# Synthesis of Functionalized Pyrrolidinone Scaffolds via Smiles-Truce Cascade

**DOI:** 10.1021/acs.orglett.3c02559

**Published:** 2023-09-05

**Authors:** Thomas Sephton, Jonathan M. Large, Sam Butterworth, Michael F. Greaney

**Affiliations:** † School of Chemistry, University of Manchester, Manchester M13 9PL, U.K.; ‡ Accelerator Building, Open Innovation Campus, LifeArc, Stevenage SG1 2FX, U.K.; § Division of Pharmacy and Optometry, School of Health Sciences, Manchester Academic Health Sciences Centre, University of Manchester, Manchester M13 9PL, U.K.

## Abstract

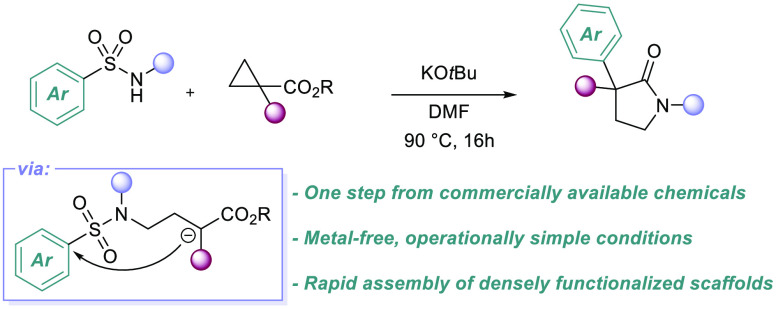

Arylsulfonamides have been found to react with cyclopropane diesters under simple base treatment to give α-arylated pyrrolidinones. This one-pot process comprises three steps: nucleophilic ring-opening of the cyclopropane, reaction of the resulting enolate in a Smiles-Truce aryl transfer, and lactam formation. The reaction represents a new, operationally simple approach to biologically active pyrrolidinones and expands Smiles-Truce arylation methods to encompass sp^3^ electrophilic centers in cascade processes.

The pyrrolidinone heterocycle is present throughout chemistry and the natural world, featured as a privileged pharmacophore in numerous pharmacologically active compounds (Scheme 1A).^1^ As such, synthetic methods to construct simple pyrrolidinone backbones are myriad (Scheme 1B), with manipulation of the *a*-position achieved by simple base treatment followed by quenching with electrophiles.^2^ These sequences, albeit robust and modular, can only be achieved in a minimum of three synthetic steps, leaving much room for improvement in terms of synthetic ideality.^3^ We thus looked to develop a more ideal synthesis of these polyfunctionalized scaffolds, achievable from commercially available, accessible starting materials while aiming to preserve the operational simplicity, robustness, and modularity of the classical approaches. Our proposal was to react a bifunctional sulfonamide with an activated cyclopropane, kickstarting a domino sequence featuring a novel 6-*exo-trig* Smiles-Truce rearrangement as the key step (Scheme 1C).

**Scheme 1:**
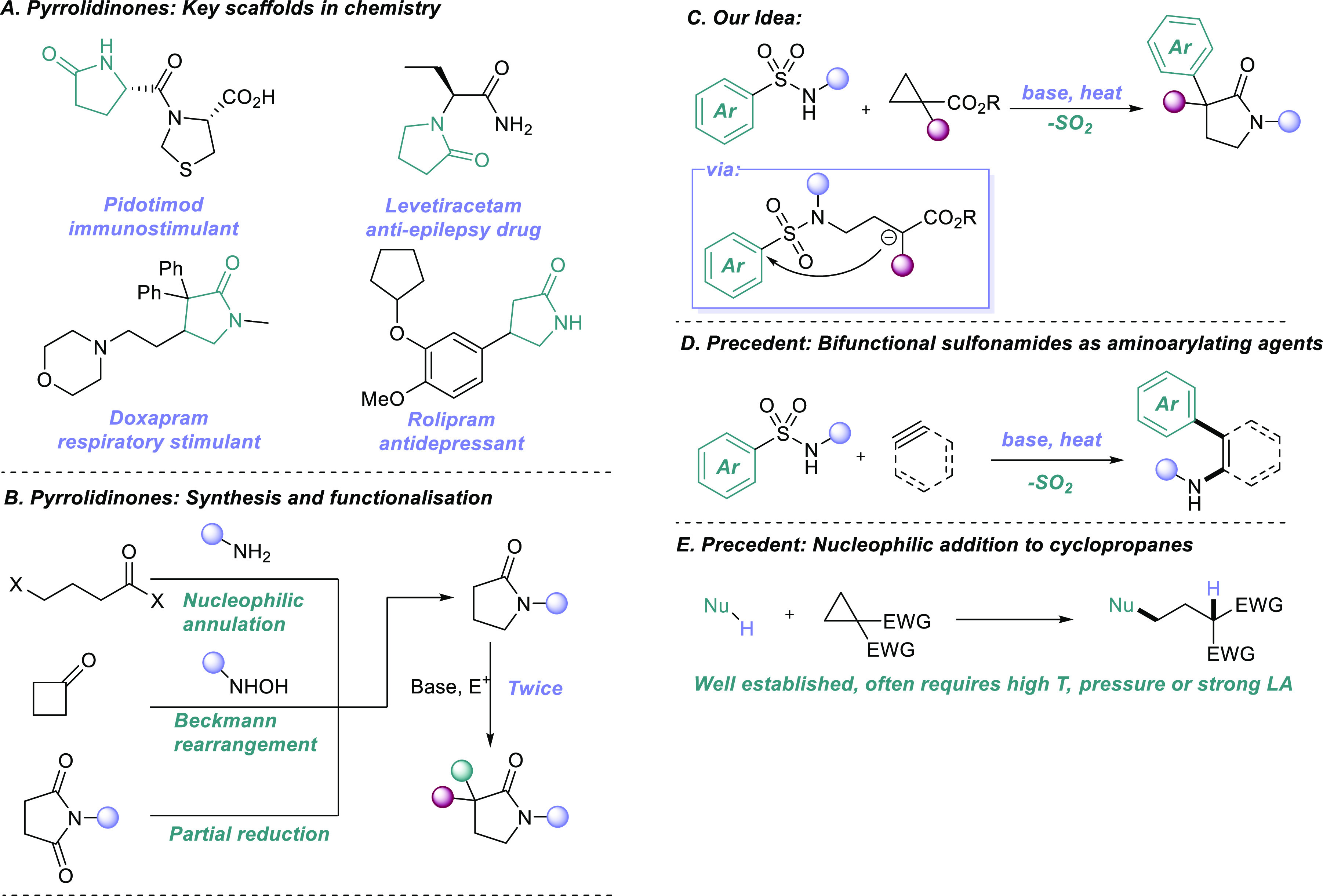
Smiles Approach to Pyrrolidinones

The Smiles rearrangement has undergone a renaissance in recent years, emerging as an efficient, transition-metal-free method to achieve difficult and valuable arylations.^4,5^ Domino Smiles transformations are particularly effective in this regard, as they generate the key anion or radical intermediate for arene transfer through an *in situ* bond-forming step. We, and others, have shown that readily available arylsulfonamides are powerful amino-arylating agents in this reaction mode, forming both C–N and C–C_(aryl)_ bonds under simple conditions through reaction with appropriate electrophiles.^6^ This reactivity has been established primarily for sulfonamide addition to sp and sp^2^ π electrophiles such as arynes, alkynes, and alkenes (Scheme 1D).^7^ Addition to a cyclopropane would represent a novel path to harnessing sp^3^ carbon centers in tandem Smiles chemistry, with the potential to access an important class of biologically active aza-heterocycle. Literature routes to arylating pyrrolidinones typically involve alkali metal enolates reacting with aryl halides through Pd(0) catalysis or S_N_Ar pathways.^8^

The nucleophilic ring opening and subsequent annulation of activated cyclopropanes have been extensively researched over the past 30 years, and a broad range of nucleophiles is able to enact this transformation. However, many of these methods require high temperatures, high pressures, strong bases, or most commonly lanthanide Lewis acid catalysis (Scheme 1E).^9^ A key question to answer at the outset, therefore, was whether an electron poor arylsulfonamide could successfully open a cyclopropane under the mild reaction conditions that characterize Smiles arylation systems.

We chose nosylamine **1a** and cyclopropane diester **2a** as our substrates and began by trialing a reaction with carbonate bases under moderate heat. We were pleased to find that sulfonamide **1a** did, in fact, react with **2a** under simple K_2_CO_3_ treatment at 70 °C in DMF, producing pyrrolidinone **3a** in low yield (Table 1, entry 1). Optimization through standard base and solvent screening led to small improvements (entries 2–4) but highlighted the difficulty of initial addition, with unconsumed sulfonamide being observed throughout. Slightly increasing the temperature proved more effective, as was increasing the concentration of the reaction, which gave yields in the mid-40s (entries 5–7). Interestingly, incorporation of Lewis acid catalysts did not improve the efficiency of the reaction (entry 8). With these improved conditions in hand, we examined the substrate scope of the reaction with a view to finding substrates that can more effectively exploit this reactivity window (Scheme 2).

**Table 1:**
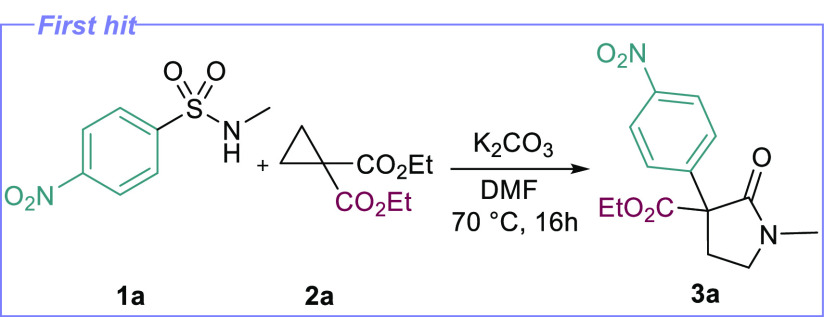
Reaction Optimization

Entry^a^	**1a** eq	Base (eq)	Solvent^b^	Conditions	% Yield
1	1	K_2_CO_3_ (1.5)	DMF	70 °C, 16h	13
2	2	Cs_2_CO_3_ (2)	DMF	70 °C, 16h	24
3	2	KO*t*Bu (2)	DMF	70 °C, 16h	21
4	2	Cs_2_CO_3_ (2)	DMSO	70 °C, 16h	20
5	3	KO*t*Bu (3)	DMF	90 °C, 72h	40
6	4	KO*t*Bu (4)	DMF	90 °C, 16h	44
**7**	**3**	**KO***t***Bu(3)**	**DMF(1M)**	**90 °C, 16h**	**48(43)**
8^c^	3	KO*t*Bu (3)	DMF	90 °C, 16h	21

a NMR yields, isolated yields in parentheses, reactions run on a 0.1 mmol scale.

b 0.1 M concentration.

c 10 mol % Yb(OTf)_3_ added.

**Scheme 2:**
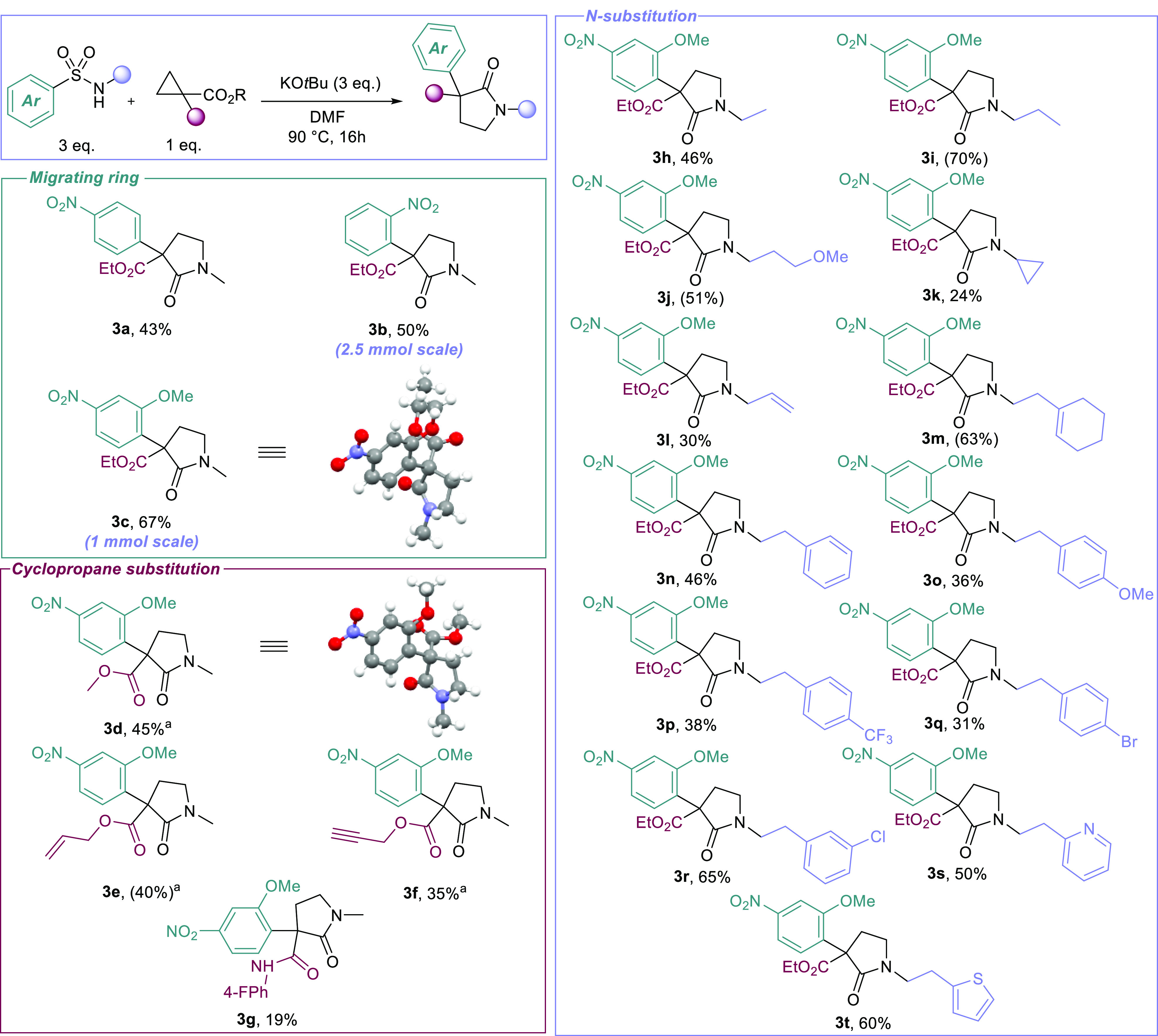
Substrate Scope Conditions used: Cs_2_CO_3_, DMF, 70 °C, 16 h. Reactions run on a 0.2 mmol scale, isolated yields, NMR yields are in parentheses.

We first examined the substrate scope of the migrating ring. Outside of our parent substrate **3a**, the reaction proceeded similarly with *ortho*-nitro substitution (**3b**), which could be effectively scaled-up (2.5 mmol). A breakthrough came when we trialed the *ortho*-methoxy *para*-nitro (*o*MeN) arene unit which worked effectively in the reaction to give **3c** in good yield (product structure confirmed by X-ray crystallographic analysis). This result is consistent with prior work in the group, further showcasing the *o*MeN unit as an ideal migrating ring in anionic Smiles systems. Efforts to replace the nitroarene with other electron-deficient arenes and heteroarenes were not successful (see the SI for more information). This is partly mitigated by the versatility of the nitro groups as a functional handle (*vide infra*). After sufficient exploration of the migrating ring scope, we looked to explore the effects of alternatively substituted cyclopropanes, taking forward *o*MeN sulfonamide as our model substrate. Substitution of the ethyl esters with methyl esters was successful, affording product **3d**, which was confirmed by X-ray crystallographic analysis. Similarly successful were allyl and propargyl esters **3e** and **3f**, the latter of which features a “clickable” alkyne handle. Incorporation of an *N*-aryl amide (**3g**) was less successful, however, as to be expected due to the reduction in electrophilicity.

Upon completion of the cyclopropane scope, we then looked to examine the tolerance of the system to sulfonamide N-substitution. Unsurprisingly, simple alkyl substitution was well tolerated, alongside simple allyl ethers affording **3h**–**3j** in good yields. Less successful was cyclic alkyl substitution, with cyclopropane **3k** being produced in only modest yield. Allyl protected sulfonamides were tolerated, producing **3l**, and internal alkenes (**3m**) were also feasible in the reaction.

Furthermore, the reaction proved effective for simple phenethyl substitution (**3n**), which, due to the relative ease of purification, was used as a scaffold to explore the broader functional group tolerance of the system. The substrate scope encompassed methoxy and trifluoromethyl substitution (**3o**–**3p**), alongside being tolerant of aryl halides, producing pyrrolidinones **3q** and **3r** and, importantly, showcasing this system’s orthogonality to transition-metal catalysis. Gratifyingly, heteroarenes were also very well tolerated in the reaction, furnishing pyridine and thiophene containing scaffolds **3s** and **3t** in good yield.

We then looked to further manipulate these pyrrolidinone scaffolds (Scheme 3A). We prepared 958 mg of our model substrate **3c**, and we first looked to cleave the ester group. This worked well, producing aryl pyrrolidinone **4a** and liberating a carbon center for further functionalization. We next sought to demonstrate the versatility of nitroarenes as functional handles, thus mitigating our substrate scope limitation. Initially, we simply reduced the nitroarene to an aniline (**4b**), unveiling a privileged functional handle. Next, we looked to translate some of Nakao’s work to our system, utilizing nitroarenes as pseudo halides for Pd-catalyzed cross-couplings.^10^ We were pleased to find that we could enact a nitro-Suzuki on our model substrate, furnishing biaryl pyrrolidinone **4c** in good yields. Similarly, we could reductively denitrate our compound in excellent yield, producing compound **4d** and thus effectively mitigating our migrating ring scope limitations.

**Scheme 3:**
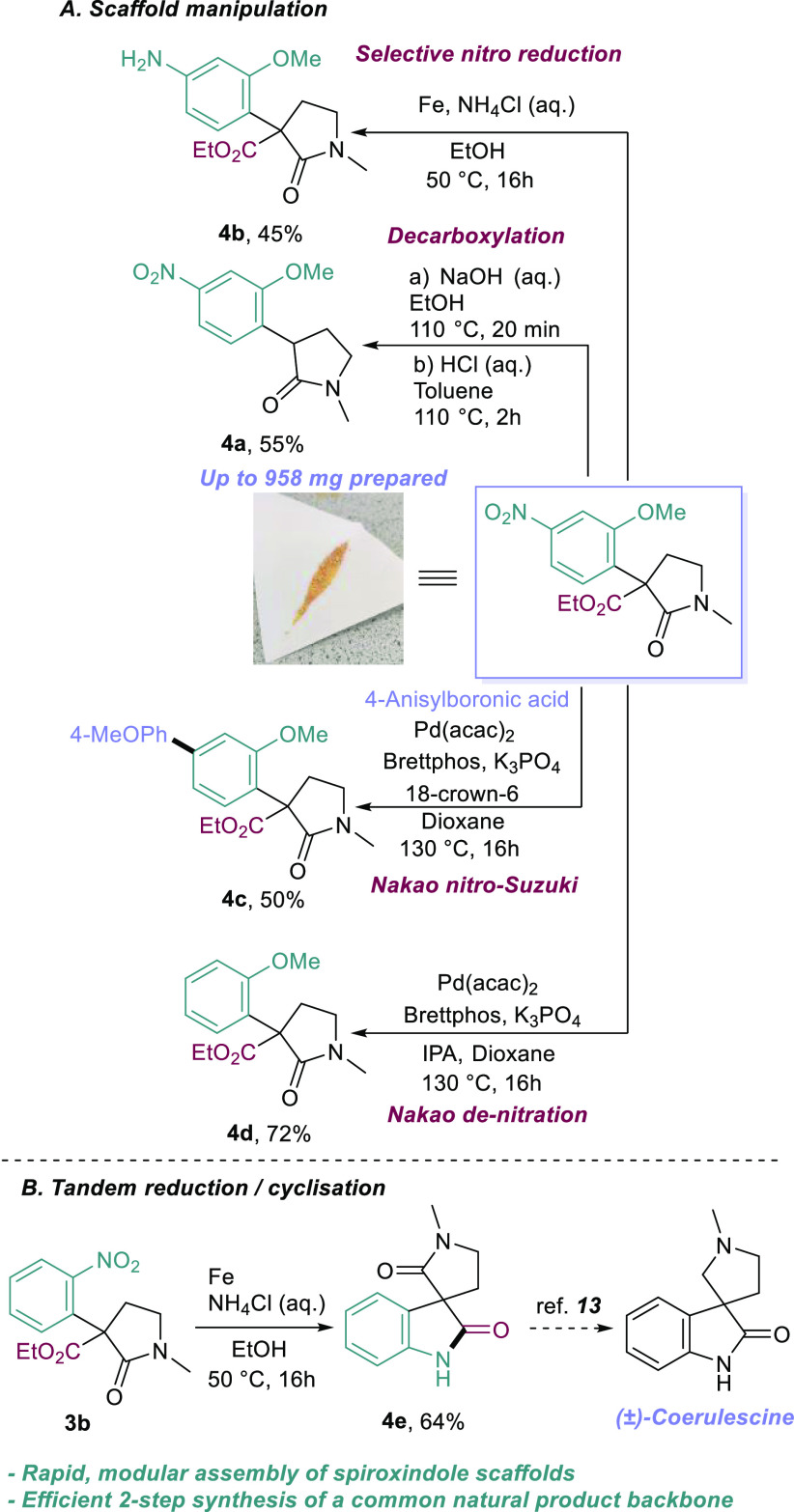
Further Manipulation

After successfully manipulating our model substrate, we proposed that simple reduction of *ortho*-nitro example **3b** would enact an *in situ* cyclization (Scheme 3B), thus, providing modular, efficient, and rapid access to spiroxindole scaffolds, which are key pharmacophores in pharmaceuticals and the backbone of many natural products.^11,12^ The reaction worked well, affording spiroxindole **4e** in good yield. **4e** is a direct precursor to natural products (±)-Coerulescine and (±)-Horsfiline, which can be afforded in a few simple steps.^13^

Finally, we elucidated the mechanism of the reaction. We subjected an electron-rich sulfonamide to the standard reaction conditions (Scheme 4A) and obtained ring-opened adduct **5a** as the only observed product. This result confirmed our proposed initial step of the reaction and that these sulfonamides can open cyclopropanes in the absence of strong Lewis acids, high temperatures, or strong bases. With this knowledge in hand, we propose that sulfonamide **1** initially attacks cyclopropane **2**, forming intermediate **A**, which can undergo a *6-exo trig* Smiles-Truce rearrangement *via* Meisenheimer intermediate **B**, extruding SO_2_ and liberating reactive amine **C**, which immediately cyclizes to form pyrrolidinone **3** (Scheme 4B).

**Scheme 4:**
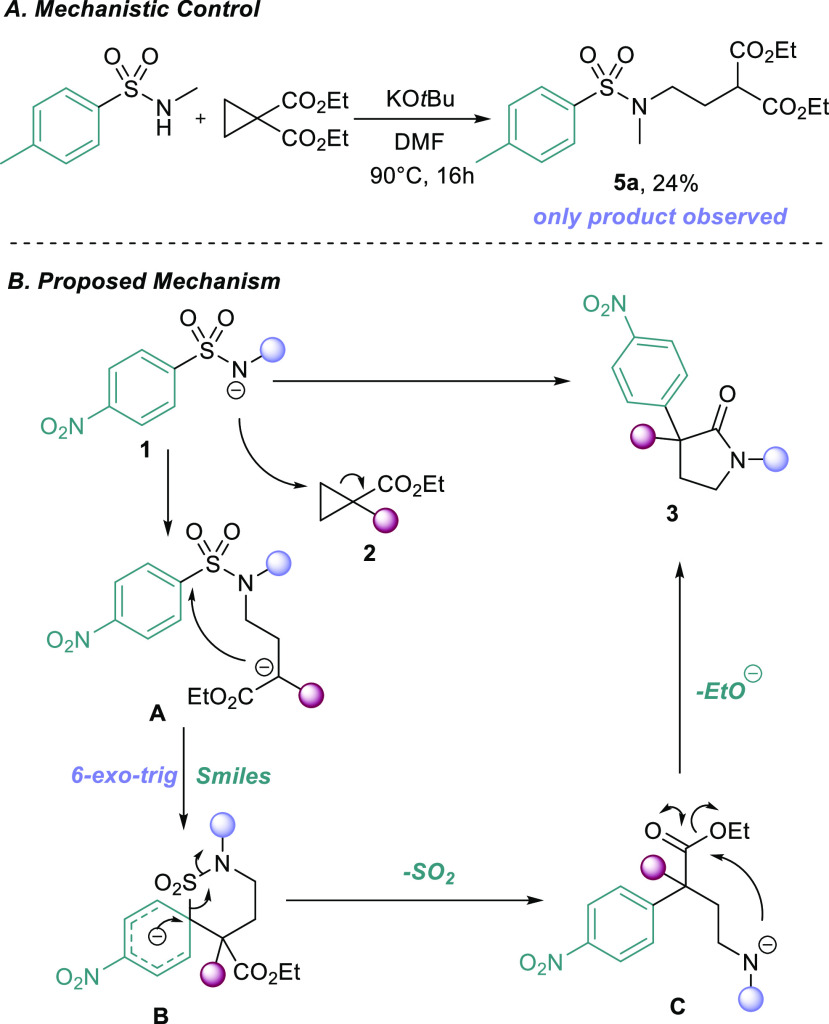
Mechanistic Investigations

In conclusion, we developed a novel method for the one-step synthesis of densely functionalized pyrrolidinones. The reaction is metal-free, proceeds from widely commercially available starting materials, and is operationally simple, needing only a simple base treatment and heating. The reaction is scalable, and the products formed can be easily diversified and further functionalized, affording rapid access to valuable pharmacophore structures in as little as two steps.

## Data Availability

The data underlying this study are available in the published article and its Supporting Information.

## Supporting Information Available

Synthetic methods, optimization studies, preparative procedures, mechanistic studies and ^1^H NMR, ^13^C NMR, ^19^F NMR spectra, HRMS, and melting points (PDF)
