# Supplementary material for: Synthesis of Functionalized Pyrrolidinone Scaffolds via Smiles-Truce Cascade
Source: Org Lett. 2023 Sep 5;25(36):6736–40. doi: 10.1021/acs.orglett.3c02559 (PMC10510726; doi:10.1021/acs.orglett.3c02559)
Supplement: Supplementary file 1 — ol3c02559_si_001.pdf [file ol3c02559_si_001.pdf]

Synthesis of functionalized pyrrolidinone scaffolds *via* Smiles-Truce cascade

Thomas Sephton,[1] Jonathan M. Large,[2] Sam Butterworth[3]* and Michael F. Greaney[1]*

[1] School of Chemistry, University of Manchester, Manchester M13 9PL, UK.

[2] LifeArc, Accelerator Building, Open Innovation Campus, Stevenage SG1 2FX, UK.

[3] Division of Pharmacy and Optometry, School of Health Sciences, Manchester Academic Health Sciences Centre, University of Manchester, Manchester M13 9PL, UK.

sam.butterworth@manchester.ac.uk

michael.greaney@manchester.ac.uk

Supporting Information

Contents

1. General Remarks S-3
2. General Procedures S-5
3. Selected Limitations S-7
4. X-Ray Crystallography S-8
5. Compound Characterisation Data S-13
6. NMR Spectra S-35
7. References S-79

1. General Remarks

All air and/or moisture sensitive reactions were performed under an atmosphere of dry nitrogen using anhydrous solvents and standard *Schlenk* techniques. The glassware used for such reactions was oven-dried. Reagents and solvents were purchased from commercial sources at the highest available grade and used as supplied unless otherwise noted. Small amounts of liquids were handled using 50, 100 and 250 µL Hamilton® 700 Series, 800 Series and GASTIGHT PTFE Luer-lock 1700 Series microsyringes, fit with Luer-lock needles. Flash column chromatography was performed using re-used 10g, 25g or 50g Biotage® Snap Ultra or Biotage Sfär Silica cartridges on a Biotage Isolera Four automated column, using 35-70 µm, 60 Å silica gel for chromatography from ThermoFisher Scientific® or 40-63 µm 60 Å silica gel from Sigma-Aldrich.

^{1}H, ^{13}C and ^{19}F NMR spectroscopy were recorded on either 500 MHz (Bruker® AVII+ 500, Bruker AVIII HD 500) or 400 MHz (Bruker AVIII HD 400, Bruker AVIII 400) NMR spectrometers. Chemical shifts (δ) are reported in parts per million (ppm) and multiplicities are reported as singlets (s), broad singlets (brs), doublets (d), triplets (t), quartets (q), pentets (p), sextets (sx), heptets (h), combinations thereof (dt meaning a doublet of triplets), or multiplets (m). Coupling constants (J) are reported in Hertz (Hz). All ^{1}H NMR and ^{13}C NMR shifts were referenced to the residual solvent peak of $CDCl_3$ (^{1}H referenced to 7.26 ppm and ^{13}C referenced to 77.16 ppm). All ^{19}F chemical shifts were unadjusted from raw data. 2D heteronuclear single quantum coherence (HSQC), heteronuclear multiple bond correlation (HMBC) and homonuclear correlation spectroscopy (COSY) NMR spectroscopy were used to assist the assignment of signals. NMR yields were calculated from ^{1}H NMR spectra using nitromethane as an internal standard.

Mass spectrometry measurements were carried out by the Mass Spectrometry Service in the Department of Chemistry at the University of Manchester (experiments and analysis was carried out by Gareth Smith, Emma Enston, Otis Leahair and Carole Webb). High resolution mass spectrometry (HRMS) was recorded on ThermoFisher Scientific Q-Exactive™, Thermo Scientific Exactive plus EMR and Agilent 6530 Q-TOF instruments, using either electrospray ionisation (ESI), atmospheric-pressure chemical ionisation (APCI) - in some cases APCI was carried out using an atmospheric solids analysis probe (ASAP) - as ionisation methods in the positive and negative mode. Low resolution APCI was recorder on Agilent® 6120 Quadrupole LC/MS or a ThermoFisher Scientific Q-Exactive instruments in the positive and negative mode. Melting points (mp) were recorded on a Griffin melting point apparatus to the nearest degree and are uncorrected.

Compound names are those generated by ChemDraw® (PerkinElmer®) following International Union of Pure and Applied Chemistry (IUPAC) nomenclature. Novel compounds are labelled in *italics*.

2. General Procedures

General Procedure A: Sulfonamide synthesis

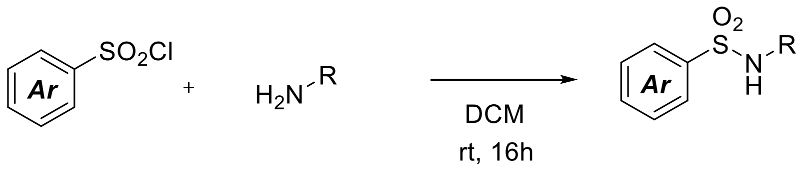

A 100 mL round-bottom flask was charged with the corresponding aryl sulfonyl chloride (1 eq.), evacuated and then backfilled with nitrogen. DCM (0.08 M) and the corresponding amine (2.5 eq.) were then added *via* syringe. The resulting solution was stirred at room temperature overnight. After this, the mixture was diluted with DCM and washed with aq. HCl (1 M). The organic layer was dried with anhydrous $MgSO_4$, filtered and concentrated *in vacuo*. If necessary, the crude product was then purified using flash column chromatography.

General Procedure B: Carboxylic acid alkylation

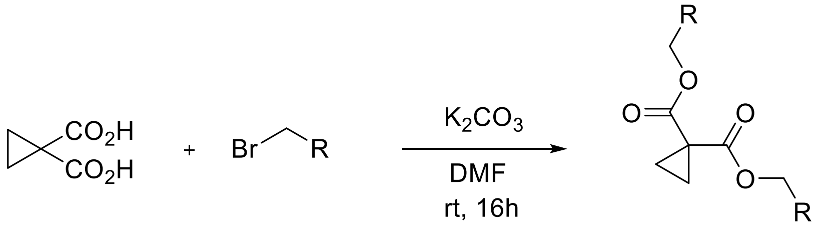

Prepared by adaptation of a literature procedure.[1] A 2-5 mL microwave vial was charged with carboxylic acid (270 mg, 2 mmol) and K_2CO_3 (2.8 g, 20 mmol). The vial was sealed, evacuated and backfilled with nitrogen. DMF (2 mL) and the corresponding alkyl bromide (12 mmol) were added *via* syringe. The resulting mixture was stirred at room temperature overnight. After this, the vial was unsealed, and the mixture was diluted with ethyl acetate and washed with 10% aq. LiCl. The organic layer was then dried with anhydrous $MgSO_4$, filtered and concentrated *in vacuo*.

General Procedure C: Pyrrolidinone synthesis (Conditions A)

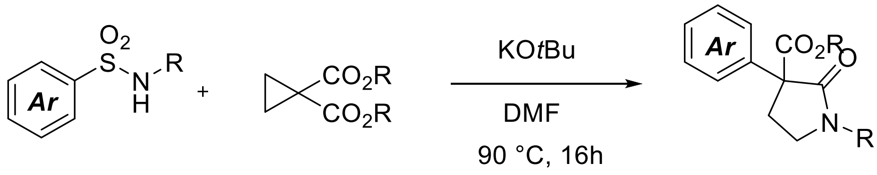

A 2-5 mL oven-dried microwave vial was charged with the corresponding sulfonamide (0.6 mmol) and KO*t*Bu (68 mg, 0.6 mmol). The vial was sealed, evacuated and backfilled with nitrogen. DMF (0.2 mL)

and the corresponding cyclopropane (0.2 mmol) were then added *via* syringe. The resulting mixture was heated in a heating mantle to 90°C and stirred overnight. The vial was unsealed, and the mixture was diluted with ethyl acetate. The organic phase was washed with aq. HCl (1 M) and LiCl (10 %), dried ($MgSO_4$), filtered and concentrated *in vacuo*. The crude product was then purified using flash column chromatography.

General Procedure D: Pyrrolidinone synthesis (Conditions B)

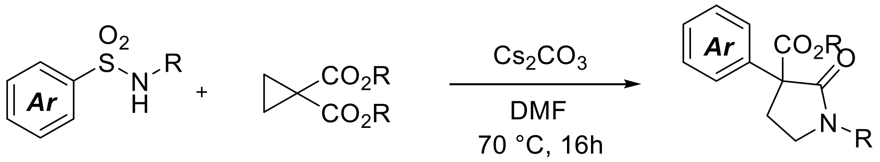

A 2-5 mL oven-dried microwave vial was charged with the corresponding sulfonamide (0.6 mmol) and Cs_2CO_3 (195 mg, 0.6 mmol). The vial was sealed, evacuated and backfilled with nitrogen. DMF (2 mL) and the corresponding cyclopropane (0.2 mmol) were then added *via* syringe. The resulting mixture was heated in a heating mantle to 70°C and stirred overnight. The vial was unsealed, and the mixture was diluted with ethyl acetate. The organic phase was washed with aq. HCl (1 M) and LiCl (10 %), dried ($MgSO_4$), filtered and concentrated *in vacuo*. The crude product was then purified using flash column chromatography.

General Procedure E: Nitroarene reduction

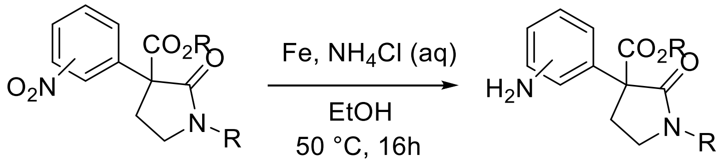

A 2-5 mL oven-dried microwave vial was charged with the corresponding nitroarene **3** (1 eq.), ammonium chloride (0.7 eq.), Fe filings (5 eq.) and water (0.33 M). EtOH (0.5 M) was added *via* syringe and the resulting suspension was heated in a heating mantle and stirred at 50 °C for 16 h. The reaction mixture was cooled to room temperature and filtered. Water was added and the aqueous layer was extracted with ethyl acetate. The combined organic layers were washed with brine, dried over anhydrous $MgSO_4$, filtered, and concentrated *in vacuo*. The crude product was then purified by flash column chromatography.

3. Selected Limitations

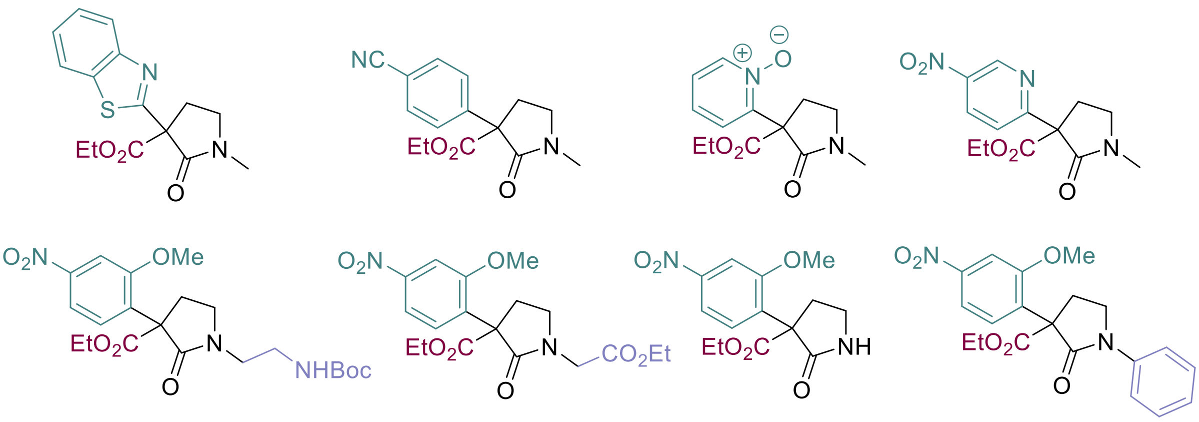

4. X-Ray Crystallography

All data collections, crystal structure determinations and refinements were done by the X-ray crystallography service (George F. S. Whitehead) at The University of Manchester.

Data collection

X-ray data was collected at a temperature of 100 K on a Rigaku® FR-X DW rotating anode diffractometer using CuKα radiation, (λ = 1.54184 Å) with an AFC-11 RINC goniometer and a Rigaku Hypix 6000 HE photon counting detector. The diffractometer was equipped with an Oxford Cryosystems® Cryostream 800 plus nitrogen flow gas system.

Crystal structure determinations and refinements

X-ray data were processed and reduced using CrysAlis^{Pro} suite of programs. The crystal structures were solved and refined against all F2 values using the SHELX and Olex 2 suite of programs. All the non-hydrogen atoms were refined anisotropically. Hydrogen atoms were placed in a calculated position refined using idealised geometries (riding model) and assigned fixed isotropic displacement parameters.

Data availability

Crystallographic data for **3c** and **3d** have been deposited in the Cambridge Crystallographic Data Centre, with deposition numbers CCDC 2277260 (**3c**), 2277255 (**3d**), and are available free of charge via https://www.ccdc.cam.ac.uk/structures/.

X-ray structure of *ethyl 3-(2-methoxy-4-nitrophenyl)-1-methyl-2-oxopyrrolidine-3-carboxylate (3c)*

CCDC Deposition Number: 2277260

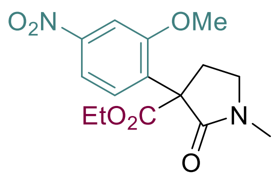

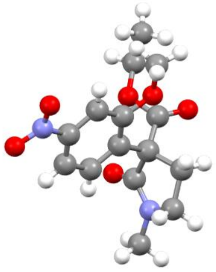

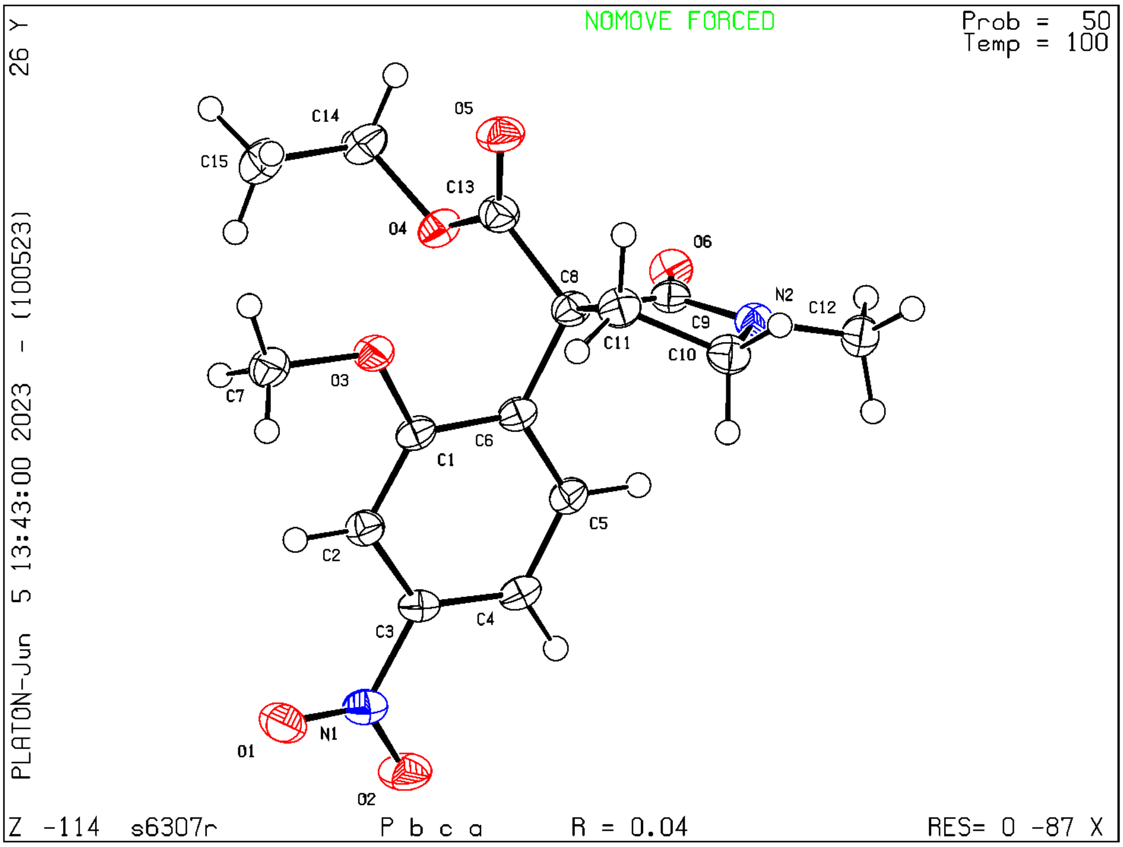

Empirical formula	$C_{15}H_{18}N_2O_6$
Formula weight	322.31
Temperature (K)	100
Space group	P 1 21 1

a (Å)	13.3719 (3)
b (Å)	6.83663(18)
c (Å)	32.6070(8)
α (°)	90
β (°)	90
γ (°)	90
Volume ($Å^3$)	2980.89(13)
Z	8
P_{calc} (g/cm^3)	1.436
μ (mm^{-1})	0.947
F(000)	1360.0
Radiation (Å)	CuKα (λ = 1.54184)
Independent reflections	3079
Data/parameters	3079/211
Goodness-of-fit on F^2	1.056
Final R indexes [I>=2σ (I)]	wR_2 = 0.1138
Final R indexes [all data]	R_1 = 0.0425
Ellipsoid contour probability levels (%)	50

Table S1. Crystal data and structure refinement for 3c.

X-ray structure of *methyl 3-(2-methoxy-4-nitrophenyl)-1-methyl-2-oxopyrrolidine-3-carboxylate (3d)*

CCDC Deposition Number: 2277255

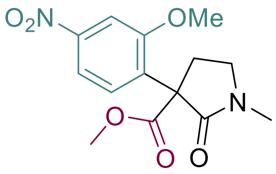

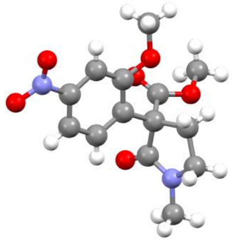

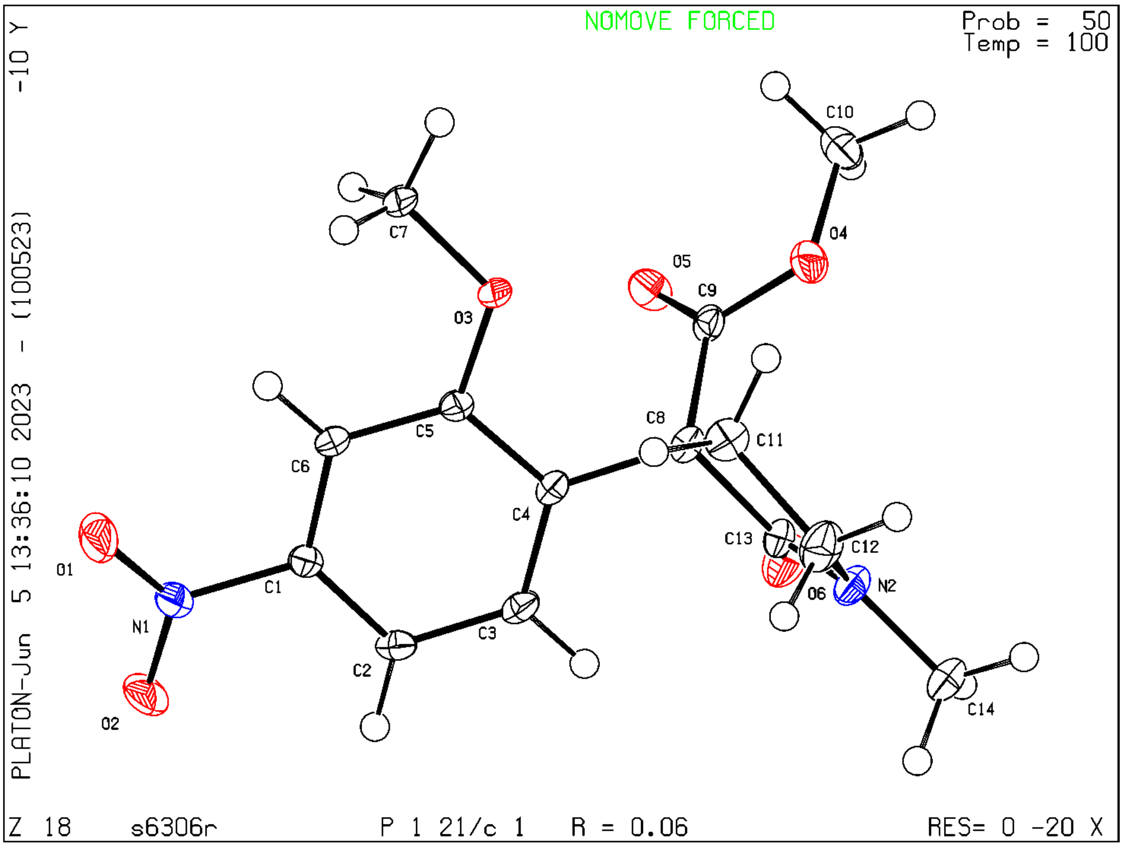

Empirical formula	$C_{14}H_{16}N_2O_6$
Formula weight	308.29
Temperature (K)	100
Space group	P 1 21/c 1

a (Å)	11.4507 (5)
b (Å)	18.2000 (9)
c (Å)	6.6863 (3)
α (°)	90
β (°)	91.583 (5)
γ (°)	90
Volume ($Å^3$)	1392.91 (11)
Z	4
P_{calc} (g/cm^3)	1.470
μ (mm^{-1})	0.116
F(000)	648.0
Radiation (Å)	CuKα (λ = 0.71073)
Independent reflections	3479
Data/parameters	3479/202
Goodness-of-fit on F^2	1.089
Final R indexes [I>=2σ (I)]	wR_2 = 0.1622
Ellipsoid contour probability levels (%)	50

Table S2. Crystal data and structure refinement for 3d.

5. Compound Characterisation Data

N-methyl-2-nitrobenzenesulfonamide (1b)

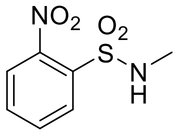

Prepared according to general procedure A (10 mmol scale). The pure product was afforded as an off-white solid (1.62 g, 75% yield).

^{1}H NMR (500 MHz, CDCl$_3$) δ 8.16 – 8.11 (m, 1H), 7.88 – 7.84 (m, 1H), 7.78 – 7.72 (m, 2H), 5.24 (s, 1H), 2.79 (d, *J* = 5.4 Hz, 3H).

^{13}C NMR (126 MHz, CDCl$_3$) δ 148.4, 133.8, 132.8, 132.5, 131.6, 125.6, 29.9.

HRMS (APCI) m/z: $[M+H]^+$ Calculated for $[C_7H_9O_4N_2S]^+$ 217.0278; found 217.0281.

The data are in accordance with literature.[2]

2-methoxy-N-methyl-4-nitrobenzenesulfonamide (1c)

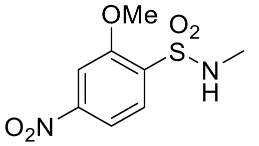

Prepared according to general procedure A (10 mmol scale). The pure product was afforded as an off-white solid (2.15 g, 88% yield).

^{1}H NMR (500 MHz, CDCl$_3$) δ 8.11 (d, *J* = 8.5 Hz, 1H), 7.94 (d, *J* = 8.4 Hz, 1H), 7.90 – 7.87 (m, 1H), 4.95 – 4.88 (m, 1H), 4.11 (d, *J* = 1.4 Hz, 3H), 2.65 (d, *J* = 5.3 Hz, 3H).

^{13}C NMR (126 MHz, CDCl$_3$) δ 156.9, 151.7, 132.3, 132.0, 115.7, 107.4, 57.4, 29.5.

HRMS (APCI) m/z: $[M+H]^+$ Calculated for $[C_8H_{11}O_5N_2S]^+$ 247.0383; found 247.0386.

The data are in accordance with the literature.[2]

N-ethyl-2-methoxy-4-nitrobenzenesulfonamide (1h)

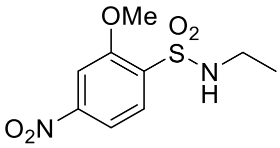

Prepared according to general procedure A (2 mmol scale). The pure product was afforded as a yellow solid (400 mg, 77% yield).

¹H NMR (500 MHz, $CDCl_3$) δ 8.11 (d, *J* = 8.5 Hz, 1H), 7.93 (dd, *J* = 8.5, 2.0 Hz, 1H), 7.88 (d, *J* = 2.1 Hz, 1H), 4.92 (t, *J* = 6.1 Hz, 1H), 4.11 (s, 3H), 3.02 (qd, *J* = 7.2, 5.9 Hz, 2H), 1.10 (t, *J* = 7.2 Hz, 3H).

¹³C NMR (126 MHz, $CDCl_3$) δ 156.8, 151.6, 133.8, 131.5, 115.7, 107.5, 57.3, 38.7, 15.3.

HRMS (APCI) m/z: $[M+H]^+$ Calculated for $[C_9H_{13}O_5N_2S]^+$ 261.0540; found 261.0546.

mp: 138-140 °C.

2-methoxy-4-nitro-N-propylbenzenesulfonamide (1i)

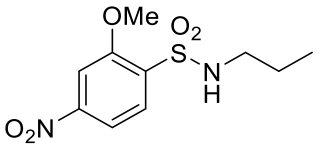

Prepared according to general procedure A (2 mmol scale). The pure product was afforded as a yellow solid (495 mg, 90% yield).

¹H NMR (500 MHz, $CDCl_3$) δ 8.10 (d, J = 8.5 Hz, 1H), 7.93 (dd, J = 8.5, 2.0 Hz, 1H), 7.88 (d, J = 2.1 Hz, 1H), 5.03 (t, J = 6.2 Hz, 1H), 4.11 (s, 3H), 2.90 (td, J = 7.1, 6.1 Hz, 2H), 1.47 (q, J = 7.3 Hz, 2H), 0.86 (t, J = 7.4 Hz, 3H).

¹³C NMR (126 MHz, $CDCl_3$) δ 156.8, 151.6, 133.7, 131.5, 115.7, 107.4, 57.3, 45.4, 23.0, 11.1.

HRMS (APCI) m/z: $[M+H]^+$ Calculated for $[C_{10}H_{15}O_5N_2S]^+$ 275.0696; found 275.0701.

mp: 70-72 °C.

2-methoxy-N-(3-methoxypropyl)-4-nitrobenzenesulfonamide (1j)

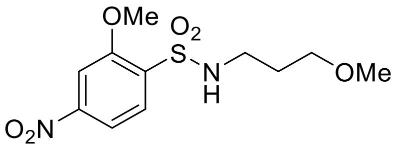

Prepared according to general procedure A (2 mmol scale). The pure product was afforded as a yellow solid (525 mg, 86% yield).

¹H NMR (400 MHz, $CDCl_3$) δ 8.08 (d, *J* = 8.5 Hz, 1H), 7.91 (dd, *J* = 8.5, 2.0 Hz, 1H), 7.87 (d, *J* = 2.0 Hz, 1H), 5.64 (t, *J* = 5.8 Hz, 1H), 4.09 (s, 3H), 3.42 (t, *J* = 5.6 Hz, 2H), 3.31 (s, 3H), 3.06 (q, *J* = 6.1 Hz, 2H), 1.74 – 1.67 (m, 2H).

¹³C NMR (101 MHz, $CDCl_3$) δ 156.9, 151.5, 133.6, 131.5, 115.6, 107.3, 71.6, 59.0, 57.2, 42.5, 29.0.

HRMS (APCI) m/z: $[M+H]^+$ Calculated for $[C_{11}H_{17}O_6N_2S]^+$ 305.0806; found 305.0802.

mp: 80-82 °C.

N-cyclopropyl-2-methoxy-4-nitrobenzenesulfonamide (1k)

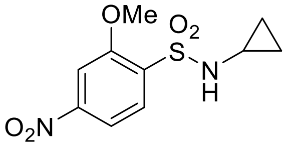

Prepared according to general procedure A (2 mmol scale). The pure product was afforded as a red solid (470 mg, 86% yield).

^{1}H NMR (500 MHz, $CDCl_3$) δ 8.17 (d, *J* = 8.5 Hz, 1H), 7.96 (dd, *J* = 8.5, 2.1 Hz, 1H), 7.90 (d, *J* = 2.0 Hz, 1H), 5.37 (s, 1H), 4.12 (s, 3H), 2.16 – 2.10 (m, 1H), 0.73 – 0.55 (m, 4H).

^{13}C NMR (126 MHz, $CDCl_3$) δ 156.9, 151.8, 133.2, 132.1, 115.8, 107.5, 57.4, 24.6, 6.4.

HRMS (APCI) m/z: $[M+H]^+$ Calculated for $[C_{10}H_{13}O_5N_2S]^+$ 273.0540; found 273.0541.

mp: 116-118 °C.

N-allyl-2-methoxy-4-nitrobenzenesulfonamide (1l)

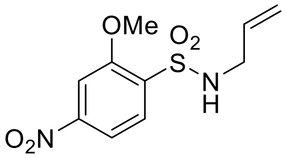

Prepared according to general procedure A (2 mmol scale). The pure product was afforded as a yellow solid (470 mg, 86% yield).

^{1}H NMR (500 MHz, $CDCl_3$) δ 8.09 (d, *J* = 8.6 Hz, 1H), 7.93 (d, *J* = 8.6 Hz, 1H), 7.88 (s, 1H), 5.67 (dddd, *J* = 17.5, 10.1, 6.7, 5.2 Hz, 1H), 5.15 (dq, *J* = 17.1, 1.5 Hz, 1H), 5.10 – 5.02 (m, 2H), 4.10 (s, 3H), 3.62 (t, *J* = 6.1, 1.6 Hz, 2H).

^{13}C NMR (126 MHz, $CDCl_3$) δ 156.8, 151.6, 133.8, 132.7, 131.4, 118.1, 115.7, 107.5, 57.3, 46.1.

HRMS (APCI) m/z: $[M+H]^+$ Calculated for $[C_{10}H_{13}O_5N_2S]^+$ 273.0540; found 273.0544.

mp: 76-78 °C.

N-(2-(cyclohex-1-en-1-yl)ethyl)-2-methoxy-4-nitrobenzenesulfonamide (1m)

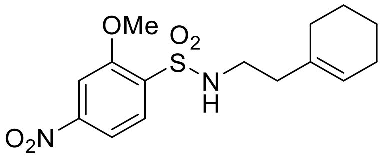

Prepared according to general procedure A (2 mmol scale). The pure product was afforded as an off-white solid (546 mg, 80% yield).

^{1}H NMR (400 MHz, CDCl$_3$) δ 8.11 (d, *J* = 8.5 Hz, 1H), 7.94 (dd, *J* = 8.5, 2.1 Hz, 1H), 7.88 (d, *J* = 2.1 Hz, 1H), 5.43 – 5.37 (m, 1H), 4.92 – 4.87 (m, 1H), 4.09 (s, 3H), 3.02 (q, *J* = 6.4 Hz, 2H), 2.09 (t, *J* = 6.6 Hz, 2H), 2.00 – 1.96 (m, 2H), 1.79 – 1.73 (m, 2H), 1.63 – 1.49 (m, 4H).

^{13}C NMR (101 MHz, CDCl$_3$) δ 156.8, 151.6, 133.6, 131.5, 124.7, 115.8, 107.4, 57.3, 41.0, 37.6, 27.8, 25.3, 22.8, 22.4.

HRMS (APCI) m/z: $[M+H]^+$ Calculated for $[C_{15}H_{21}O_5N_2S]^+$ 341.1166; found 341.1168.

mp: 112-114 °C.

2-methoxy-4-nitro-N-phenethylbenzenesulfonamide (1n)

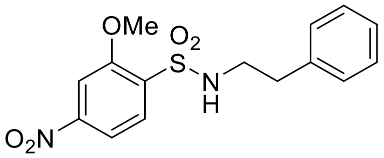

Prepared according to general procedure A (2 mmol scale). The pure product was afforded as a yellow solid (483 mg, 72% yield).

^{1}H NMR (400 MHz, CDCl$_3$) δ 8.07 (d, *J* = 8.5 Hz, 1H), 7.91 (dd, *J* = 8.5, 2.1 Hz, 1H), 7.74 (d, *J* = 2.0 Hz, 1H), 7.31 – 7.21 (m, 3H), 7.09 – 7.05 (m, 2H), 4.89 (t, *J* = 6.1 Hz, 1H), 3.75 (s, 3H), 3.22 (q, *J* = 6.3 Hz, 2H), 2.80 (t, *J* = 6.5 Hz, 2H).

^{13}C NMR (101 MHz, CDCl$_3$) δ 156.6, 151.6, 137.7, 133.1, 131.4, 129.0, 128.8, 127.2, 115.6, 107.2, 56.9, 44.5, 35.4.

HRMS (APCI) m/z: $[M+H]^+$ Calculated for $[C_{15}H_{17}O_5N_2S]^+$ 337.0853; found 337.0860.

mp: 126-128 °C.

2-methoxy-N-(4-methoxyphenethyl)-4-nitrobenzenesulfonamide (1o)

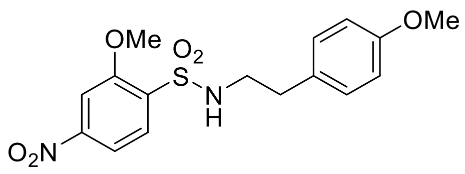

Prepared according to general procedure A (2 mmol scale). The pure product was afforded as a yellow solid (642 mg, 88% yield).

^{1}H NMR (500 MHz, CDCl$_3$) δ 8.05 (d, *J* = 8.5 Hz, 1H), 7.90 (dd, *J* = 8.5, 2.0 Hz, 1H), 7.74 (d, *J* = 2.1 Hz, 1H), 7.00 – 6.96 (m, 2H), 6.81 – 6.77 (m, 2H), 4.90 (t, *J* = 6.1 Hz, 1H), 3.80 (s, 3H), 3.78 (s, 3H), 3.18 (q, *J* = 6.4 Hz, 2H), 2.73 (t, *J* = 6.5 Hz, 2H).

^{13}C NMR (126 MHz, CDCl$_3$) δ 158.7, 156.6, 151.5, 133.1, 131.3, 129.8, 129.5, 115.6, 114.3, 107.2, 56.9, 55.4, 44.8, 34.6.

HRMS (APCI) m/z: $[M]^+$ Calculated for $[C_{16}H_{18}O_6N_2S]$ 366.0880; found 366.0887.

mp: 126-128 °C.

2-methoxy-4-nitro-N-(4-(trifluoromethyl)phenethyl)benzenesulfonamide (1p)

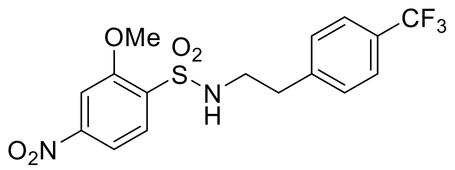

Prepared according to general procedure A (2 mmol scale). The pure product was afforded as an off-white solid (642 mg, 79% yield).

^{1}H NMR (500 MHz, CDCl$_3$) δ 8.06 (d, *J* = 8.5 Hz, 1H), 7.91 (dd, *J* = 8.5, 2.1 Hz, 1H), 7.78 (d, *J* = 2.0 Hz, 1H), 7.53 (d, *J* = 8.0 Hz, 2H), 7.23 (d, *J* = 8.0 Hz, 2H), 5.02 (t, *J* = 6.2 Hz, 1H), 3.86 (s, 3H), 3.25 (q, *J* = 6.6 Hz, 2H), 2.88 (t, *J* = 6.7 Hz, 2H).

^{13}C NMR (126 MHz, CDCl$_3$) δ 156.6, 151.6, 141.9, 133.1, 131.3, 129.4 (d, *J* = 32.4 Hz), 129.2, 125.7 (q, *J* = 3.8 Hz), 124.1 (d, *J* = 271.9 Hz), 115.7, 107.3, 57.0, 44.2, 35.7.

^{19}F NMR (471 MHz, CDCl$_3$) δ -62.52.

HRMS (APCI) m/z: $[M+H]^+$ Calculated for $[C_{16}H_{16}O_5N_2SF_3]^+$ 405.0727; found 405.0731.

mp: 120-122 °C.

N-(4-bromophenethyl)-2-methoxy-4-nitrobenzenesulfonamide (1q)

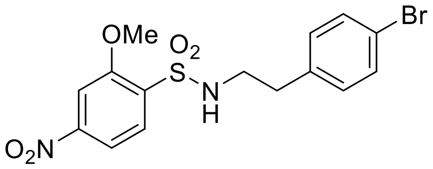

Prepared according to general procedure A (2 mmol scale). The pure product was afforded as a yellow solid (665 mg, 80% yield).

^{1}H NMR (400 MHz, CDCl$_3$) δ 8.05 (d, *J* = 8.5 Hz, 1H), 7.92 (dd, *J* = 8.5, 2.0 Hz, 1H), 7.78 (d, *J* = 2.0 Hz, 1H), 7.42 – 7.36 (m, 2H), 6.99 – 6.92 (m, 2H), 4.88 (t, *J* = 6.1 Hz, 1H), 3.86 (s, 3H), 3.21 (q, *J* = 6.5 Hz, 2H), 2.77 (t, *J* = 6.6 Hz, 2H).

^{13}C NMR (101 MHz, CDCl$_3$) δ 156.6, 151.6, 136.7, 133.2, 132.0, 131.3, 130.5, 121.1, 115.7, 107.3, 57.1, 44.4, 35.2.

HRMS (APCI) m/z: $[M+H]^+$ Calculated for $[C_{15}H_{16}O_5N_2SBr]^+$ 414.9958; found 414.9962.

mp: 128-130 °C.

N-(3-chlorophenethyl)-2-methoxy-4-nitrobenzenesulfonamide (1r)

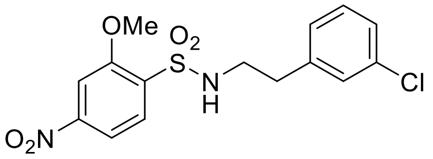

Prepared according to general procedure A (2 mmol scale). The pure product was afforded as a yellow solid (635 mg, 86% yield).

^{1}H NMR (400 MHz, CDCl$_3$) δ 8.07 (d, *J* = 8.5 Hz, 1H), 7.92 (dd, *J* = 8.5, 2.1 Hz, 1H), 7.78 (d, *J* = 2.0 Hz, 1H), 7.23 – 7.20 (m, 2H), 7.02 – 6.96 (m, 2H), 4.90 (t, *J* = 6.1 Hz, 1H), 3.86 (s, 3H), 3.26 (q, *J* = 6.4 Hz, 2H), 2.78 (t, *J* = 6.6 Hz, 2H).

^{13}C NMR (101 MHz, CDCl$_3$) δ 151.6, 139.8, 134.7, 133.2, 131.2, 130.2, 128.7, 127.3, 127.1, 115.7, 107.4, 57.0, 44.2, 35.3.

HRMS (APCI) m/z: $[M+H]^+$ Calculated for $[C_{15}H_{16}O_5N_2SCl]^+$ 371.0463; found 371.0466.

mp: 106-108 °C.

2-methoxy-4-nitro-N-(2-(pyridin-2-yl)ethyl)benzenesulfonamide (1s)

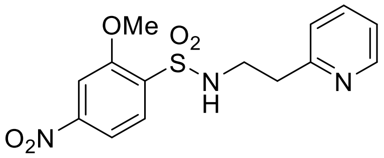

Prepared according to general procedure A (2 mmol scale). The pure product was afforded as a yellow solid (462 mg, 69% yield).

^{1}H NMR (500 MHz, CDCl$_3$) δ 8.49 (d, *J* = 4.7 Hz, 1H), 8.10 (d, *J* = 8.5 Hz, 1H), 7.90 (dd, *J* = 8.5, 2.1 Hz, 1H), 7.76 (d, *J* = 2.1 Hz, 1H), 7.59 (td, *J* = 7.6, 1.8 Hz, 1H), 7.19 – 7.13 (m, 1H), 7.05 (d, *J* = 7.8 Hz, 1H), 6.54 (t, *J* = 6.1 Hz, 1H), 3.93 (s, 3H), 3.41 (q, *J* = 6.0 Hz, 2H), 2.92 (t, *J* = 5.9 Hz, 2H).

^{13}C NMR (126 MHz, CDCl$_3$) δ 158.9, 156.8, 151.4, 149.1, 136.9, 133.9, 131.3, 123.7, 121.9, 115.5, 107.1, 56.9, 42.3, 36.1.

HRMS (APCI) m/z: [M+H]$^+$ Calculated for $[C_{14}H_{16}O_5N_3S]^+$ 338.0805; found 338.0807.

mp: 106-108 °C.

2-methoxy-4-nitro-N-(2-(thiophen-2-yl)ethyl)benzenesulfonamide (1t)

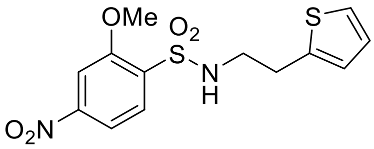

Prepared according to general procedure A (2 mmol scale). The pure product was afforded as a yellow solid (212 mg, 31% yield).

^{1}H NMR (500 MHz, CDCl$_3$) δ 8.09 (dd, *J* = 8.4, 2.4 Hz, 1H), 7.93 (dt, *J* = 8.5, 2.3 Hz, 1H), 7.79 (s, 1H), 7.18 (dt, *J* = 5.2, 1.4 Hz, 1H), 6.95 – 6.92 (m, 1H), 6.78 (s, 1H), 5.04 (t, *J* = 6.2 Hz, 1H), 3.86 (s, 3H), 3.27 – 3.19 (m, 2H), 3.02 (t, *J* = 6.4 Hz, 2H).

^{13}C NMR (126 MHz, CDCl$_3$) δ 156.7, 151.6, 140.1, 133.1, 131.3, 127.3, 126.2, 124.7, 115.7, 107.2, 57.0, 44.8, 29.9.

HRMS (APCI) m/z: [M+H]$^+$ Calculated for $[C_{13}H_{15}O_5N_2S_2]^+$ 343.0417; found 343.0421.

mp: 106-108 °C.

diallyl cyclopropane-1,1-dicarboxylate (2e)

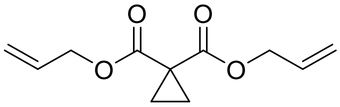

Prepared according to general procedure B (2 mmol scale). The pure product was afforded as a yellow oil (297 mg, 71% yield).

^{1}H NMR (400 MHz, $CDCl_3$) δ 5.91 (m, 2H), 5.34 (dq, *J* = 17.2, 1.5 Hz, 2H), 5.25 – 5.20 (m, 2H), 4.64 (dt, *J* = 5.6, 1.5 Hz, 4H), 1.48 (s, 4H).

^{13}C NMR (101 MHz, $CDCl_3$) δ 169.5, 131.9, 118.5, 66.1, 28.3, 16.9.

HRMS (ESI) m/z: $[M+Na]^+$ Calculated for $[C_{11}H_{14}O_4Na]^+$ 233.0784; found 233.0789.

Data are in accordance with literature.[1]

di(prop-2-yn-1-yl) cyclopropane-1,1-dicarboxylate (2f)

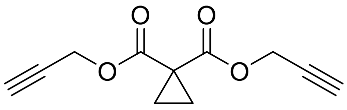

Prepared according to general procedure B (1 mmol scale). The pure product was afforded as a yellow oil (113 mg, 55% yield).

^{1}H NMR (400 MHz, $CDCl_3$) δ 4.72 (d, *J* = 2.5 Hz, 4H), 2.47 (t, *J* = 2.5 Hz, 2H), 1.53 (s, 4H).

^{13}C NMR (101 MHz, $CDCl_3$) δ 168.6, 77.2, 75.3, 53.0, 27.8, 17.4.

HRMS (APCI) m/z: $[M+H]^+$ Calculated for $[C_{11}H_{11}O_4]^+$ 207.0652; found 207.0653.

ethyl 1-methyl-3-(4-nitrophenyl)-2-oxopyrrolidine-3-carboxylate (3a)

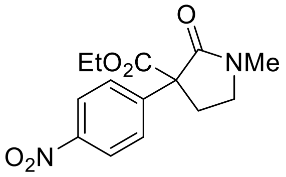

Prepared according to general procedure C (column conditions: 0-80% EtOAc in hexane). The pure product was afforded as an orange oil (25 mg, 43% yield).

^{1}H NMR (500 MHz, $CDCl_3$) δ 8.24 – 8.17 (m, 2H), 7.74 – 7.67 (m, 2H), 4.30 – 4.15 (m, 2H), 3.54 (dt, *J* = 9.7, 7.1 Hz, 1H), 3.38 – 3.30 (m, 1H), 3.11 – 3.02 (m, 1H), 2.97 (s, 3H), 2.46 – 2.36 (m, 1H), 1.22 (t, *J* = 7.1 Hz, 3H).

^{13}C NMR (126 MHz, $CDCl_3$) δ 170.0, 169.7, 147.3, 145.5, 128.7, 123.6, 62.7, 60.2, 46.6, 31.9, 30.8, 14.1.

HRMS (APCI) m/z: $[M+H]^+$ Calculated for $[C_{14}H_{17}O_5N_2]^+$ 293.1132; found 293.1130.

ethyl 1-methyl-3-(2-nitrophenyl)-2-oxopyrrolidine-3-carboxylate (3b)

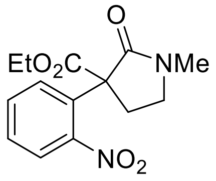

Prepared according to general procedure C (2.5 mmol scale, column conditions: 0-60% EtOAc in hexane). The pure product was afforded as a yellow solid (368 mg, 50% yield).

^{1}H NMR (400 MHz, CDCl$_3$) δ 8.07 (d, *J* = 8.2 Hz, 1H), 7.61 (t, *J* = 7.7 Hz, 1H), 7.47 (ddd, *J* = 8.6, 7.5, 1.4 Hz, 1H), 7.40 (d, *J* = 7.9 Hz, 1H), 4.21 – 4.10 (m, 2H), 3.66 – 3.58 (m, 1H), 3.51 – 3.43 (m, 1H), 3.41 – 3.33 (m, 1H), 3.04 (s, 3H), 2.29 – 2.20 (m, 1H), 1.19 (t, *J* = 7.1 Hz, 3H).

^{13}C NMR (101 MHz, CDCl$_3$) δ 170.5, 169.4, 148.6, 135.1, 133.9, 130.2, 128.6, 125.9, 62.6, 61.9, 47.0, 33.0, 30.9, 13.9.

HRMS (APCI) m/z: $[M+H]^+$ Calculated for $[C_{14}H_{17}O_5N_2]^+$ 293.1132; found 293.1129.

Data are consistent with literature.[3]

ethyl 3-(2-methoxy-4-nitrophenyl)-1-methyl-2-oxopyrrolidine-3-carboxylate (3c)

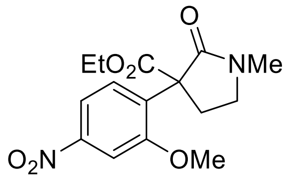

Prepared according to general procedure C (1 mmol scale, column conditions: 0-40% EtOAc in hexane). The pure product was afforded as a yellow solid (217 mg, 67% yield).

^{1}H NMR (400 MHz, CDCl$_3$) δ 7.89 – 7.75 (m, 2H), 7.71 (d, *J* = 2.2 Hz, 1H), 7.48 (d, *J* = 8.5 Hz, 1H), 4.31 – 4.21 (m, 1H), 4.15 – 4.06 (m, 1H), 3.89 (s, 3H), 3.59 – 3.50 (m, 1H), 3.31 – 3.22 (m, 2H), 3.00 (s, 3H), 2.08 – 1.98 (m, 1H), 1.17 (t, *J* = 7.1 Hz, 3H).

^{13}C NMR (101 MHz, CDCl$_3$) δ 170.1, 169.9, 157.4, 148.3, 136.2, 128.4, 116.2, 105.8, 62.2, 59.3, 56.1, 46.9, 30.8, 30.7, 14.2.

HRMS (APCI) m/z: $[M+H]^+$ Calculated for $[C_{15}H_{19}O_6N_2]^+$ 323.1238; found 323.1236.

XRD Recrystalised from EtOAc/hexane as light yellow needles. Single crystal analysis confirms the structure drawn (CCDC deposition number: 2277260)

mp: 92-94 °C.

methyl 3-(2-methoxy-4-nitrophenyl)-1-methyl-2-oxopyrrolidine-3-carboxylate (3d)

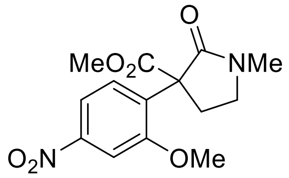

Prepared according to general procedure D (column conditions: 0-60% EtOAc in hexane). The pure product was afforded as a yellow solid (28 mg, 45% yield).

^{1}H NMR (400 MHz, $CDCl_3$) δ 7.83 (dd, *J* = 8.5, 2.3 Hz, 1H), 7.72 (d, *J* = 2.2 Hz, 1H), 7.49 (d, *J* = 8.5 Hz, 1H), 3.90 (s, 3H), 3.70 (s, 3H), 3.60 – 3.51 (m, 1H), 3.32 – 3.24 (m, 2H), 3.00 (s, 3H), 2.06 – 1.98 (m, 1H).

^{13}C NMR (101 MHz, $CDCl_3$) δ 170.7, 169.6, 157.5, 148.4, 136.2, 128.5, 116.3, 106.1, 59.2, 56.3, 53.3, 46.9, 30.9, 30.8.

HRMS (APCI) m/z: $[M+H]^+$ Calculated for $[C_{14}H_{17}O_6N_2]^+$ 309.1081; found 309.1069.

XRD Recrystalised from EtOAc/hexane as yellow crystals. Single crystal analysis confirms the structure drawn (CCDC deposition number: 2277255)

mp: 164-166 °C.

allyl 3-(2-methoxy-4-nitrophenyl)-1-methyl-2-oxopyrrolidine-3-carboxylate (3e)

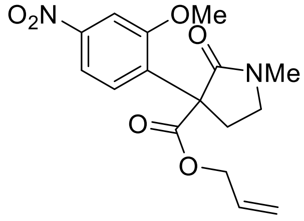

Prepared according to general procedure D (column conditions: 0-60% EtOAc in hexane). As product co-eluted with the sulfonamide starting material, the mixture was subjected to acetylation conditions (AcCl, Et_3N, DMAP, DCM, rt, 16h). This selectively acetylated the sulfonamide and allowed for isolation of the product as a yellow solid (15 mg, 22% yield) (40 % NMR yield).

^{1}H NMR (400 MHz, $CDCl_3$) δ 7.83 (dd, *J* = 8.4, 2.2 Hz, 1H), 7.71 (d, *J* = 2.2 Hz, 1H), 7.51 (d, *J* = 8.5 Hz, 1H), 5.87 – 5.73 (m, 1H), 5.25 – 5.14 (m, 2H), 4.73 – 4.50 (m, 2H), 3.88 (s, 3H), 3.59 – 3.51 (m, 1H), 3.32 – 3.22 (m, 2H), 3.00 (s, 3H), 2.09 – 1.98 (m, 1H).

^{13}C NMR (101 MHz, CDCl$_3$) δ 169.9, 169.6, 157.5, 148.4, 136.2, 131.8, 128.5, 118.7, 116.2, 105.8, 66.7, 59.3, 56.2, 46.9, 30.9, 30.7.

HRMS (ESI) m/z: [M+Na]$^+$ Calculated for $[C_{16}H_{18}O_6N_2Na]^+$ 357.1057; found 357.1065.

mp: 64-66 °C.

prop-2-yn-1-yl 3-(2-methoxy-4-nitrophenyl)-1-methyl-2-oxopyrrolidine-3-carboxylate (3f)

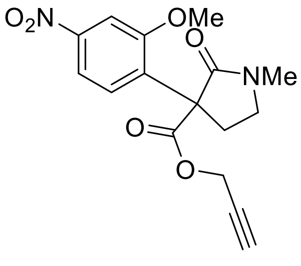

Prepared according to general procedure D (column conditions: 0-50% EtOAc in hexane). The pure product was afforded as a yellow solid (23 mg, 35% yield).

^{1}H NMR (400 MHz, CDCl$_3$) δ 7.83 (dd, *J* = 8.5, 2.2 Hz, 1H), 7.73 (d, *J* = 2.3 Hz, 1H), 7.48 (d, *J* = 8.5 Hz, 1H), 4.88 (dd, *J* = 15.5, 2.4 Hz, 1H), 4.53 (dd, *J* = 15.5, 2.5 Hz, 1H), 3.91 (s, 3H), 3.60 – 3.51 (m, 1H), 3.36 – 3.22 (m, 2H), 3.00 (s, 3H), 2.43 (t, *J* = 2.4 Hz, 1H), 2.10 – 2.00 (m, 1H).

^{13}C NMR (101 MHz, CDCl$_3$) δ 169.6, 169.3, 157.5, 148.5, 135.6, 128.4, 116.2, 105.9, 77.6, 74.8, 59.2, 56.3, 53.2, 46.8, 30.9, 30.8.

HRMS (ESI) m/z: [M+Na]$^+$ Calculated for $[C_{16}H_{16}O_6N_2Na]^+$ 355.0901; found 355.0901.

mp: 112-114 °C.

N-(4-fluorophenyl)-3-(2-methoxy-4-nitrophenyl)-1-methyl-2-oxopyrrolidine-3-carboxamide (3g)

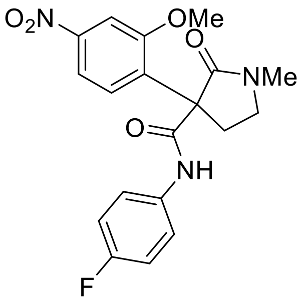

Prepared according to general procedure C (column conditions: 0-80% EtOAc in hexane). The pure product was afforded as a red solid (15 mg, 19% yield).

^{1}H NMR (500 MHz, CDCl$_3$) δ 8.47 (s, 1H), 7.83 – 7.80 (m, 1H), 7.75 (d, *J* = 2.3 Hz, 1H), 7.48 – 7.40 (m, 3H), 7.04 – 6.95 (m, 2H), 3.92 (s, 3H), 3.55 – 3.50 (m, 1H), 3.43 – 3.37 (m, 2H), 2.99 (s, 3H), 2.14 – 2.04 (m, 1H).

^{13}C NMR (126 MHz, CDCl$_3$) δ 172.6, 166.4, 159.6 (d, *J* = 244.1 Hz), 157.9, 148.6, 136.6, 133.8 (d, *J* = 2.7 Hz), 129.5, 121.7 (d, *J* = 7.9 Hz), 116.2, 115.8 (d, *J* = 22.6 Hz), 106.8, 60.3 (d, *J* = 2.0 Hz), 56.7, 47.4, 30.8, 28.3.

^{19}F NMR (471 MHz, CDCl$_3$) δ -117.71 – -117.79 (m).

HRMS (APCI) m/z: $[M+H]^+$ Calculated for $[C_{19}H_{19}O_5N_3F]^+$ 388.1303; found 388.1289.

mp: 168-170 °C.

ethyl 1-ethyl-3-(2-methoxy-4-nitrophenyl)-2-oxopyrrolidine-3-carboxylate (3h)

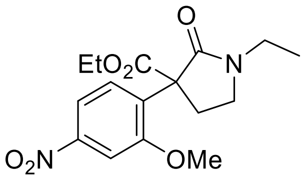

Prepared according to general procedure C (column conditions: 0-40% EtOAc in hexane). The pure product was afforded as a yellow solid (31 mg, 46% yield).

^{1}H NMR (400 MHz, CDCl$_3$) δ 7.86 – 7.80 (m, 1H), 7.71 (d, *J* = 2.3 Hz, 1H), 7.48 (d, *J* = 8.4 Hz, 1H), 4.31 – 4.20 (m, 1H), 4.16 – 4.05 (m, 1H), 3.89 (s, 3H), 3.57 – 3.37 (m, 3H), 3.29 – 3.21 (m, 2H), 2.10 – 1.97 (m, 1H), 1.22 – 1.15 (m, 6H).

^{13}C NMR (101 MHz, CDCl$_3$) δ 170.1, 169.4, 157.5, 148.3, 136.2, 128.4, 116.2, 105.8, 62.2, 59.7, 56.1, 44.0, 38.3, 30.8, 14.2, 12.5.

HRMS (APCI) m/z: $[M+H]^+$ Calculated for $[C_{16}H_{21}O_6N_2]^+$ 337.1394; found 337.1400.

mp: 78-80 °C.

ethyl 3-(2-methoxy-4-nitrophenyl)-2-oxo-1-propylpyrrolidine-3-carboxylate (3i)

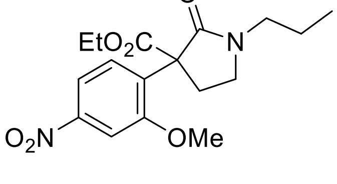

Prepared according to general procedure C (column conditions: 0-40% EtOAc in hexane). The pure product was afforded as an orange oil (27 mg, 39% yield) (70% NMR yield).

^{1}H NMR (400 MHz, $CDCl_3$) δ 7.83 (dd, *J* = 8.5, 2.2 Hz, 1H), 7.71 (d, *J* = 2.2 Hz, 1H), 7.51 (d, *J* = 8.5 Hz, 1H), 4.31 – 4.20 (m, 1H), 4.16 – 4.08 (m, 1H), 3.89 (s, 3H), 3.58 – 3.48 (m, 1H), 3.45 – 3.30 (m, 2H), 3.29 – 3.20 (m, 2H), 2.08 – 1.97 (m, 1H), 1.67 – 1.56 (m, 2H), 1.17 (t, *J* = 7.1 Hz, 3H), 0.94 (t, *J* = 7.4 Hz, 3H).

^{13}C NMR (101 MHz, $CDCl_3$) δ 170.2, 169.6, 157.5, 148.3, 136.4, 128.5, 116.3, 105.8, 62.1, 59.7, 56.1, 45.2, 44.6, 30.9, 20.6, 14.2, 11.4.

HRMS (APCI) m/z: $[M+H]^+$ Calculated for $[C_{17}H_{23}O_6N_2]^+$ 351.1551; found 351.1558.

ethyl 3-(2-methoxy-4-nitrophenyl)-1-(3-methoxypropyl)-2-oxopyrrolidine-3-carboxylate (3j)

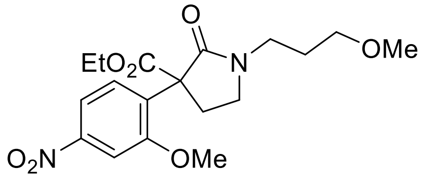

Prepared according to general procedure C (column conditions: 0-40% EtOAc in hexane). The pure product was afforded as an orange oil (26 mg, 34% yield) (51% NMR yield).

^{1}H NMR (400 MHz, $CDCl_3$) δ 7.83 (dd, *J* = 8.5, 2.2 Hz, 1H), 7.71 (d, *J* = 2.2 Hz, 1H), 7.50 (d, *J* = 8.5 Hz, 1H), 4.30 – 4.20 (m, 1H), 4.16 – 4.06 (m, 1H), 3.89 (s, 3H), 3.60 – 3.45 (m, 3H), 3.42 (t, *J* = 6.1 Hz, 2H), 3.33 (s, 3H), 3.29 – 3.20 (m, 2H), 2.08 – 1.98 (m, 1H), 1.93 – 1.81 (m, 2H), 1.17 (t, *J* = 7.1 Hz, 3H).

^{13}C NMR (101 MHz, $CDCl_3$) δ 170.2, 169.7, 157.5, 148.3, 136.3, 128.4, 116.2, 105.8, 70.1, 62.1, 59.6, 58.9, 56.1, 44.9, 41.1, 30.9, 27.6, 14.2.

HRMS (APCI) m/z: $[M+H]^+$ Calculated for $[C_{18}H_{25}O_7N_2]^+$ 381.1656; found 381.1657.

ethyl 1-cyclopropyl-3-(2-methoxy-4-nitrophenyl)-2-oxopyrrolidine-3-carboxylate (3k)

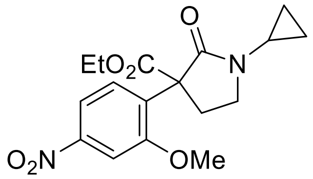

Prepared according to general procedure C (column conditions: 0-40% EtOAc in hexane). The pure product was afforded as a yellow solid (17 mg, 24% yield).

^{1}H NMR (400 MHz, $CDCl_3$) δ 7.83 (dd, *J* = 8.4, 2.2 Hz, 1H), 7.71 (d, *J* = 2.2 Hz, 1H), 7.47 (d, *J* = 8.5 Hz, 1H), 4.29 – 4.20 (m, 1H), 4.12 (dq, *J* = 10.7, 7.1 Hz, 1H), 3.88 (s, 3H), 3.49 – 3.42 (m, 1H), 3.25 – 3.09 (m, 2H), 2.87 – 2.80 (m, 1H), 2.03 – 1.95 (m, 1H), 1.17 (t, *J* = 7.1 Hz, 3H), 0.94 – 0.69 (m, 4H).

^{13}C NMR (101 MHz, $CDCl_3$) δ 170.8, 170.0, 157.4, 148.3, 136.2, 128.3, 116.2, 105.8, 62.1, 60.2, 56.1, 44.8, 30.7, 26.4, 14.2, 5.4, 5.1.

HRMS (ESI) m/z: $[M+Na]^+$ Calculated for $[C_{17}H_{20}O_6N_2Na]^+$ 371.1214; found 371.1213.

mp: 94-96 °C.

ethyl 1-allyl-3-(2-methoxy-4-nitrophenyl)-2-oxopyrrolidine-3-carboxylate (3l)

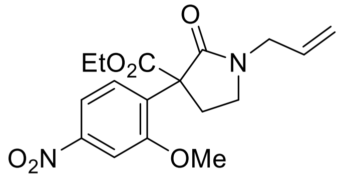

Prepared according to general procedure C (column conditions: 0-80% EtOAc in hexane). As product co-eluted with the sulfonamide starting material, the mixture was subjected to acetylation conditions (AcCl, Et_3N, DMAP, DCM, rt, 16h). This selectively acetylated the sulfonamide and allowed for isolation of the product as a yellow solid (21 mg, 30% yield).

^{1}H NMR (400 MHz, $CDCl_3$) δ 7.84 (dd, *J* = 8.5, 2.3 Hz, 1H), 7.72 (d, *J* = 2.3 Hz, 1H), 7.50 (d, *J* = 8.5 Hz, 1H), 5.83 – 5.70 (m, 1H), 5.29 – 5.19 (m, 2H), 4.31 – 4.22 (m, 1H), 4.18 – 3.94 (m, 3H), 3.90 (s, 3H), 3.55 – 3.47 (m, 1H), 3.30 – 3.17 (m, 2H), 2.09 – 1.98 (m, 1H), 1.18 (t, *J* = 7.1 Hz, 3H).

^{13}C NMR (101 MHz, $CDCl_3$) δ 170.1, 169.5, 157.5, 148.3, 136.2, 131.8, 128.4, 118.7, 116.3, 105.9, 62.2, 59.6, 56.1, 46.2, 44.3, 30.8, 14.2.

HRMS (APCI) m/z: $[M+H]^+$ Calculated for $[C_{17}H_{21}O_6N_2]^+$ 349.1394; found 349.1391.

mp: 64-66 °C.

ethyl 1-(2-(cyclohex-1-en-1-yl)ethyl)-3-(2-methoxy-4-nitrophenyl)-2-oxopyrrolidine-3-carboxylate (3m)

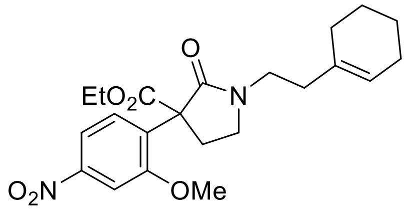

Prepared according to general procedure C (column conditions: 0-40% EtOAc in hexane). The pure product was afforded as a yellow oil (10 mg, 12% yield) (63% NMR yield).

^{1}H NMR (400 MHz, CDCl$_3$) δ 7.81 (dd, *J* = 8.5, 2.2 Hz, 1H), 7.71 (d, *J* = 2.3 Hz, 1H), 7.48 (d, *J* = 8.4 Hz, 1H), 5.43 (s, 1H), 4.31 – 4.20 (m, 1H), 4.16 – 4.04 (m, 1H), 3.89 (s, 3H), 3.73 – 3.62 (m, 1H), 3.52 – 3.44 (m, 1H), 3.43 – 3.31 (m, 1H), 3.29 – 3.19 (m, 2H), 2.21 (t, *J* = 7.2 Hz, 2H), 2.05 – 1.89 (m, 4H), 1.68 – 1.51 (m, 5H), 1.17 (t, *J* = 7.1 Hz, 3H).

^{13}C NMR (101 MHz, CDCl$_3$) δ 170.2, 169.4, 157.5, 148.3, 136.5, 134.4, 128.6, 123.9, 116.1, 105.8, 62.1, 59.6, 56.0, 44.5, 41.7, 35.9, 30.9, 27.9, 25.5, 23.0, 22.4, 14.2.

HRMS (APCI) m/z: [M+H]$^+$ Calculated for $[C_{22}H_{29}O_6N_2]^+$ 417.2020; found 417.2024.

ethyl 3-(2-methoxy-4-nitrophenyl)-2-oxo-1-phenethylpyrrolidine-3-carboxylate (3n)

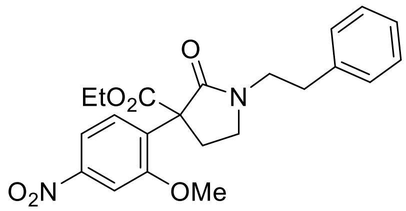

Prepared according to general procedure C (column conditions: 0-40% EtOAc in hexane). The pure product was afforded as a yellow oil (38 mg, 46% yield).

^{1}H NMR (400 MHz, CDCl$_3$) δ 7.67 (dq, *J* = 4.0, 2.3 Hz, 2H), 7.33 – 7.27 (m, 3H), 7.26 – 7.22 (m, 2H), 6.96 – 6.91 (m, 1H), 4.28 – 4.17 (m, 1H), 4.15 – 4.05 (m, 1H), 4.01 – 3.91 (m, 1H), 3.86 (s, 3H), 3.55 – 3.46 (m, 1H), 3.40 – 3.34 (m, 1H), 3.26 – 3.11 (m, 2H), 2.99 – 2.82 (m, 2H), 1.99 – 1.92 (m, 1H), 1.15 (t, *J* = 7.1 Hz, 3H).

^{13}C NMR (101 MHz, CDCl$_3$) δ 170.1, 169.7, 157.3, 148.2, 138.2, 136.1, 129.0, 128.8, 128.4, 126.8, 116.2, 105.6, 62.1, 59.5, 56.0, 44.7, 44.5, 33.7, 30.8, 14.2.

HRMS (APCI) m/z: [M+H]$^+$ Calculated for $[C_{22}H_{25}O_6N_2]^+$ 413.1707; found 413.1687.

ethyl 3-(2-methoxy-4-nitrophenyl)-1-(4-methoxyphenethyl)-2-oxopyrrolidine-3-carboxylate (3o)

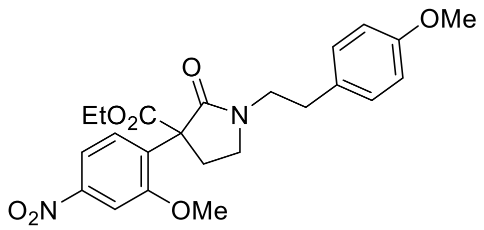

Prepared according to general procedure C (column conditions: 0-40% EtOAc in hexane). The pure product was afforded as a yellow solid (32 mg, 36% yield).

^{1}H NMR (400 MHz, CDCl$_3$) δ 7.68 – 7.62 (m, 2H), 7.18 – 7.13 (m, 2H), 6.89 – 6.81 (m, 3H), 4.28 – 4.18 (m, 1H), 4.13 – 4.03 (m, 1H), 4.00 – 3.90 (m, 1H), 3.86 (s, 3H), 3.82 (s, 3H), 3.49 – 3.39 (m, 1H), 3.40 – 3.32 (m, 1H), 3.25 – 3.11 (m, 2H), 2.97 – 2.75 (m, 2H), 1.99 – 1.91 (m, 1H), 1.15 (t, *J* = 7.1 Hz, 3H).

^{13}C NMR (101 MHz, CDCl$_3$) δ 170.1, 169.7, 158.6, 157.3, 148.2, 136.1, 130.1, 129.9, 128.4, 116.2, 114.2, 105.6, 62.1, 59.6, 56.0, 55.4, 44.6, 44.5, 32.8, 30.8, 14.2.

HRMS (ESI) m/z: $[M+Na]^+$ Calculated for $[C_{23}H_{26}O_7N_2Na]^+$ 465.1632; found 465.1634.

mp: 70-72 °C.

ethyl 3-(2-methoxy-4-nitrophenyl)-2-oxo-1-(4-(trifluoromethyl)phenethyl)pyrrolidine-3-carboxylate (3p)

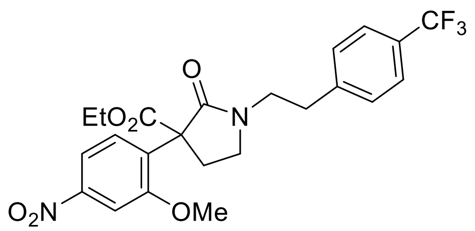

Prepared according to general procedure C (column conditions: 0-50% EtOAc in hexane). The pure product was afforded as a yellow solid (36 mg, 38% yield).

^{1}H NMR (500 MHz, CDCl$_3$) δ 7.74 – 7.70 (m, 1H), 7.68 (d, *J* = 2.2 Hz, 1H), 7.57 (d, *J* = 8.0 Hz, 2H), 7.37 (d, *J* = 7.9 Hz, 2H), 7.10 (d, *J* = 8.4 Hz, 1H), 4.26 – 4.17 (m, 1H), 4.11 – 4.03 (m, 1H), 3.87 (s, 3H), 3.86 – 3.80 (m, 1H), 3.67 – 3.61 (m, 1H), 3.46 – 3.39 (m, 1H), 3.25 – 3.14 (m, 2H), 2.98 (t, *J* = 7.7 Hz, 2H), 2.00 – 1.92 (m, 1H), 1.14 (t, *J* = 7.2 Hz, 3H).

^{13}C NMR (126 MHz, CDCl$_3$) δ 170.0, 169.7, 157.4, 148.3, 142.4, 136.0, 129.7 – 128.8 (m), 129.3, 128.2, 125.7 (q, *J* = 3.7 Hz), 127.7 – 120.8 (m), 116.1, 105.8, 62.2, 59.4, 56.0, 44.8, 44.3, 33.5, 30.9, 14.1.

^{19}F NMR (471 MHz, CDCl$_3$) δ -62.36.

HRMS (APCI) m/z: $[M+H]^+$ Calculated for $[C_{23}H_{24}O_6N_2F_3]^+$ 481.1581; found 481.1565.

mp: 84-86 °C.

ethyl 1-(4-bromophenethyl)-3-(2-methoxy-4-nitrophenyl)-2-oxopyrrolidine-3-carboxylate (3q)

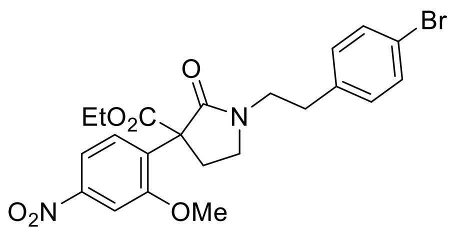

Prepared according to general procedure C (column conditions: 0-40% EtOAc in hexane). As product co-eluted with the sulfonamide starting material, the mixture was subjected to acetylation conditions (AcCl, Et_3N, DMAP, DCM, rt, 16h). This selectively acetylated the sulfonamide and allowed for isolation of the product as a yellow solid (30 mg, 31% yield).

^{1}H NMR (500 MHz, CDCl$_3$) δ 7.76 (dd, *J* = 8.4, 2.2 Hz, 1H), 7.68 (d, *J* = 2.2 Hz, 1H), 7.45 – 7.41 (m, 2H), 7.15 – 7.09 (m, 2H), 6.96 (d, *J* = 8.5 Hz, 1H), 4.26 – 4.18 (m, 1H), 4.14 – 4.04 (m, 1H), 3.92 – 3.87 (m, 1H), 3.86 (s, 4H), 3.56 – 3.49 (m, 1H), 3.42 – 3.36 (m, 1H), 3.25 – 3.12 (m, 2H), 2.94 – 2.81 (m, 2H), 2.00 – 1.91 (m, 1H), 1.15 (t, *J* = 7.1 Hz, 3H).

^{13}C NMR (126 MHz, CDCl$_3$) δ 170.0, 169.7, 157.3, 148.3, 137.2, 136.0, 131.9, 130.7, 128.3, 120.7, 116.2, 105.7, 62.2, 59.4, 56.0, 44.7, 44.2, 33.1, 30.8, 14.2.

HRMS (ESI) m/z: $[M+Na]^+$ Calculated for $[C_{22}H_{23}O_6N_2BrNa]^+$ 513.0632; found 513.0638.

mp: 98-100 °C.

ethyl 1-(3-chlorophenethyl)-3-(2-methoxy-4-nitrophenyl)-2-oxopyrrolidine-3-carboxylate (3r)

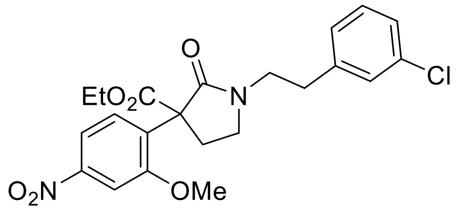

Prepared according to general procedure C (column conditions: 0-40% EtOAc in hexane). The pure product was afforded as a yellow solid (58 mg, 65% yield).

^{1}H NMR (500 MHz, CDCl$_3$) δ 7.74 (d, *J* = 8.4 Hz, 1H), 7.70 (s, 1H), 7.28 – 7.21 (m, 3H), 7.20 – 7.12 (m, 1H), 7.09 (d, *J* = 8.5 Hz, 1H), 4.29 – 4.20 (m, 1H), 4.15 – 4.07 (m, 1H), 3.92 – 3.90 (m, 1H), 3.88 (s, 3H), 3.59 – 3.53 (m, 1H), 3.45 – 3.38 (m, 1H), 3.27 – 3.15 (m, 2H), 2.97 – 2.85 (m, 2H), 2.03 – 1.96 (m, 1H), 1.17 (t, *J* = 7.1 Hz, 3H).

^{13}C NMR (126 MHz, CDCl$_3$) δ 170.0, 169.8, 157.3, 148.3, 140.3, 136.0, 134.5, 130.1, 129.1, 128.3, 127.1, 127.0, 116.2, 105.7, 62.2, 59.5, 56.0, 44.9, 44.3, 33.3, 30.9, 14.1.

HRMS (ESI) m/z: $[M+Na]^+$ Calculated for $[C_{22}H_{23}O_6N_2ClNa]^+$ 469.1137; found 469.1143.

mp: 104-106 °C.

ethyl 3-(2-methoxy-4-nitrophenyl)-2-oxo-1-(2-(pyridin-2-yl)ethyl)pyrrolidine-3-carboxylate (3s)

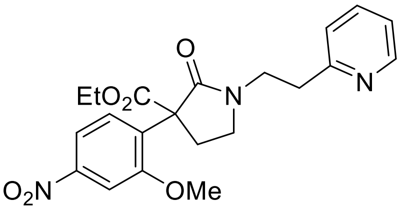

Prepared according to general procedure C (no HCl wash, column conditions: 0-100% EtOAc in hexane). The pure product was afforded as a yellow gum (41 mg, 50% yield).

^{1}H NMR (400 MHz, $CDCl_3$) δ 8.51 (d, *J* = 4.9 Hz, 1H), 7.72 (dd, *J* = 8.4, 2.3 Hz, 1H), 7.70 – 7.63 (m, 2H), 7.30 – 7.27 (m, 1H), 7.23 – 7.18 (m, 1H), 7.14 (d, *J* = 8.4 Hz, 1H), 4.28 – 4.18 (m, 1H), 4.13 – 4.05 (m, 1H), 4.03 – 3.93 (m, 1H), 3.86 (s, 3H), 3.76 – 3.67 (m, 1H), 3.46 – 3.38 (m, 1H), 3.27 – 3.16 (m, 2H), 3.12 (t, *J* = 7.0 Hz, 2H), 2.01 – 1.93 (m, 1H), 1.15 (t, *J* = 7.1 Hz, 3H).

^{13}C NMR (101 MHz, $CDCl_3$) δ 170.1, 169.8, 158.4, 157.4, 149.3, 148.2, 137.0, 136.1, 128.3, 123.7, 122.0, 116.1, 105.7, 62.1, 59.5, 56.0, 44.9, 43.4, 35.8, 30.9, 14.2.

HRMS (APCI) m/z: $[M+H]^+$ Calculated for $[C_{21}H_{24}O_6N_3]^+$ 414.1660; found 414.1644.

ethyl 3-(2-methoxy-4-nitrophenyl)-2-oxo-1-(2-(thiophen-2-yl)ethyl)pyrrolidine-3-carboxylate (3t)

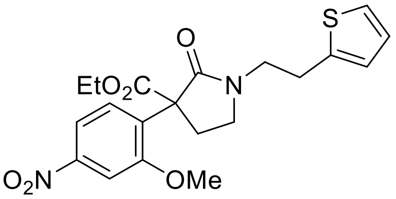

Prepared according to general procedure C (column conditions: 0-50% EtOAc in hexane). The pure product was afforded as a brown solid (50 mg, 60% yield).

^{1}H NMR (400 MHz, $CDCl_3$) δ 7.73 (dd, *J* = 8.5, 2.2 Hz, 1H), 7.68 (d, *J* = 2.3 Hz, 1H), 7.19 (dd, *J* = 5.1, 1.2 Hz, 1H), 7.10 (d, *J* = 8.5 Hz, 1H), 6.96 (dd, *J* = 5.1, 3.4 Hz, 1H), 6.92 – 6.87 (m, 1H), 4.29 – 4.18 (m, 1H), 4.15 – 4.06 (m, 1H), 3.95 (dt, *J* = 13.6, 7.5 Hz, 1H), 3.87 (s, 3H), 3.57 – 3.49 (m, 1H), 3.43 – 3.37 (m, 1H), 3.28 – 3.12 (m, 4H), 2.04 – 1.94 (m, 1H), 1.16 (t, *J* = 7.1 Hz, 3H).

^{13}C NMR (101 MHz, $CDCl_3$) δ 170.0, 169.8, 157.3, 148.2, 140.5, 136.1, 128.5, 127.3, 125.8, 124.2, 116.2, 105.7, 62.2, 59.5, 56.0, 44.9, 44.9, 30.9, 27.8, 14.2.

HRMS (APCI) m/z: $[M+H]^+$ Calculated for $[C_{20}H_{23}O_6N_2S]^+$ 419.1271; found 419.1257.

mp: 70-72 °C.

3-(2-methoxy-4-nitrophenyl)-1-methylpyrrolidin-2-one (4a)

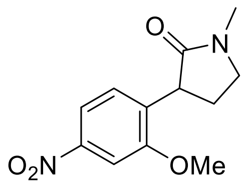

Prepared by adaptation of a literature procedure.[4] a 2-5 mL oven-dried microwave vial was charged with pyrrolidinone **3c** (0.168 mmol, 54 mg). The vial was sealed and EtOH (1 mL) was added, followed by 1M aqueous NaOH (0.67 mL). The mixture was heated in a heating mantle to 110 °C and stirred for 20 minutes. The reaction was then cooled, unsealed and diluted (EtOAc). The organic phase was washed (1M HCl, 10% LiCl), dried ($MgSO_4$) and concentrated *in vacuo*. Another 2-5 mL microwave vial was charged with the crude product. The vial was sealed and toluene (0.8 mL) was added, followed by 0.5M HCl (1 mL). The mixture was heated in a heating mantle to 110 °C and stirred for 2 hours. The reaction was then cooled, unsealed and diluted (DCM). The organic phase was washed with H_2O, dried with $MgSO_4$, and concentrated *in vacuo*. The crude product was then purified by flash column chromatography (0-50% EtOAc in hexane), affording the pure product as a yellow solid (23 mg, 55% yield).

^{1}H NMR (400 MHz, $CDCl_3$) δ 7.81 (dd, J = 8.4, 2.2 Hz, 1H), 7.71 (d, J = 2.2 Hz, 1H), 7.30 (d, J = 8.4 Hz, 1H), 4.01 (t, J = 9.3 Hz, 1H), 3.91 (s, 3H), 3.49 – 3.42 (m, 2H), 2.96 (d, J = 0.8 Hz, 3H), 2.57 – 2.46 (m, 1H), 2.05 – 1.91 (m, 1H).

^{13}C NMR (101 MHz, $CDCl_3$) δ 174.2, 157.9, 148.1, 136.4, 129.9, 116.3, 105.8, 56.3, 48.0, 43.7, 30.3, 26.7.

HRMS (ESI) m/z: $[M+Na]^+$ Calculated for $[C_{12}H_{14}O_4N_2Na]^+$ 273.0846, found 273.0844.

mp: 114-116 °C.

ethyl 3-(4-amino-2-methoxyphenyl)-1-methyl-2-oxopyrrolidine-3-carboxylate (4b)

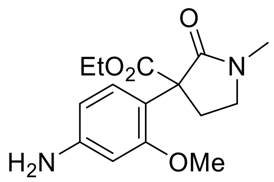

Prepared according to general procedure E (0.1 mmol scale, column conditions: 0-80% EtOAc in hexane). The pure product was afforded as a yellow oil (13 mg, 45% yield).

^{1}H NMR (400 MHz, CDCl$_3$) δ 6.95 (d, *J* = 8.1 Hz, 1H), 6.32 (d, *J* = 2.1 Hz, 1H), 6.28 (dd, *J* = 8.3, 2.1 Hz, 1H), 4.31 – 4.21 (m, 1H), 4.16 – 4.06 (m, 1H), 3.72 (s, 3H), 3.47 – 3.38 (m, 1H), 3.27 – 3.12 (m, 2H), 2.96 (s, 3H), 2.03 (ddd, *J* = 12.3, 8.6, 4.6 Hz, 1H), 1.19 (t, *J* = 7.1 Hz, 3H).

^{13}C NMR (101 MHz, CDCl$_3$) δ 171.7, 171.3, 157.9, 145.3, 128.4, 120.2, 107.9, 99.9, 61.7, 59.1, 55.3, 46.9, 31.5, 30.6, 14.3.

HRMS (ESI) m/z: $[M+Na]^+$ Calculated for $[C_{15}H_{20}O_4N_2Na]^+$ 315.1315; found 315.1316.

ethyl 3-(3,4'-dimethoxy-[1,1'-biphenyl]-4-yl)-1-methyl-2-oxopyrrolidine-3-carboxylate (4c)

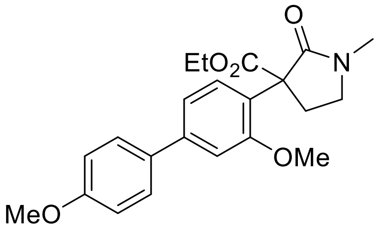

Prepared by adaptation of a literature procedure.[5] a 2-5 mL microwave vial was charged with pyrrolidinone **3c** (0.3 mmol, 96 mg), 4-anisyl boronic acid (0.45 mmol, 68 mg), palladium(II) acetylacetonate (5 mol%, 5 mg), Brettphos (20 mol%, 32 mg), 18-crown-6 (10 mol%, 8 mg) and K_3PO_4 (0.9 mmol, 191 mg). The vial was sealed, evacuated and backfilled under nitrogen. 1,4-dioxane (1.5 mL) was added, and the mixture was heated in a heating mantle to 130 °C, and stirred overnight. The reaction was then cooled, unsealed and diluted with DCM. The mixture was filtered over celite, dried with $MgSO_4$, and concentrated *in vacuo*. The crude product was then purified by flash column chromatography (0-90% EtOAc in hexane), affording the pure product as a yellow gum (58 mg, 50% yield).

^{1}H NMR (400 MHz, CDCl$_3$) δ 7.53 – 7.48 (m, 2H), 7.24 (d, *J* = 7.9 Hz, 1H), 7.10 (dd, *J* = 8.0, 1.8 Hz, 1H), 7.02 (d, *J* = 1.7 Hz, 1H), 6.99 – 6.94 (m, 2H), 4.35 – 4.24 (m, 1H), 4.18 – 4.08 (m, 1H), 3.85 (s, 3H), 3.84 (s, 3H), 3.51 – 3.43 (m, 1H), 3.30 – 3.21 (m, 2H), 2.99 (s, 3H), 2.16 – 2.04 (m, 1H), 1.20 (t, *J* = 7.1 Hz, 3H).

^{13}C NMR (101 MHz, CDCl$_3$) δ 171.3, 170.9, 159.4, 157.1, 141.7, 133.6, 128.3, 128.0, 127.4, 119.3, 114.3, 109.5, 61.8, 59.3, 55.5, 55.4, 46.9, 31.3, 30.6, 14.2.

HRMS (ESI) m/z: $[M+Na]^+$ Calculated for $[C_{22}H_{25}O_5NNa]^+$ 406.1625; found 406.1633.

ethyl 3-(2-methoxyphenyl)-1-methyl-2-oxopyrrolidine-3-carboxylate (4d)

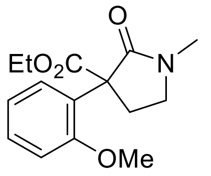

Prepared by adaptation of a literature procedure.[6] a 2-5 mL microwave vial was charged with pyrrolidinone **3c** (0.2 mmol, 64 mg), palladium(II) acetylacetonate (5 mol%, 3 mg), Brettphos (10 mol%, 11 mg), and K_3PO_4 (0.5 mmol, 106 mg).). The vial was sealed, evacuated and backfilled under nitrogen. 1,4-dioxane (1.5 mL) was added, followed by isopropanol (0.3 mmol, 18 mg). The mixture was heated in a heating mantle to 130 °C, and stirred overnight. The reaction was then cooled, unsealed and diluted with EtOAc. The mixture was filtered over a silica plug, dried with $MgSO_4$, and concentrated *in vacuo*. The crude product was then purified by flash column chromatography (0-80% EtOAc in hexane), affording the pure product as a yellow solid (40 mg, 72% yield).

^{1}H NMR (400 MHz, CDCl$_3$) δ 7.26 (t, *J* = 8.2 Hz, 1H), 7.20 (dd, *J* = 7.6, 1.7 Hz, 1H), 6.92 (t, *J* = 7.6 Hz, 1H), 6.87 (d, *J* = 8.2 Hz, 1H), 4.33 – 4.23 (m, 1H), 4.16 – 4.05 (m, 1H), 3.78 (s, 3H), 3.50 – 3.41 (m, 1H), 3.28 – 3.18 (m, 2H), 2.98 (s, 3H), 2.10 – 2.02 (m, 1H), 1.18 (t, *J* = 7.1 Hz, 3H).

^{13}C NMR (101 MHz, CDCl$_3$) δ 171.3, 170.9, 156.9, 128.9, 128.7, 127.7, 120.9, 110.9, 61.8, 59.5, 55.3, 46.8, 31.2, 30.6, 14.2.

HRMS (ESI) m/z: $[M+Na]^+$ Calculated for $[C_{15}H_{19}O_4NNa]^+$ 300.1206; found 300.1213.

mp: 104-106 °C.

1'-methylspiro[indoline-3,3'-pyrrolidine]-2,2'-dione (4e)

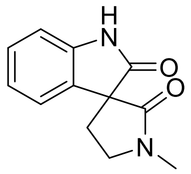

Prepared according to general procedure E (0.8 mmol scale, column conditions: 0-40% EtOAc in hexane). The pure product was afforded as an off-white solid (110 mg, 64% yield).

^{1}H NMR (400 MHz, CDCl$_3$) δ 7.99 (s, 1H), 7.21 (td, *J* = 7.7, 1.3 Hz, 1H), 7.15 – 7.10 (m, 1H), 7.03 (td, *J* = 7.6, 1.1 Hz, 1H), 6.85 (dt, *J* = 7.9, 0.8 Hz, 1H), 3.82 – 3.71 (m, 1H), 3.66 – 3.54 (m, 1H), 2.99 (s, 3H), 2.78 – 2.67 (m, 1H), 2.46 – 2.34 (m, 1H).

^{13}C NMR (101 MHz, CDCl$_3$) δ 177.4, 170.6, 141.5, 130.5, 129.1, 123.3, 123.1, 110.3, 58.3, 47.4, 30.7, 29.5.

HRMS (ESI) m/z: $[M+Na]^+$ Calculated for $[C_{12}H_{12}O_2N_2Na]^+$ 239.0791; found 239.0790.

Data are in accordance with literature.[7]

diethyl 2-(2-((N,4-dimethylphenyl)sulfonamido)ethyl)malonate (5a)

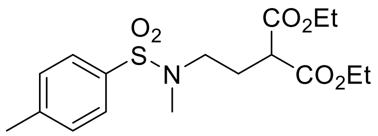

Prepared according to general procedure C (column conditions: 0-40% EtOAc in hexane). The pure product was afforded as a yellow oil (18 mg, 24% yield).

^{1}H NMR (400 MHz, $CDCl_3$) δ 7.66 – 7.61 (m, 2H), 7.31 (d, *J* = 8.0 Hz, 2H), 4.22 (q, *J* = 7.2 Hz, 4H), 3.54 (t, *J* = 7.1 Hz, 1H), 3.05 (t, *J* = 6.6 Hz, 2H), 2.69 (s, 3H), 2.42 (s, 3H), 2.14 (q, *J* = 6.8 Hz, 2H), 1.29 (t, *J* = 7.1 Hz, 6H).

^{13}C NMR (101 MHz, $CDCl_3$) δ 169.3, 143.6, 134.4, 129.8, 127.6, 61.8, 49.1, 48.3, 35.0, 26.8, 21.6, 14.2.

HRMS (ESI) m/z: $[M+Na]^+$ Calculated for $[C_{17}H_{25}O_6NSNa]^+$ 394.1295; found 394.1296.

6. NMR Spectra

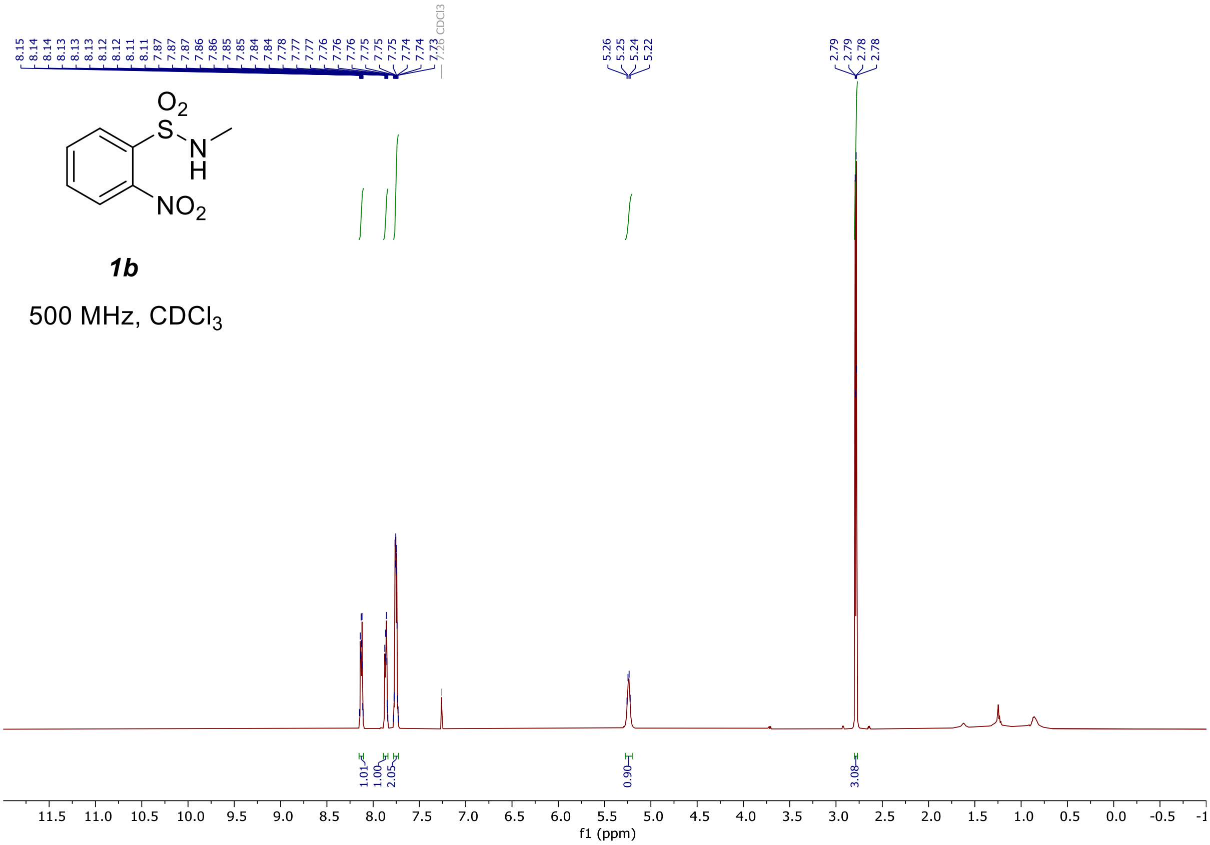

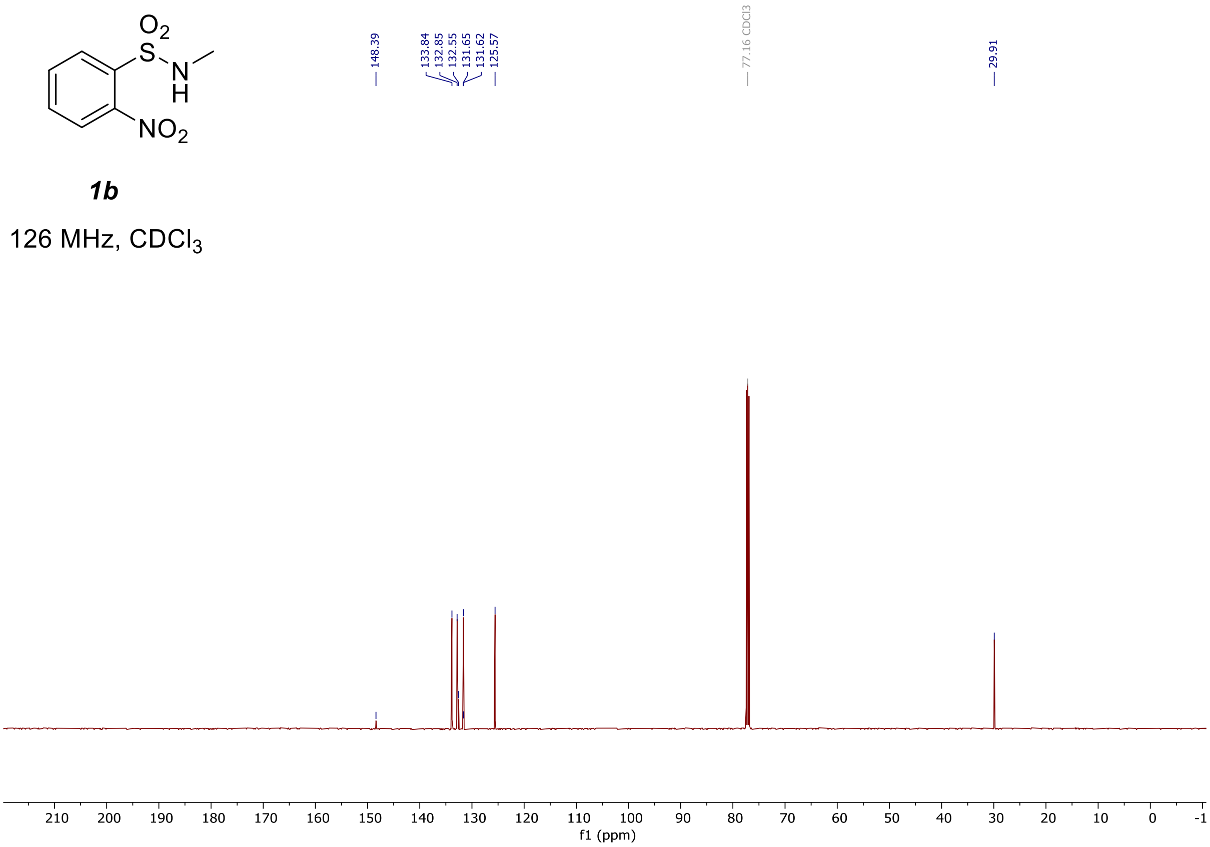

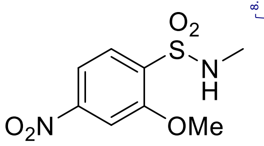

1c

500 MHz, $CDCl_3$

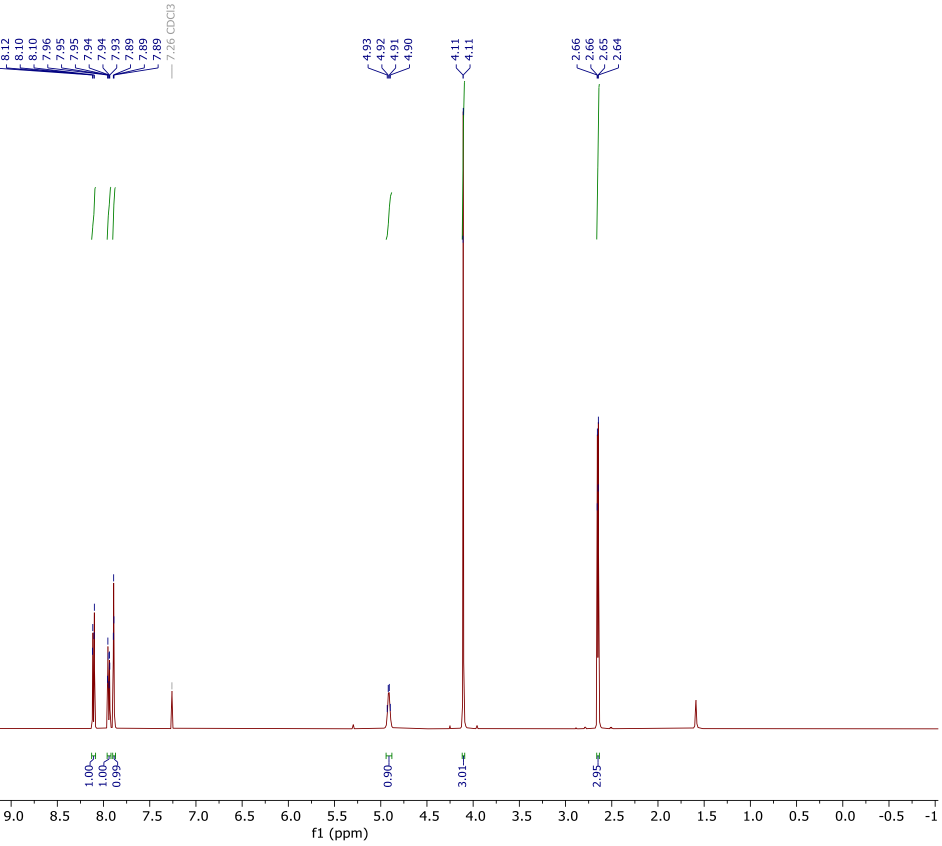

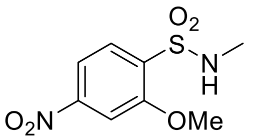

1c

126 MHz, $CDCl_3$

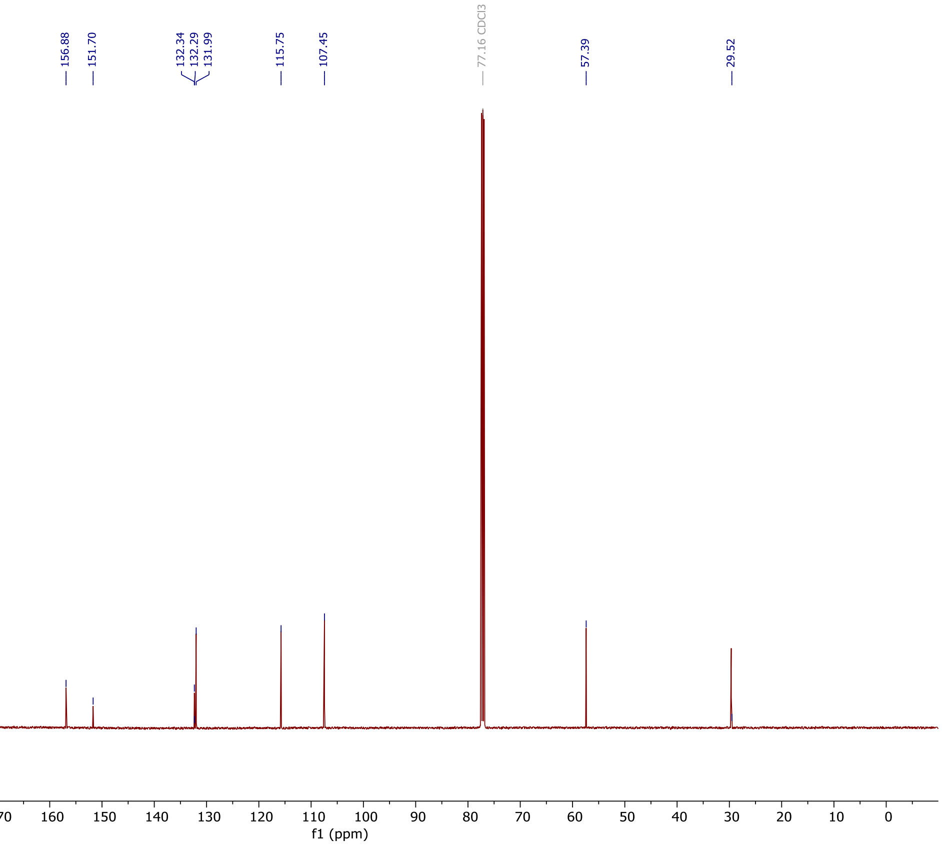

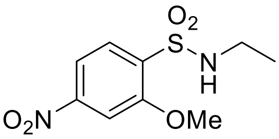

1h

500 MHz, $CDCl_3$

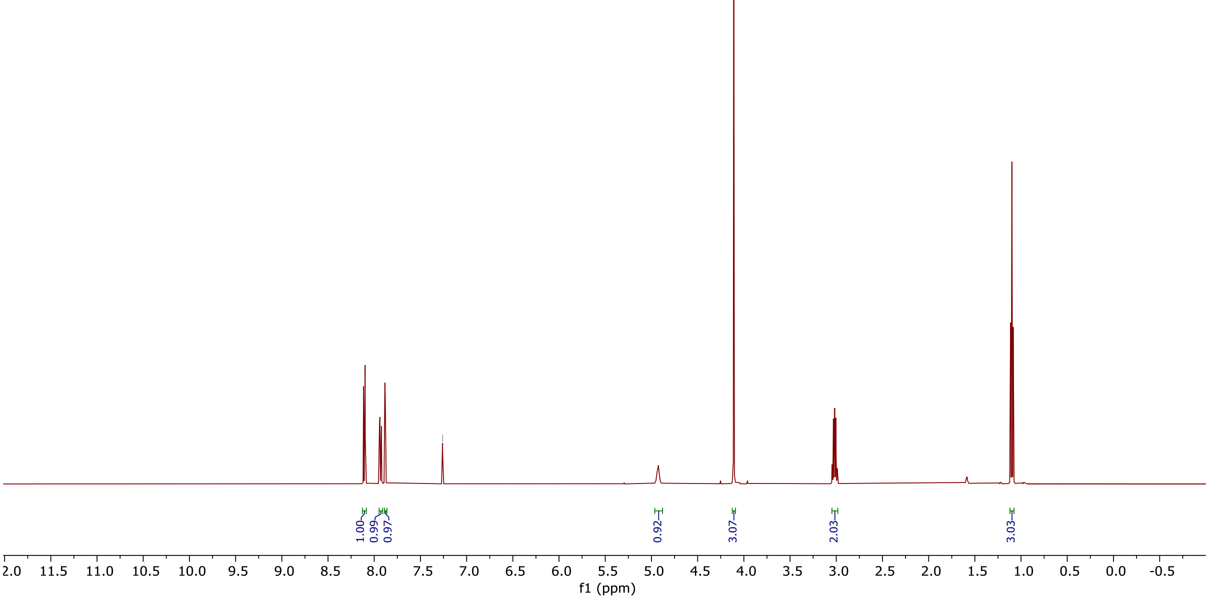

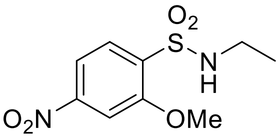

1h

126 MHz, $CDCl_3$

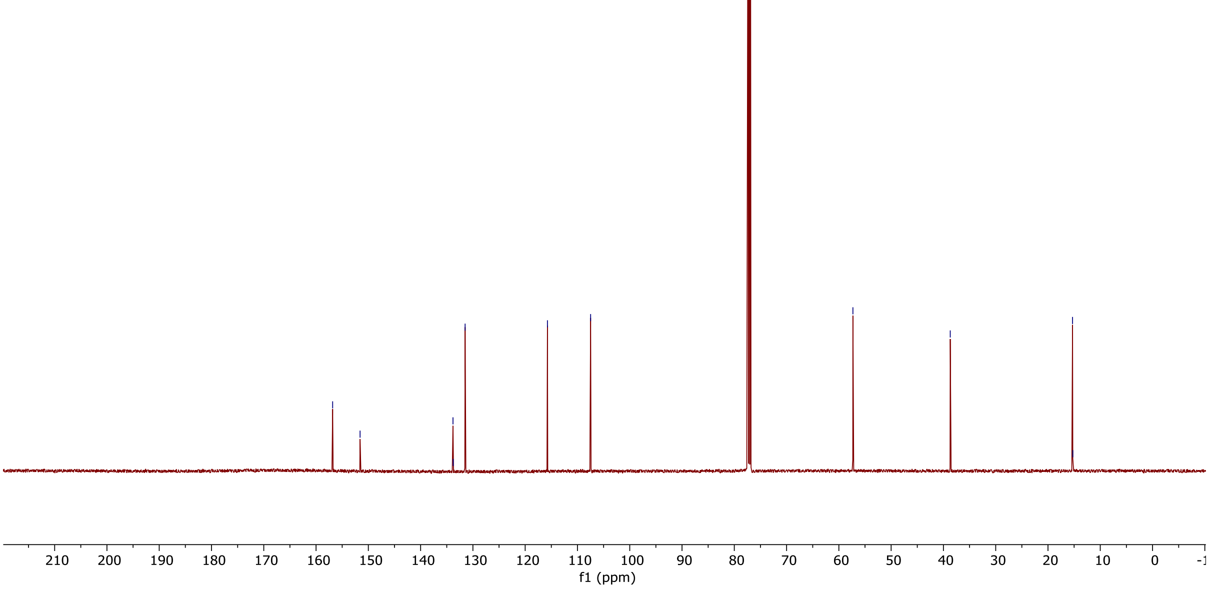

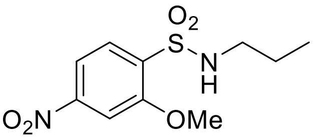

1i
500 MHz, $CDCl_3$

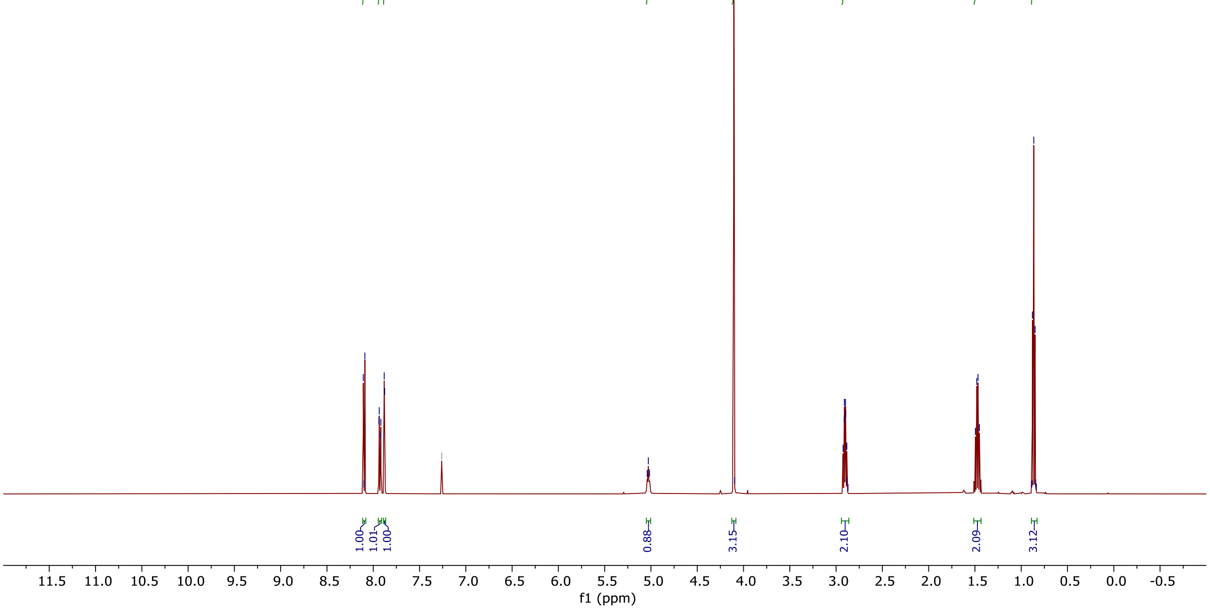

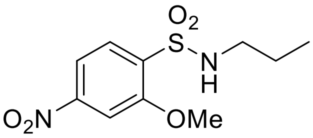

1i
126 MHz, $CDCl_3$

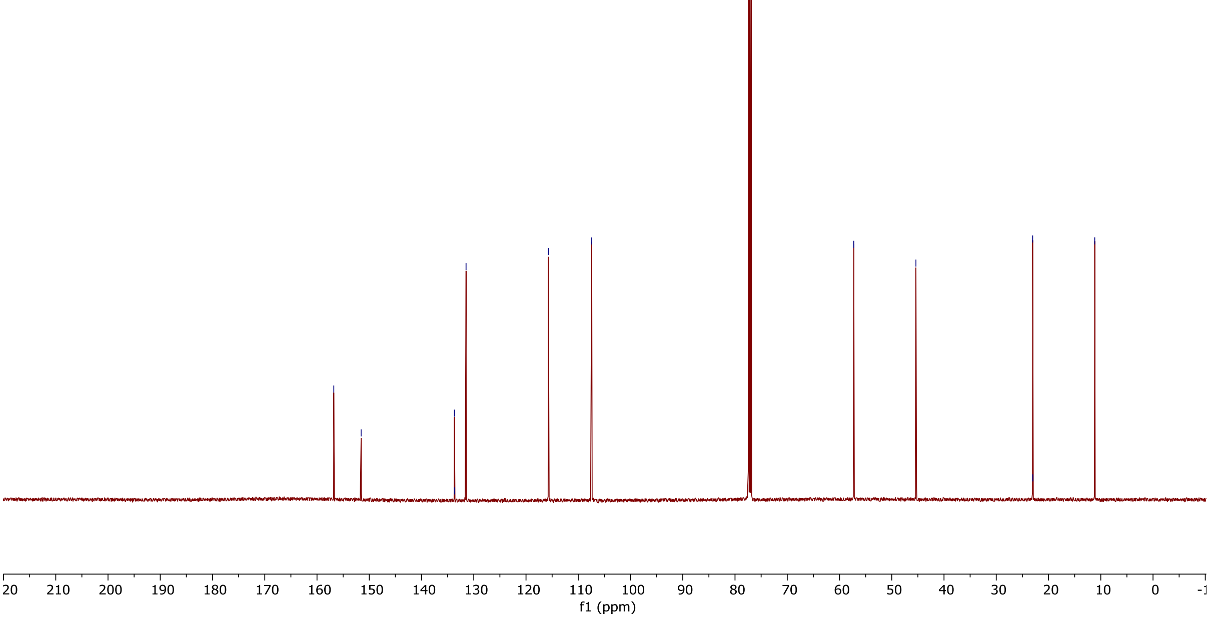

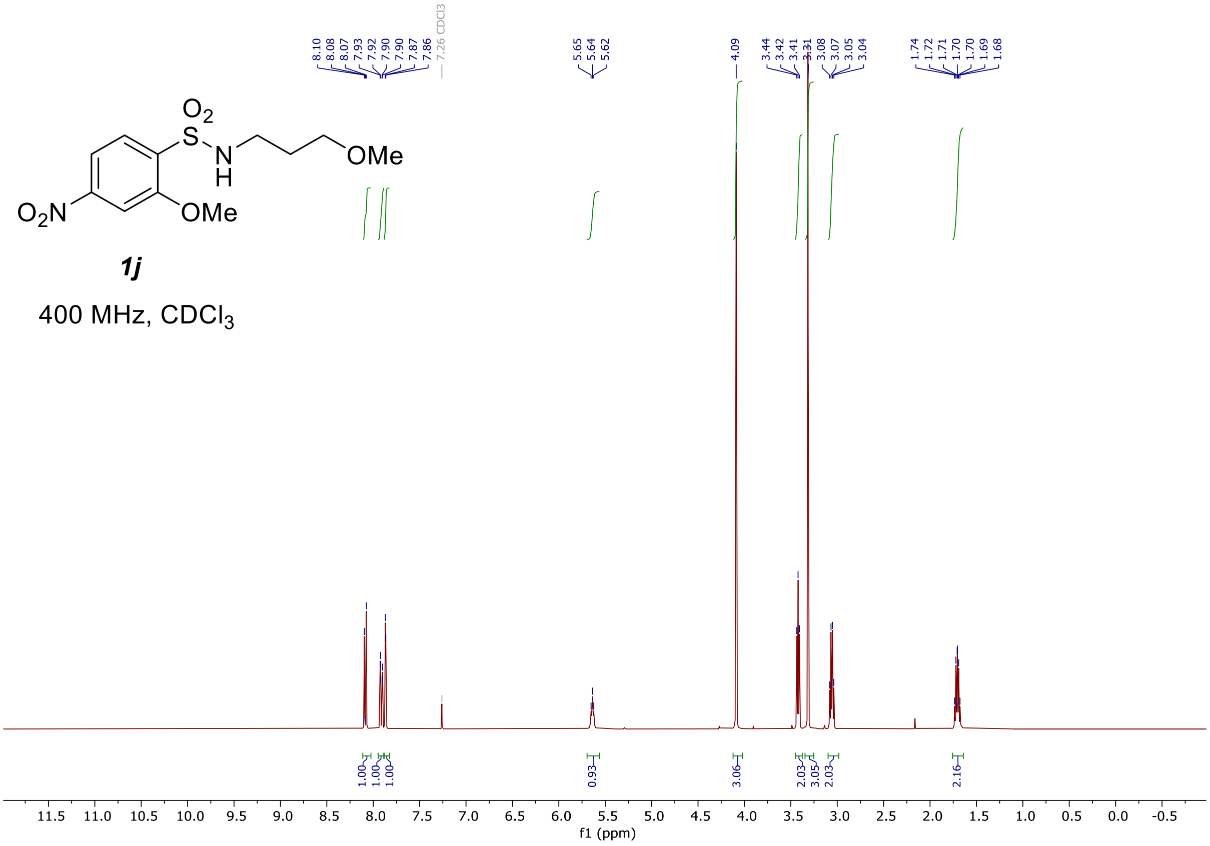
1j
400 MHz, CDCl3
8.10 8.08 8.07 7.93 7.92 7.90 7.90 7.87 7.86
7.26 CDCl3
5.65 5.64 5.62
4.09
3.44 3.42 3.41 3.31 3.08 3.07 3.05 3.04
1.74 1.72 1.71 1.70 1.69 1.68
1.00 1.00 1.00 0.93 3.06 2.03 3.05 2.03 2.16
f1 (ppm)

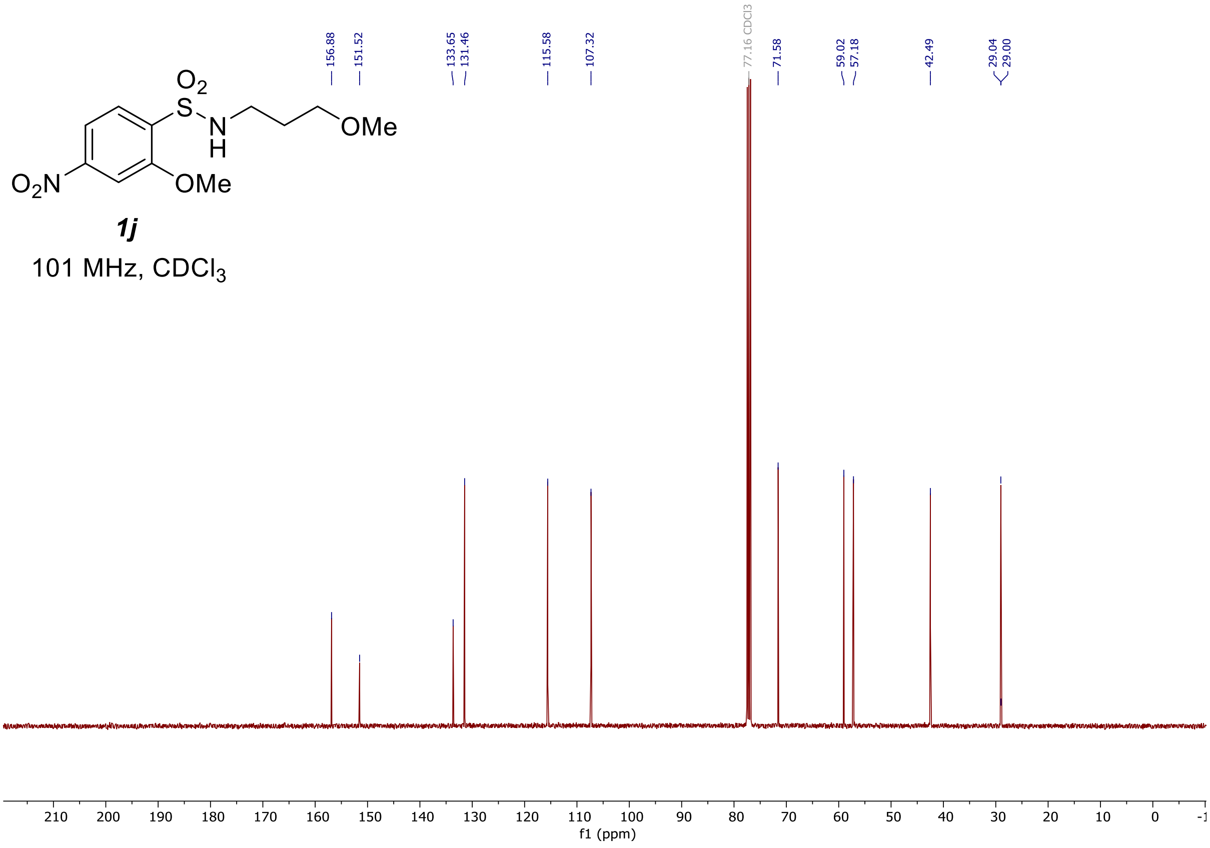
1j
101 MHz, CDCl3
156.88 151.52 133.65 131.46 115.58 107.32
77.16 CDCl3
71.58 59.02 57.18 42.49 29.04 29.00
f1 (ppm)

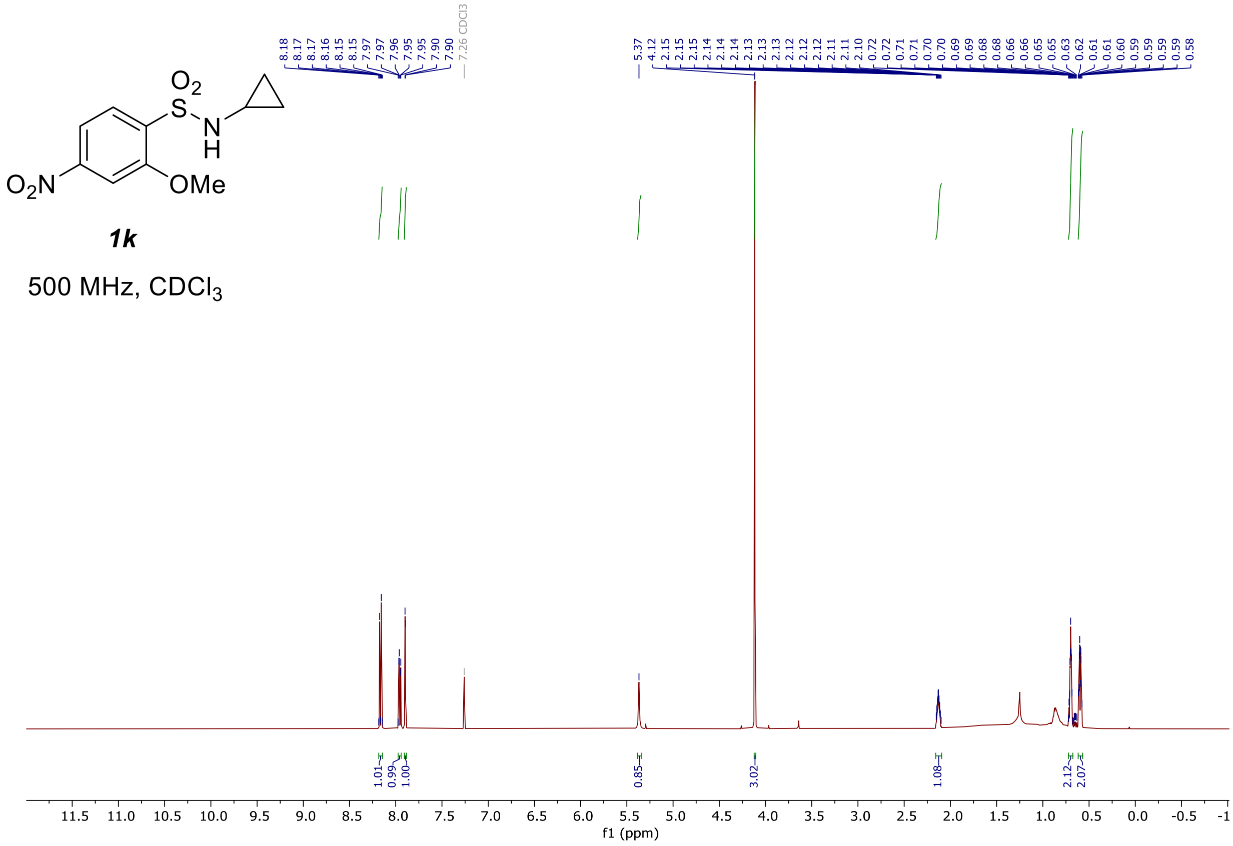

1k
500 MHz, CDCl3
f1 (ppm)

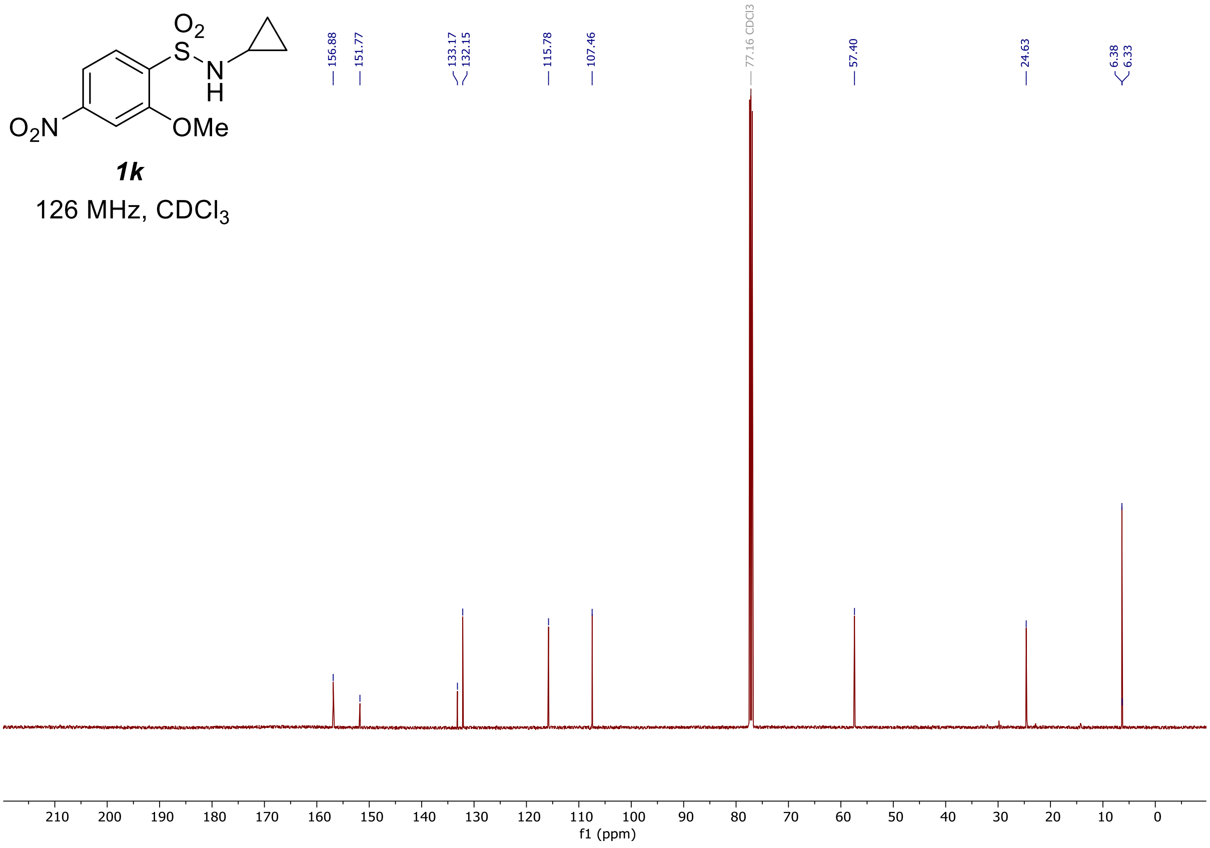

1k
126 MHz, CDCl3
156.88
151.77
133.17
132.15
115.78
107.46
77.16 CDCl3
57.40
24.63
6.38
6.33
f1 (ppm)

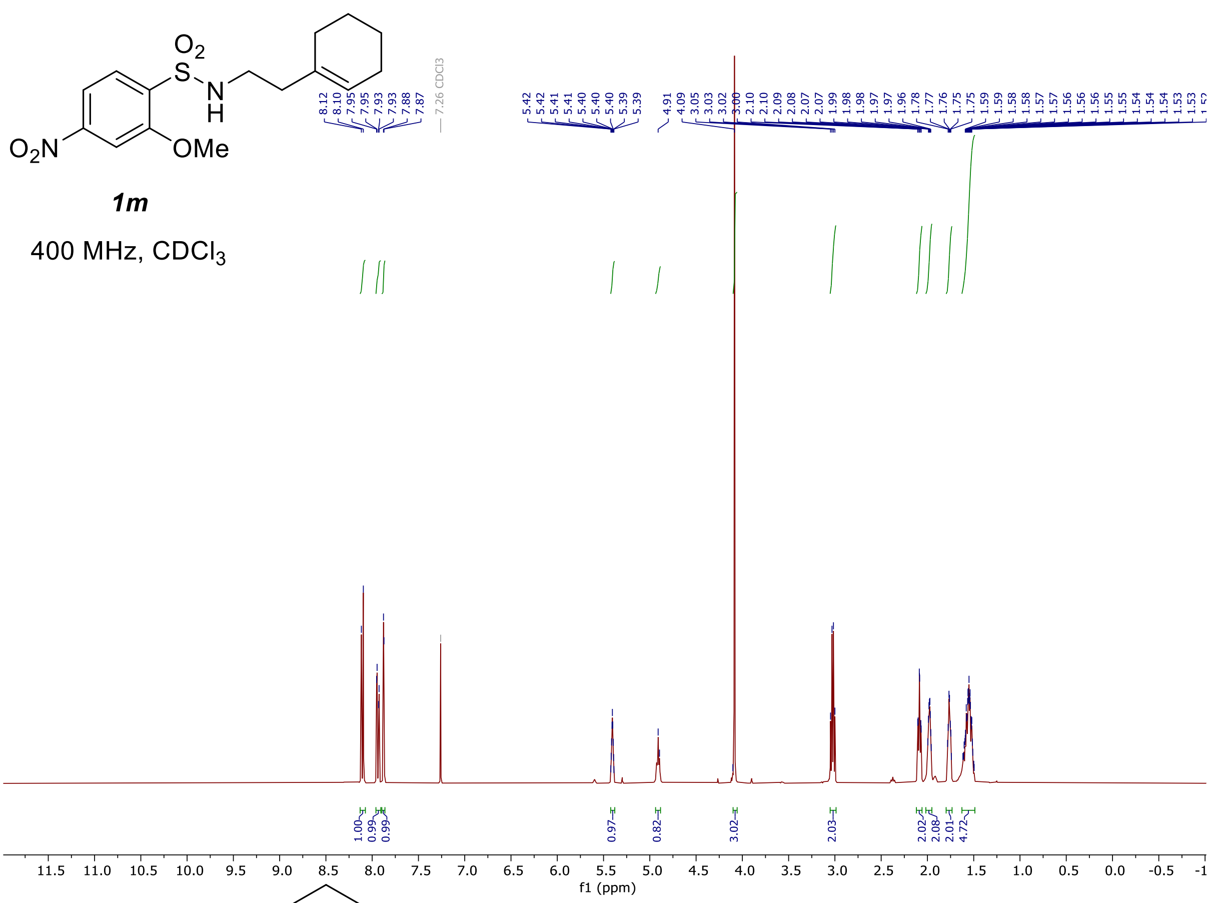
O2
S
N
H
O2N
OMe
1m
400 MHz, CDCl3
f1 (ppm)

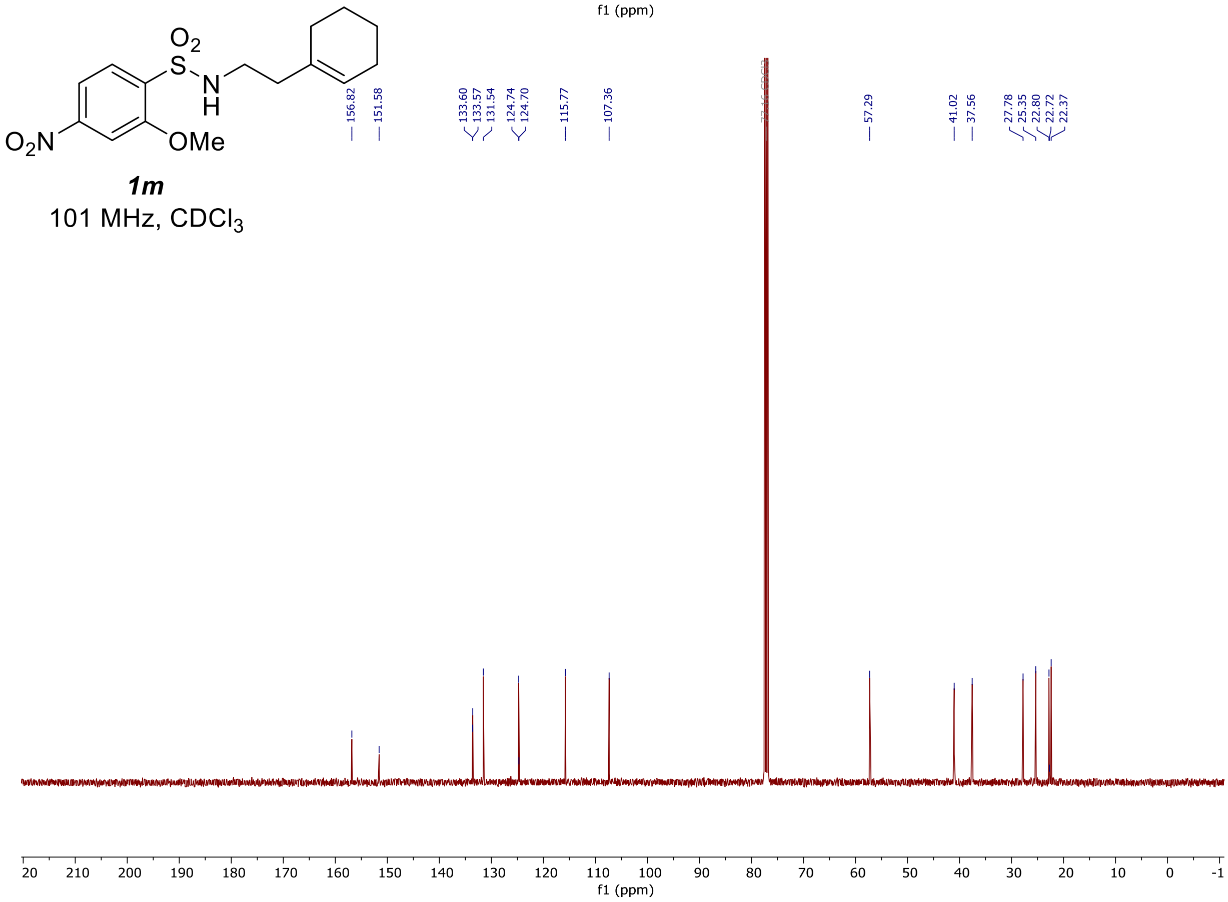
O2
S
N
H
O2N
OMe
1m
101 MHz, CDCl3
156.82
151.58
133.60
133.57
131.54
124.74
124.70
115.77
107.36
57.29
41.02
37.56
27.78
25.35
22.80
22.72
22.37
f1 (ppm)

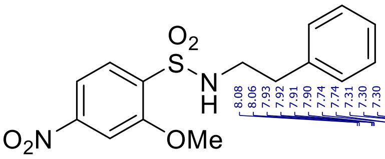

1n

400 MHz, $CDCl_3$

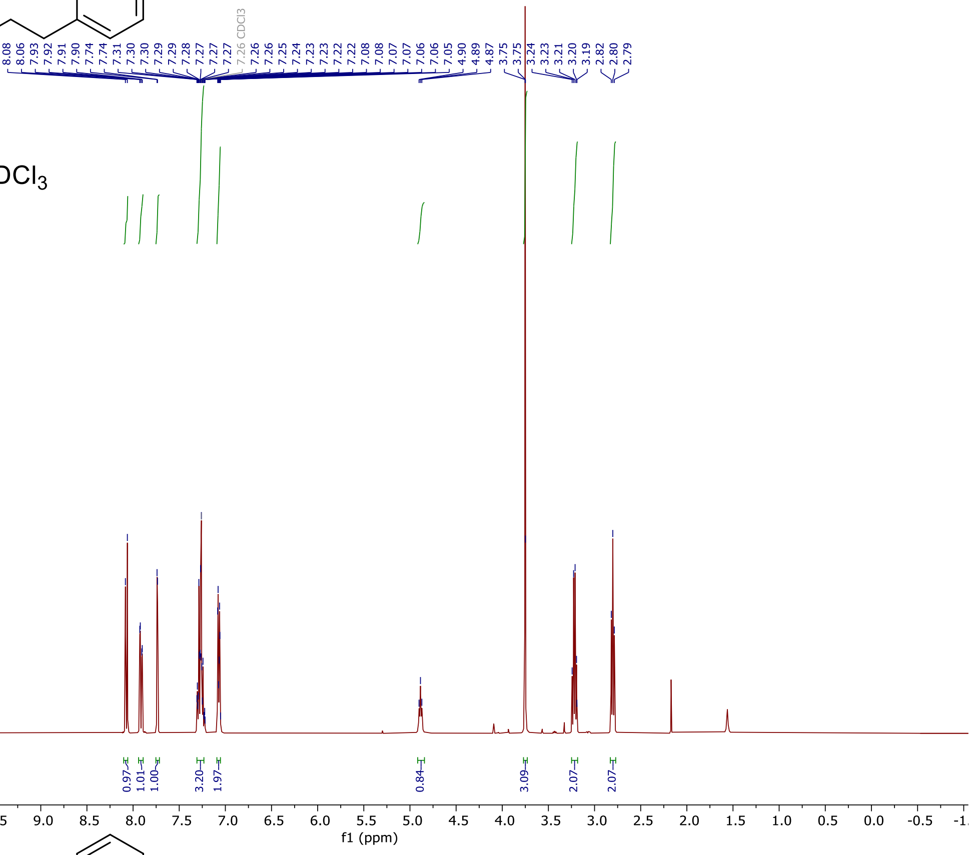

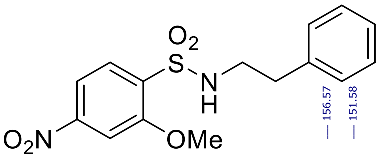

1n

101 MHz, $CDCl_3$

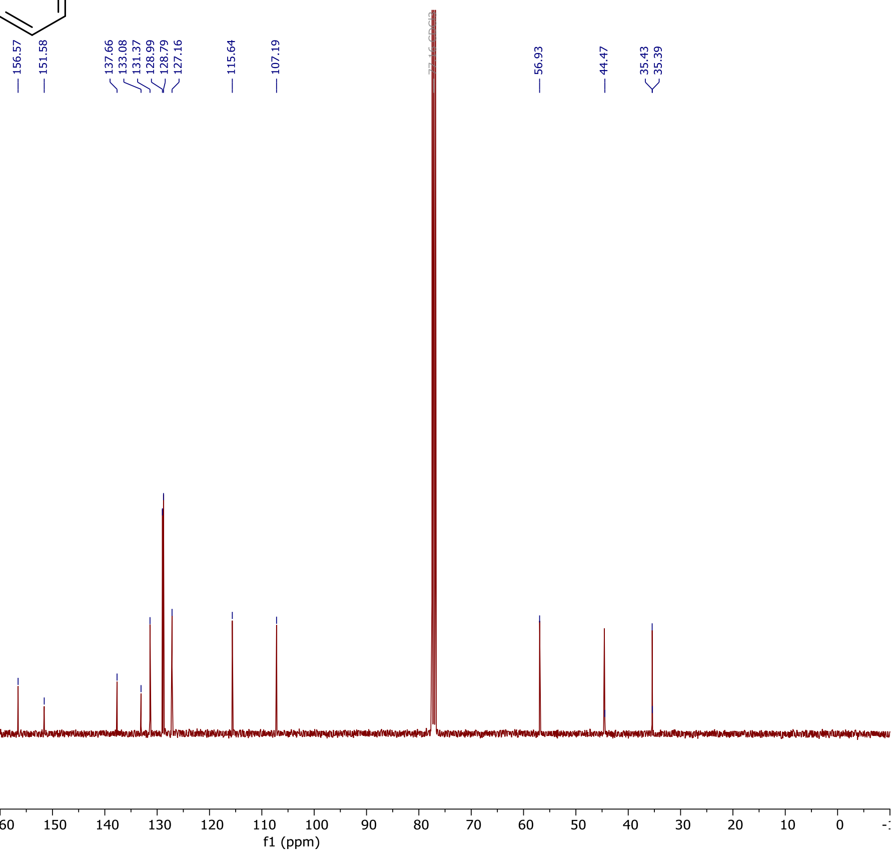

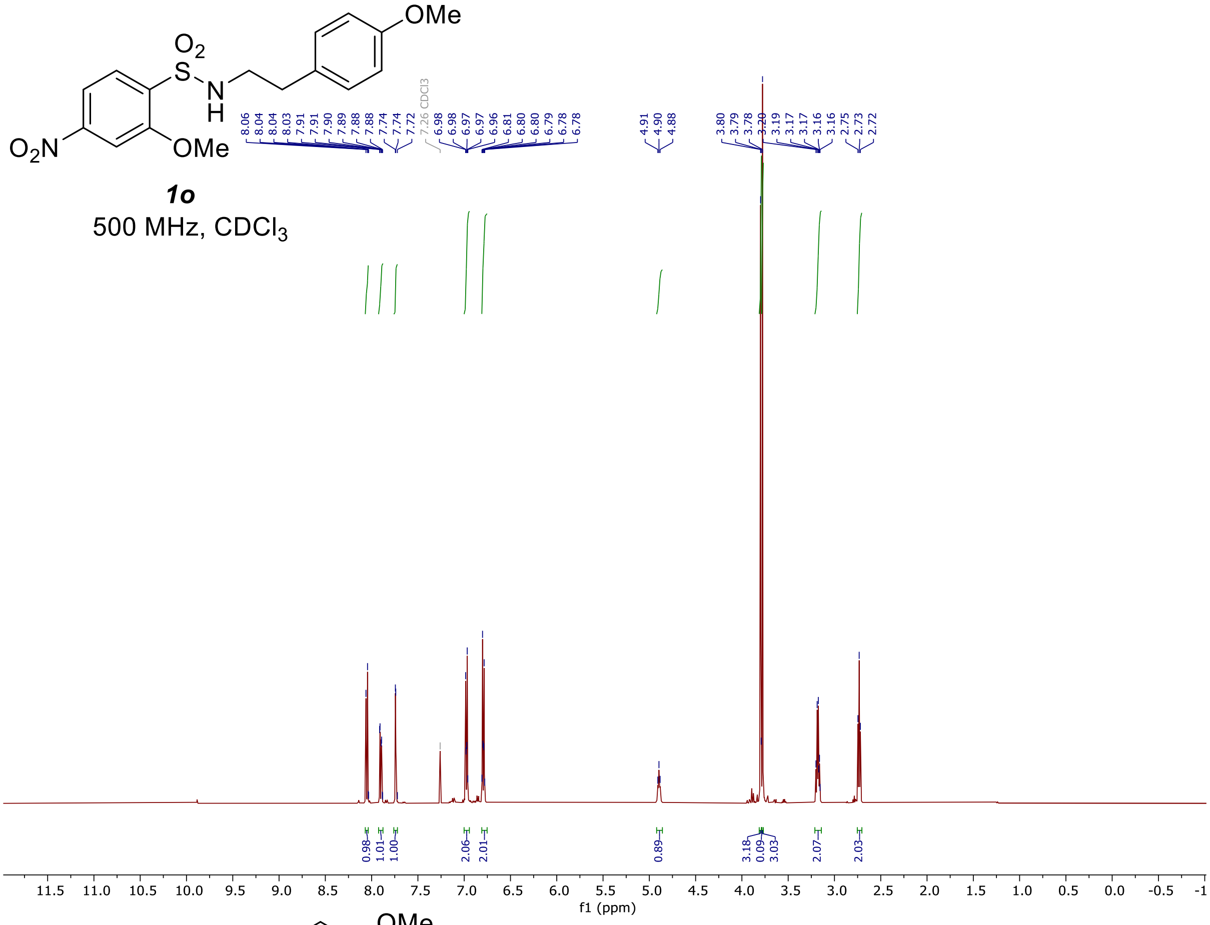

OMe
O2
S
N
H
O2N
OMe
1o
500 MHz, CDCl3
f1 (ppm)

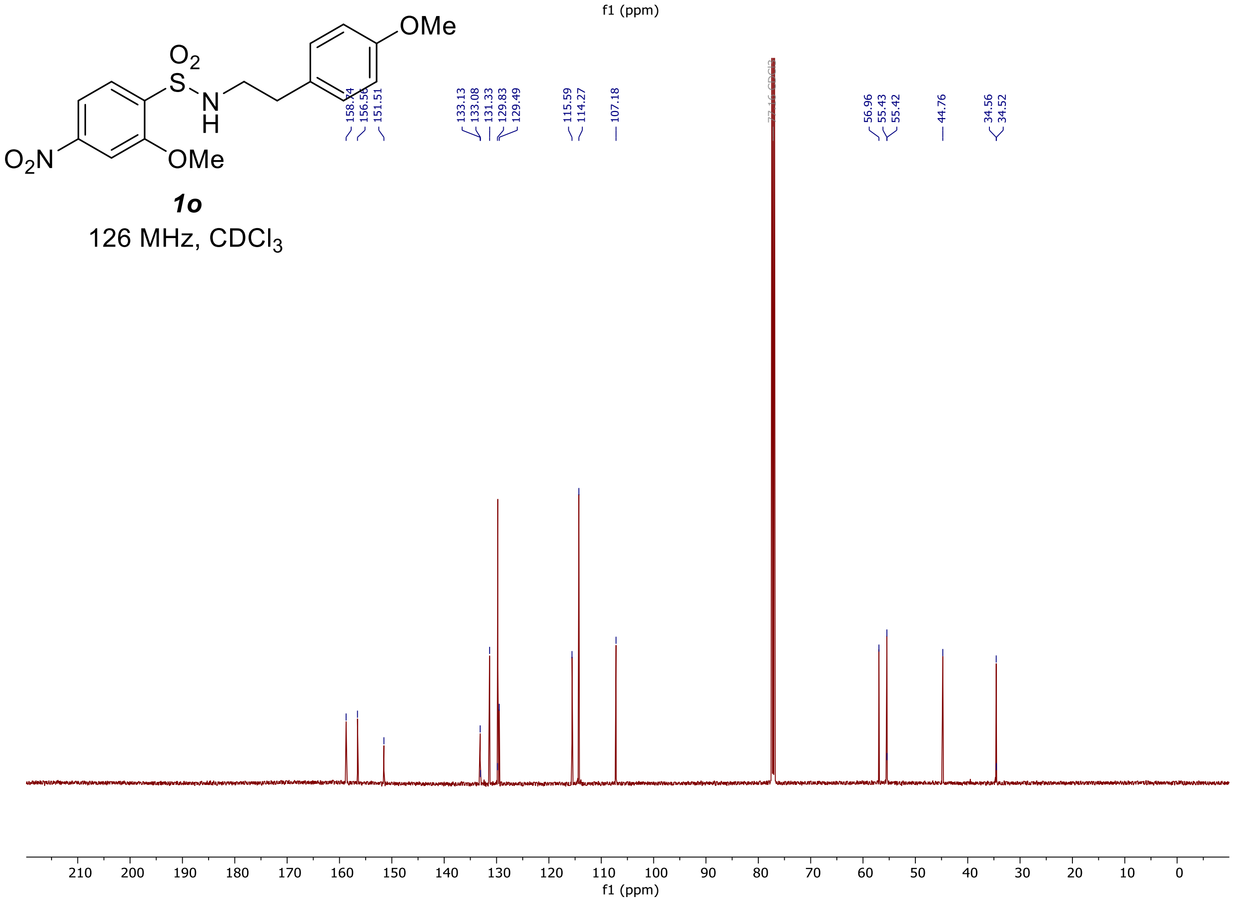

OMe
O2
S
N
H
O2N
OMe
1o
126 MHz, CDCl3
f1 (ppm)

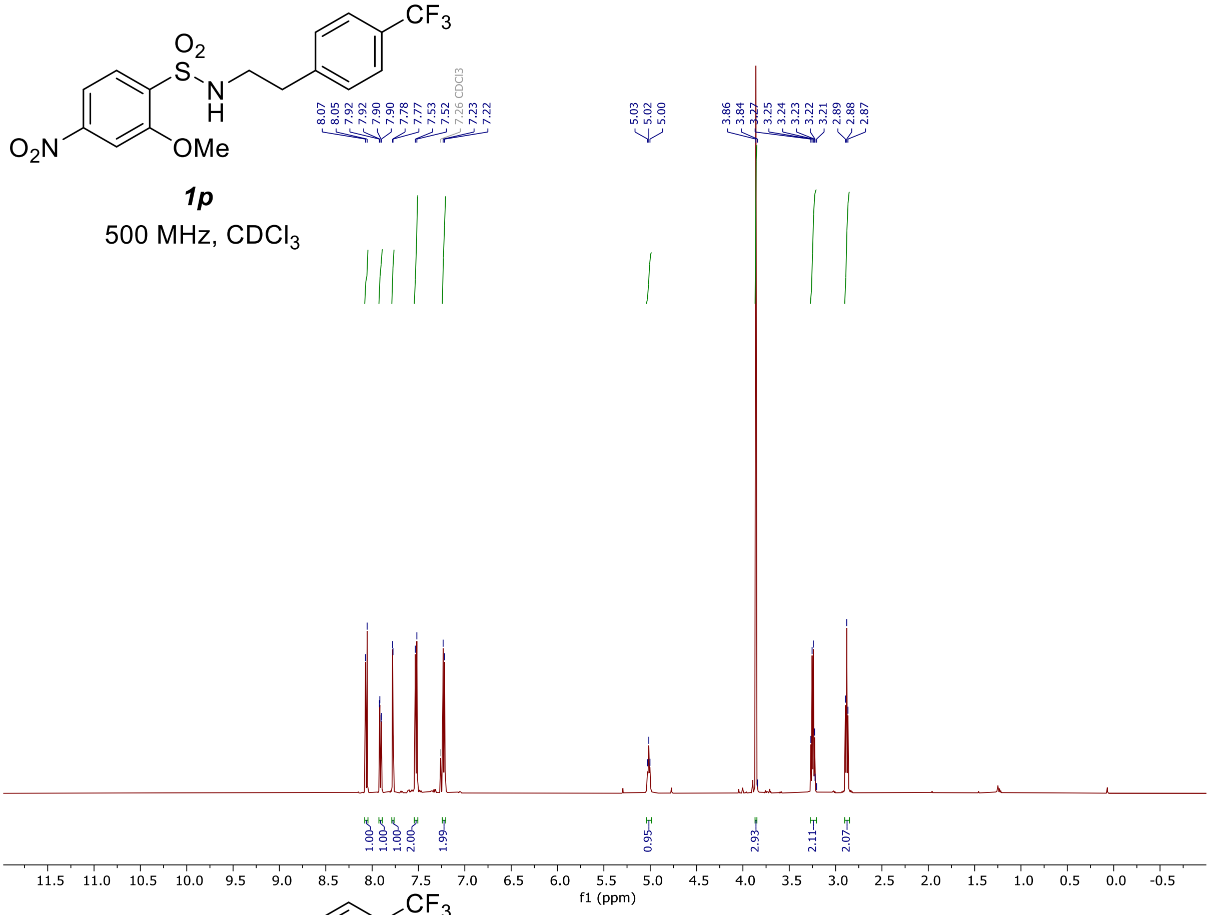

1p

500 MHz, $CDCl_3$

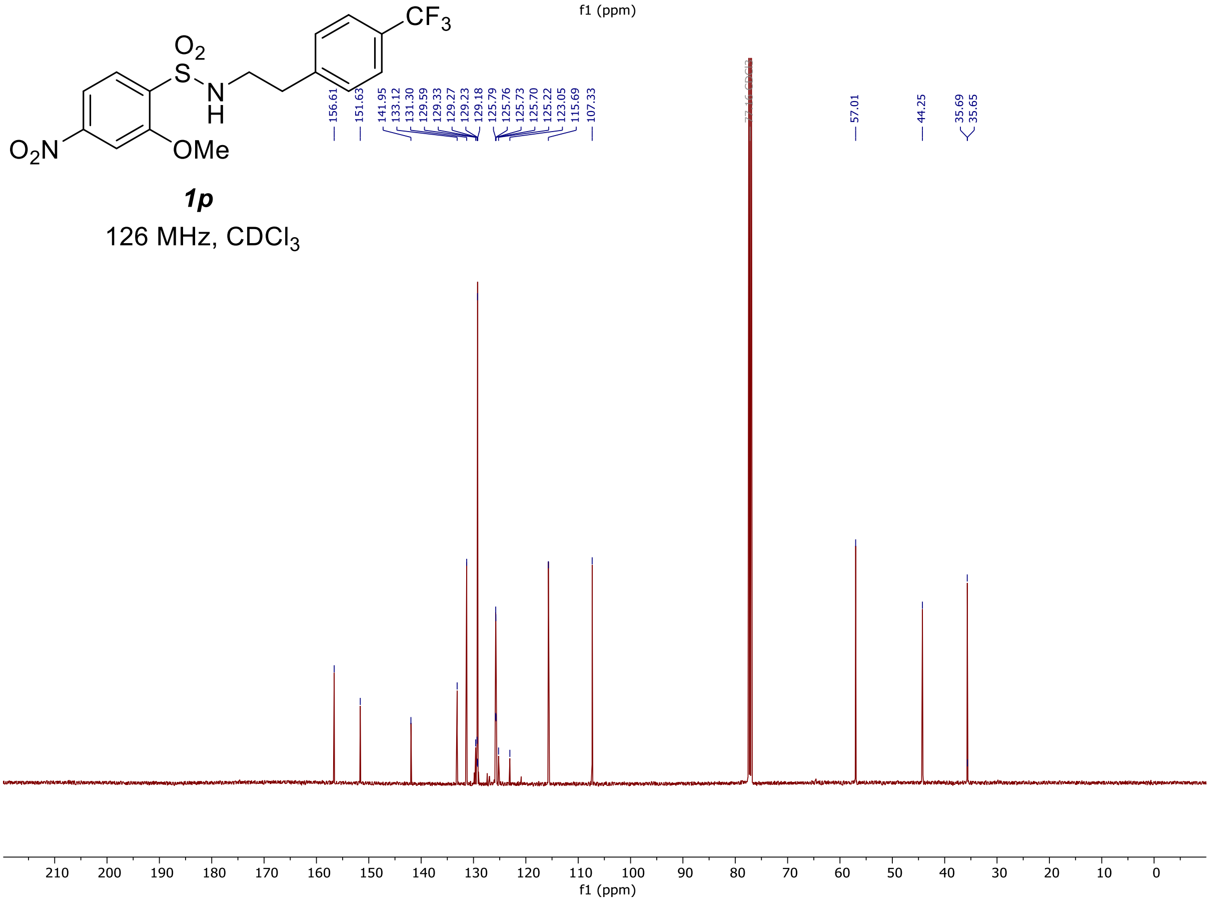

1p

126 MHz, $CDCl_3$

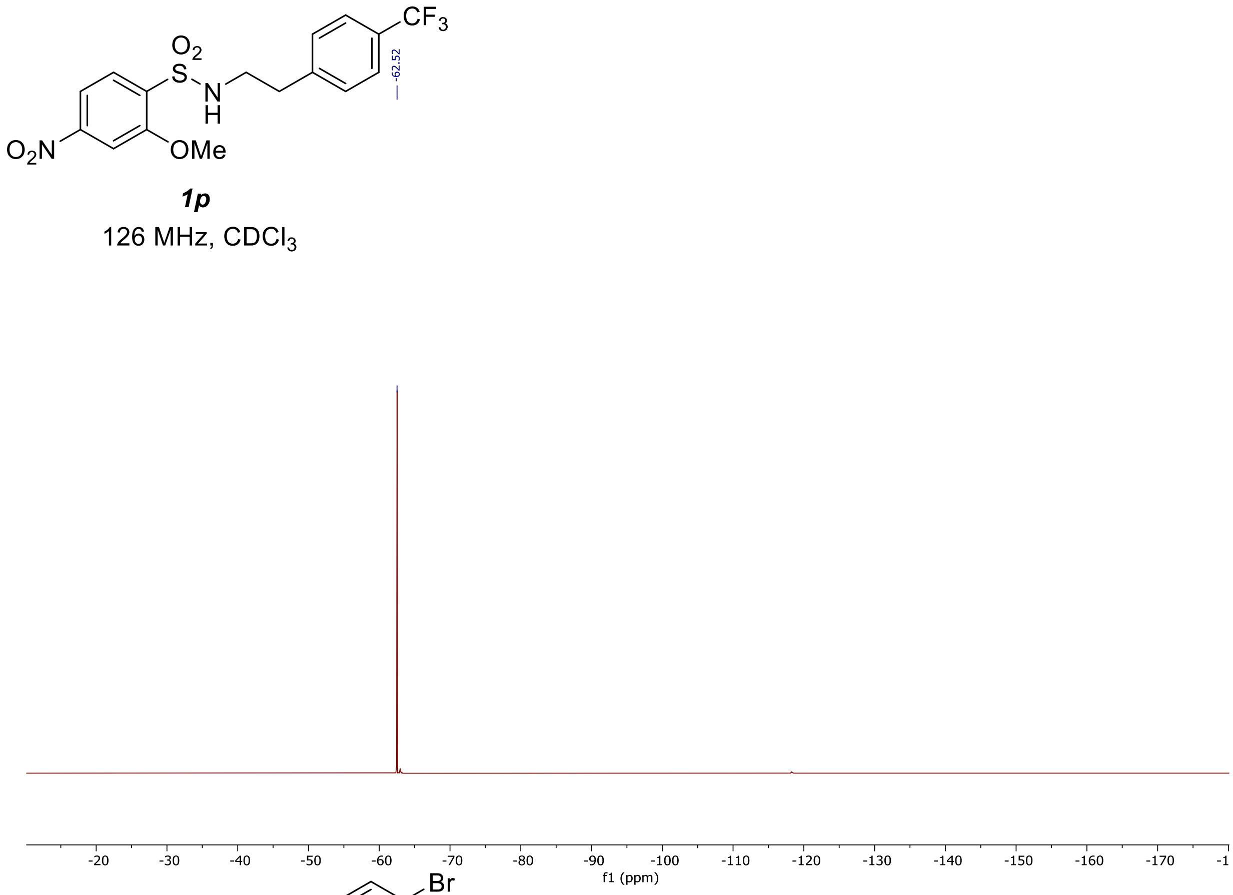

1p
126 MHz, $CDCl_3$

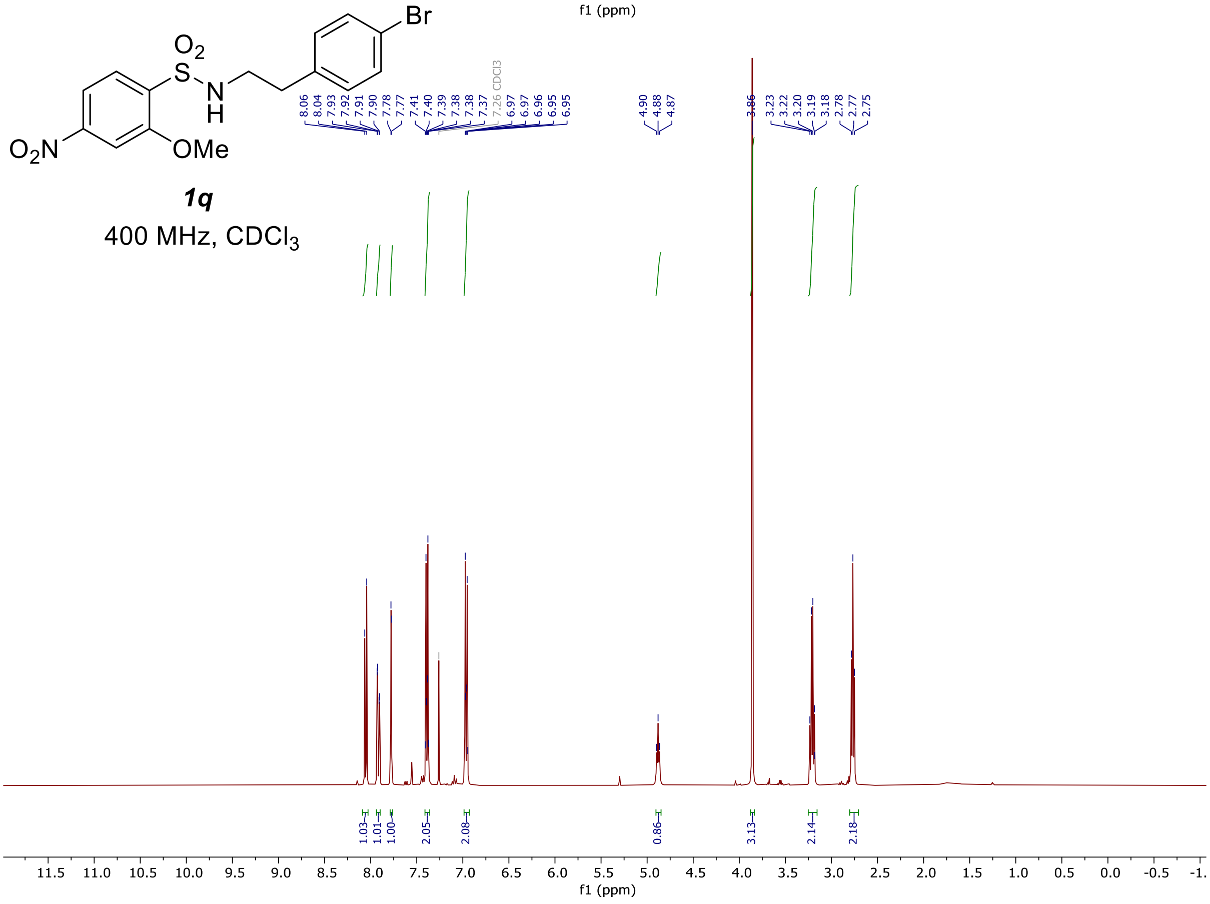

1q
400 MHz, $CDCl_3$

1q

101 MHz, $CDCl_3$

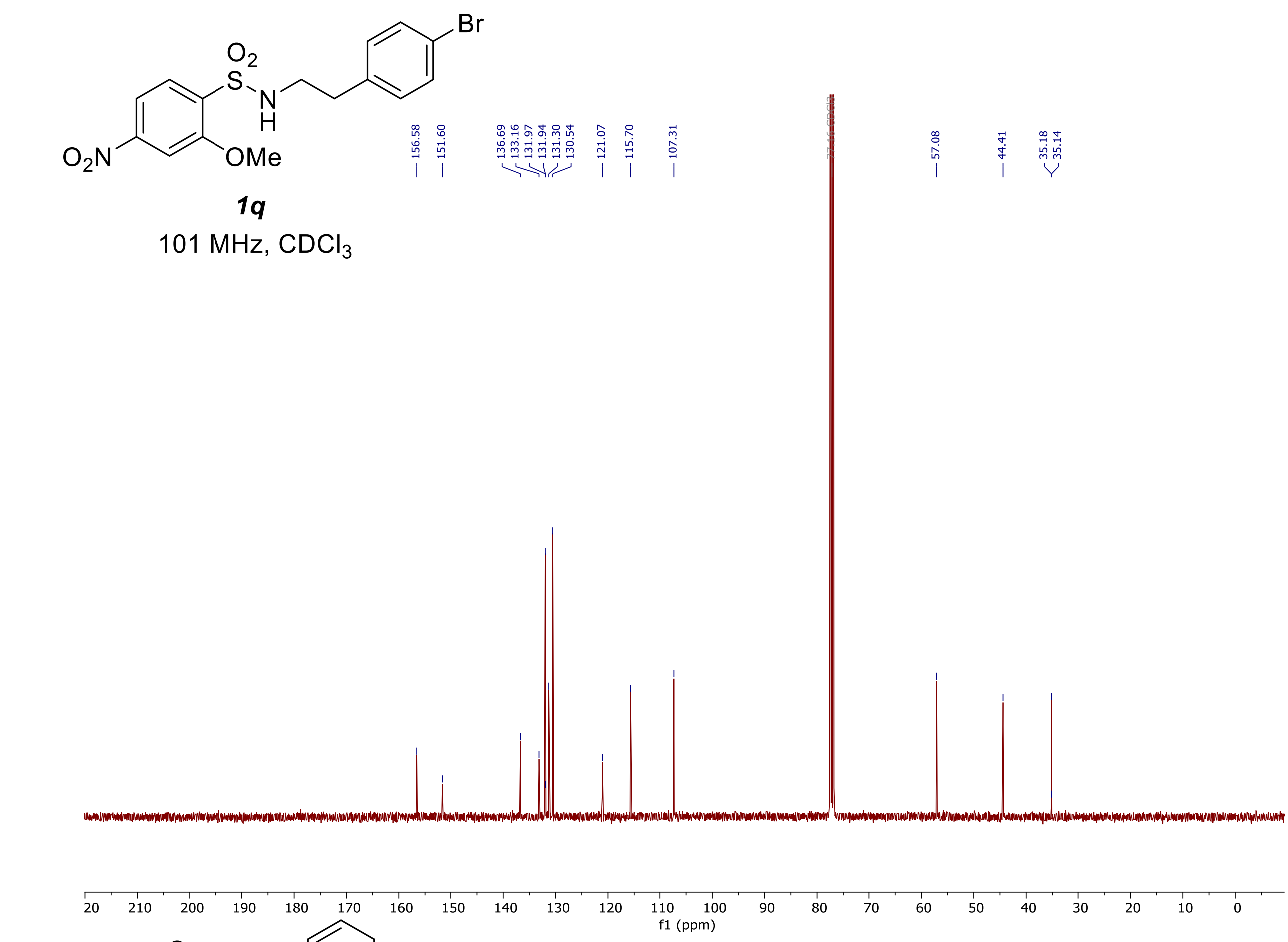

1r

400 MHz, $CDCl_3$

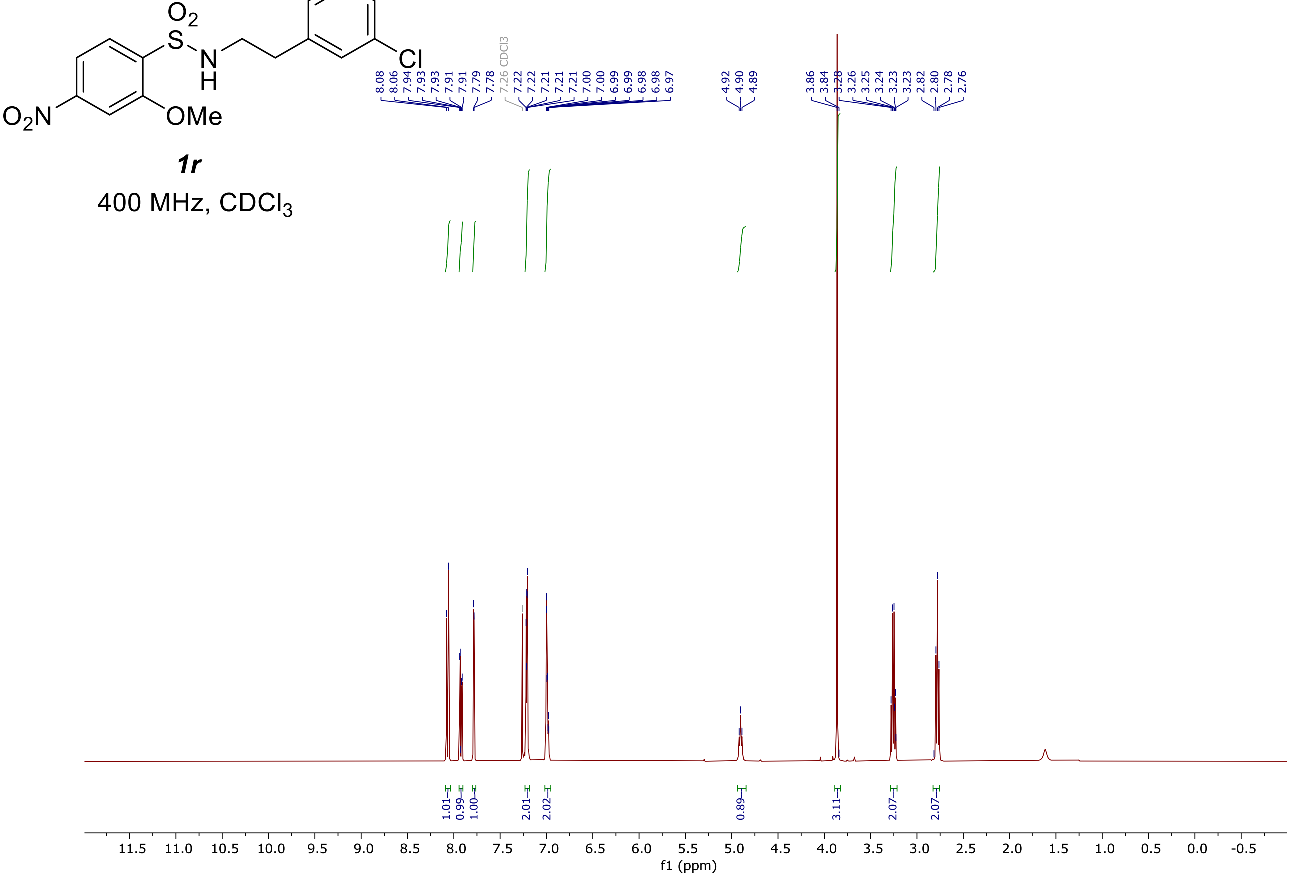

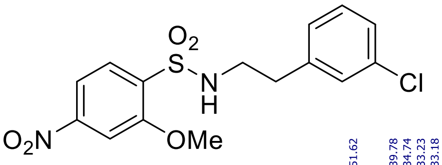

1r

101 MHz, $CDCl_3$

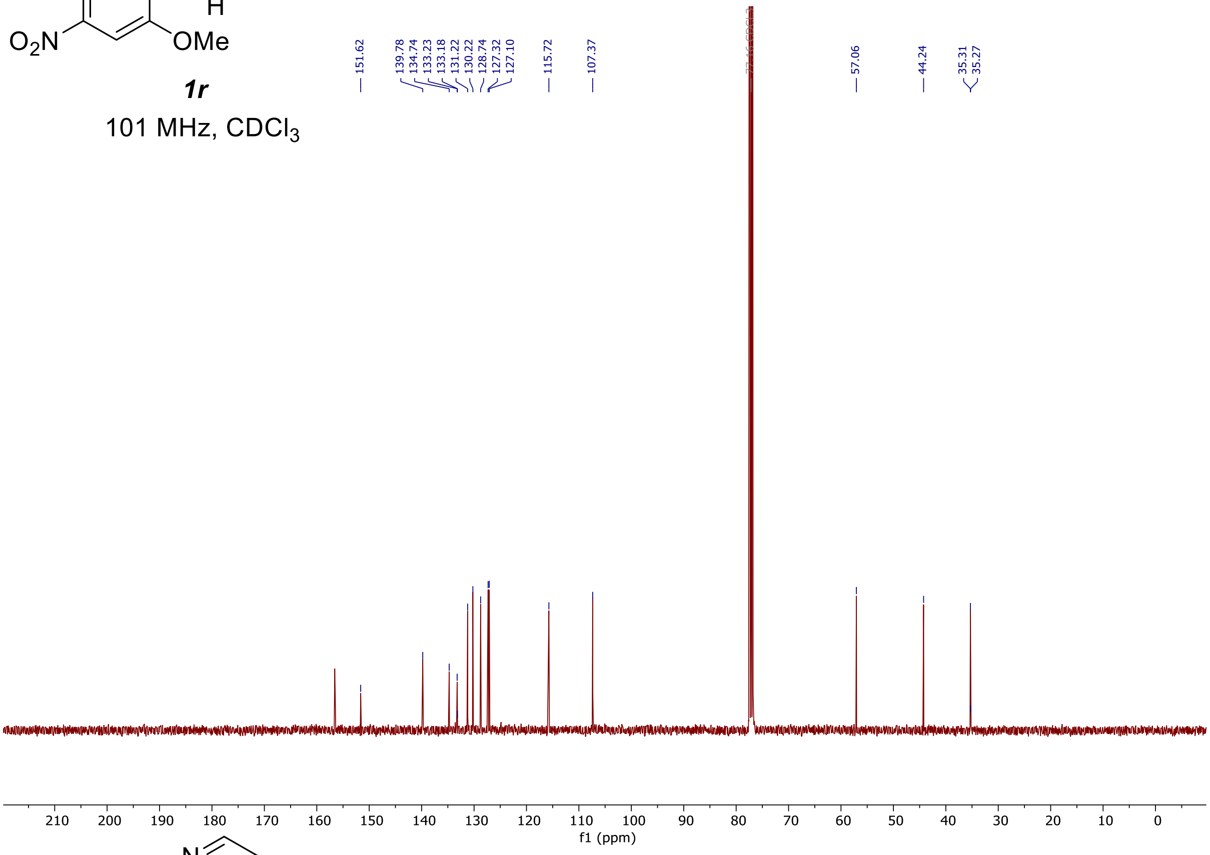

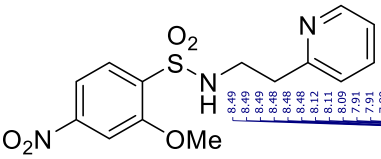

1s

500 MHz, $CDCl_3$

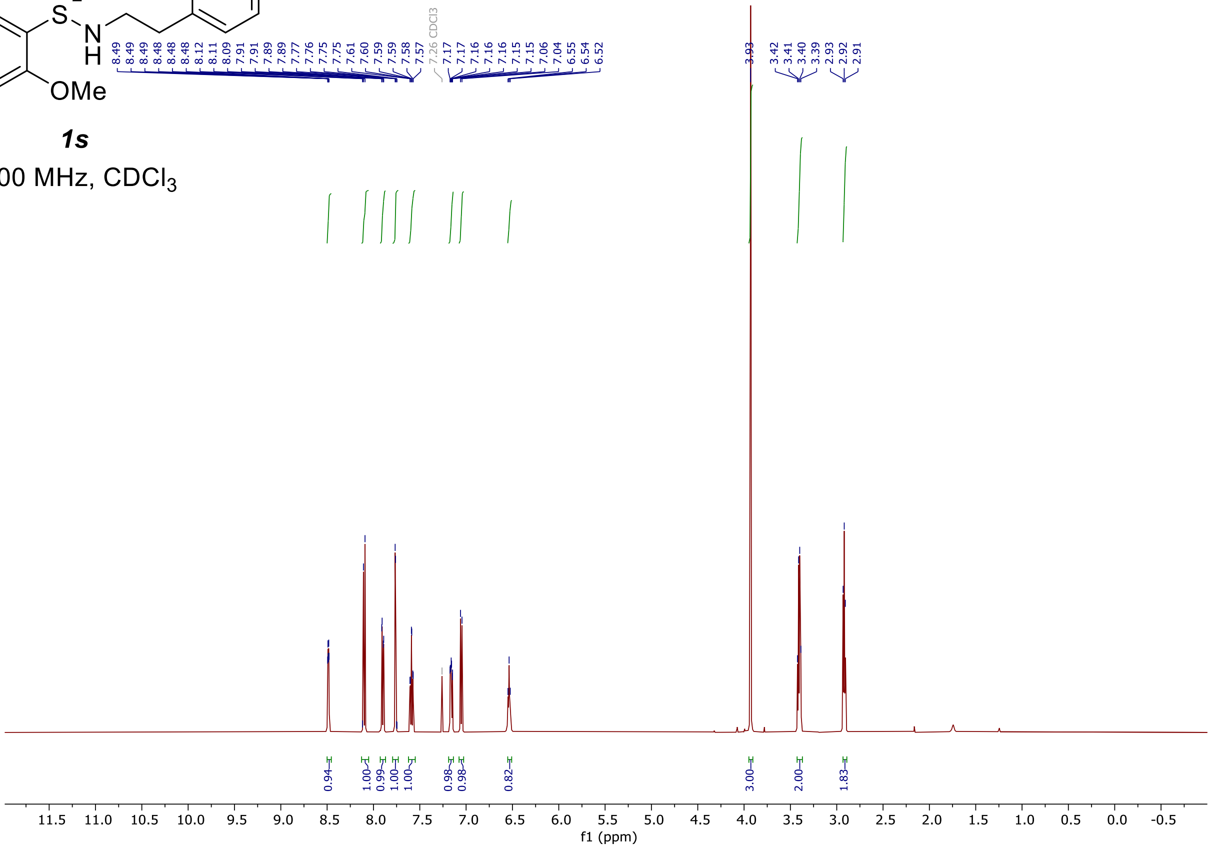

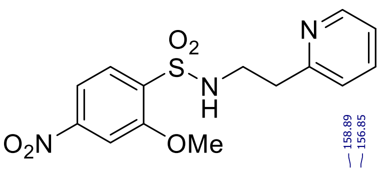

1s

126 MHz, $CDCl_3$

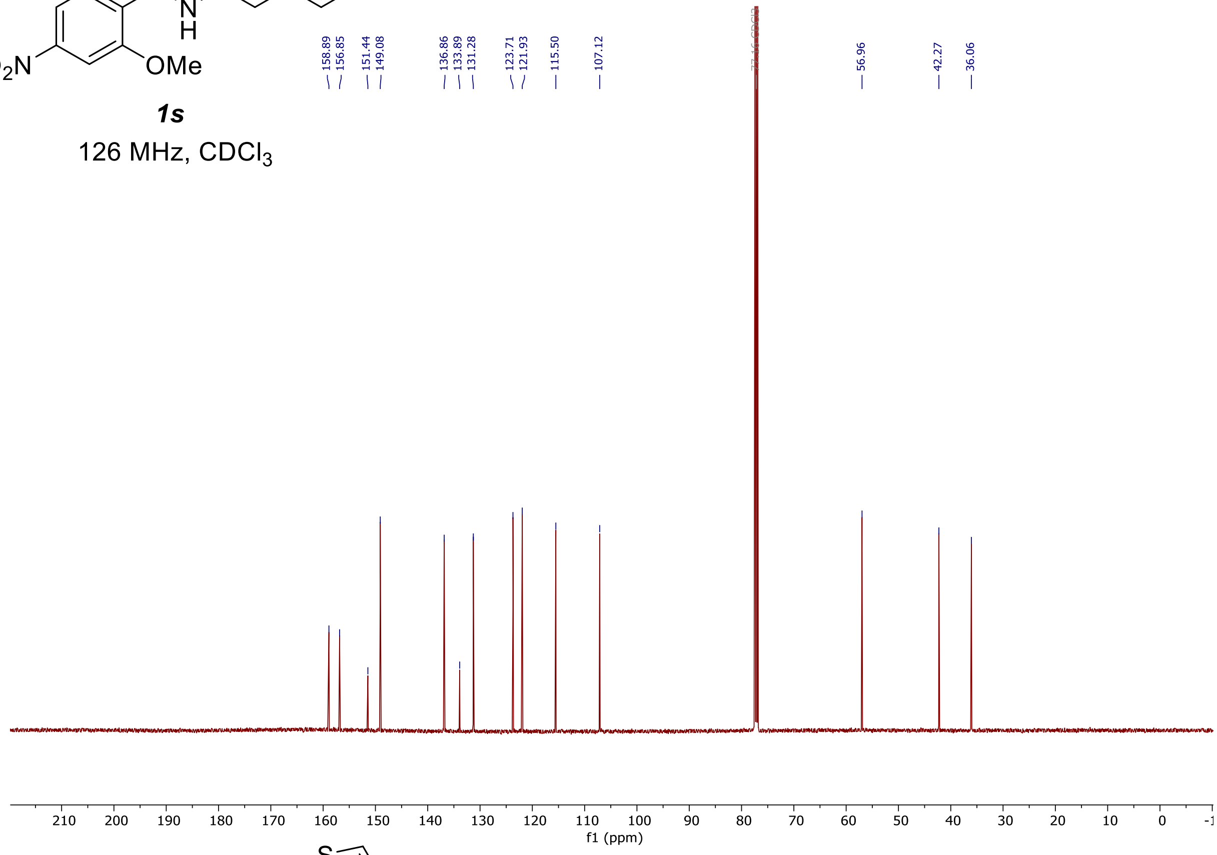

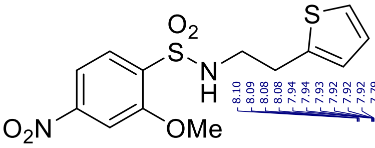

1t

500 MHz, $CDCl_3$

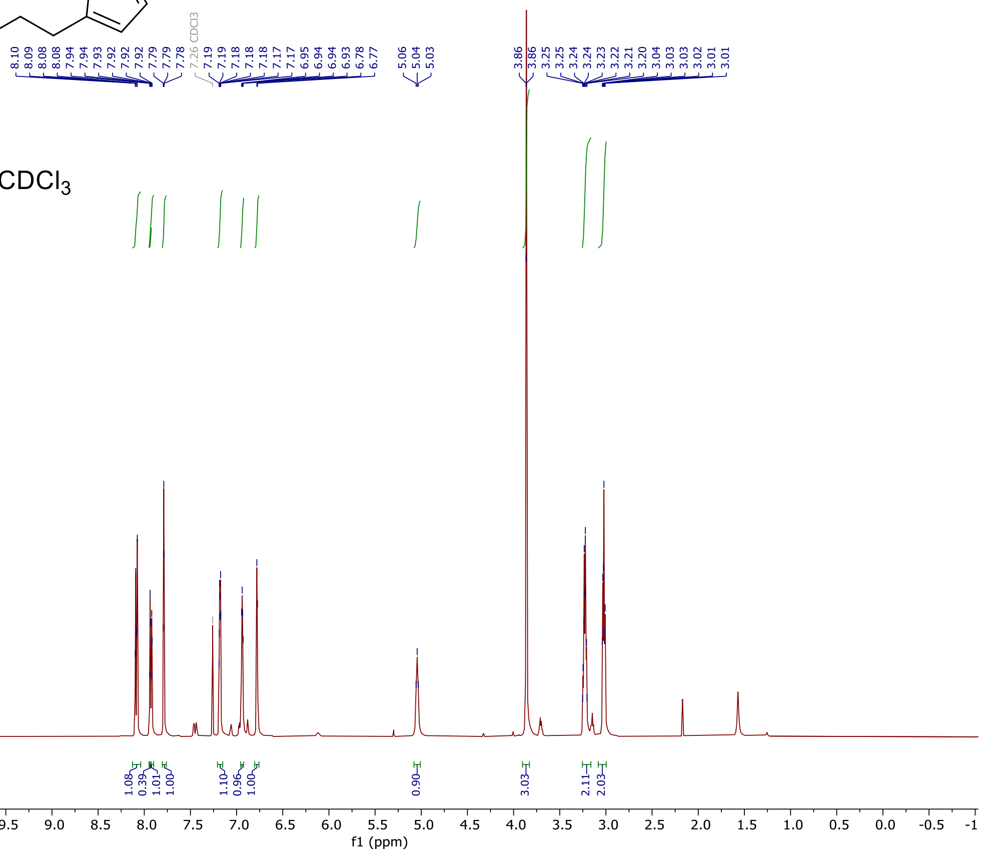

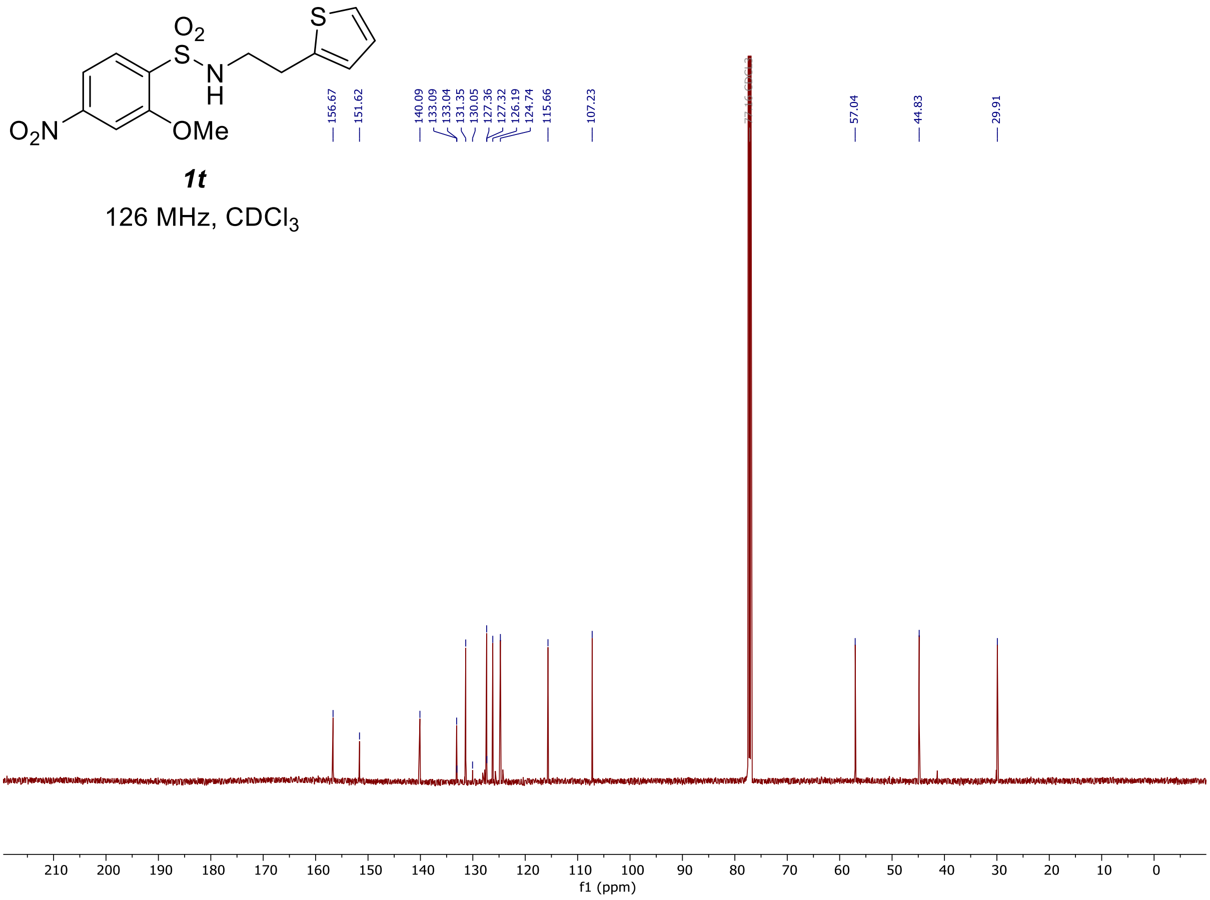

1t

126 MHz, $CDCl_3$

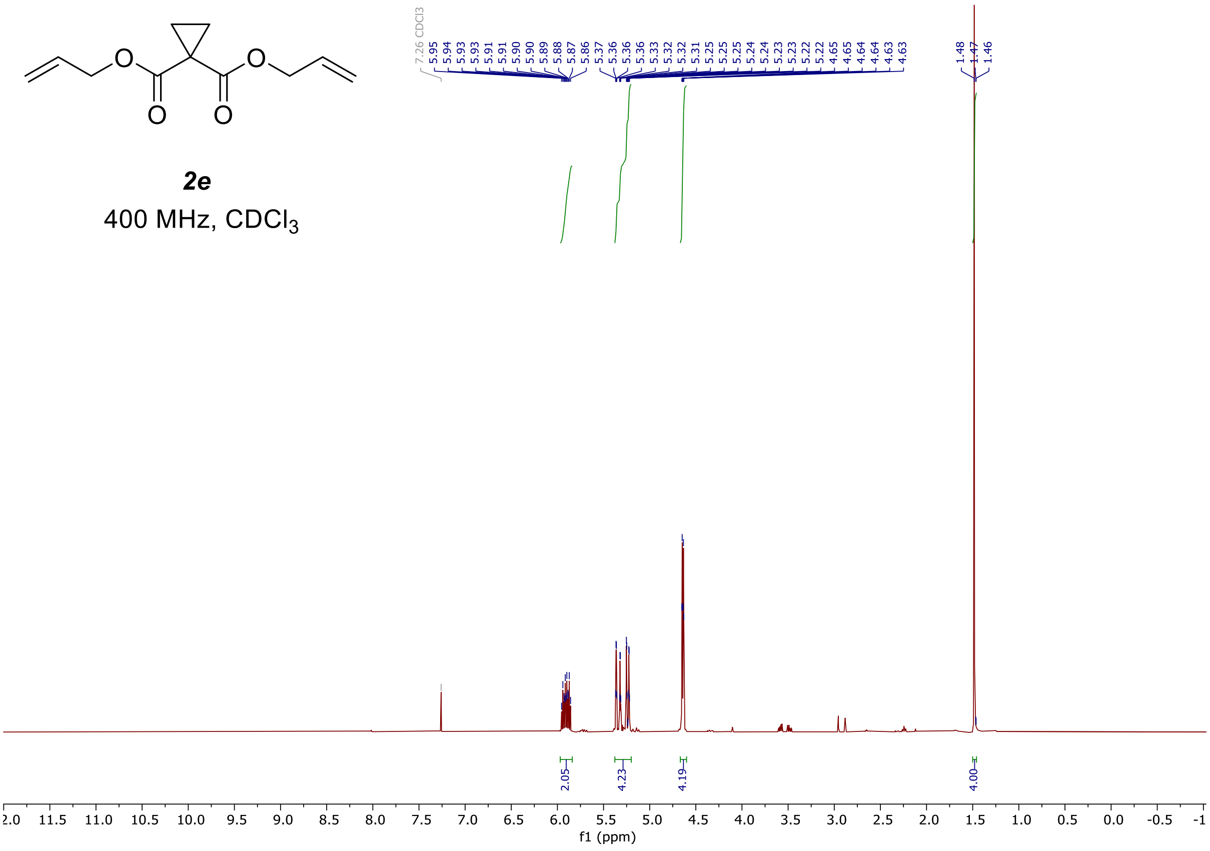

2e

400 MHz, $CDCl_3$

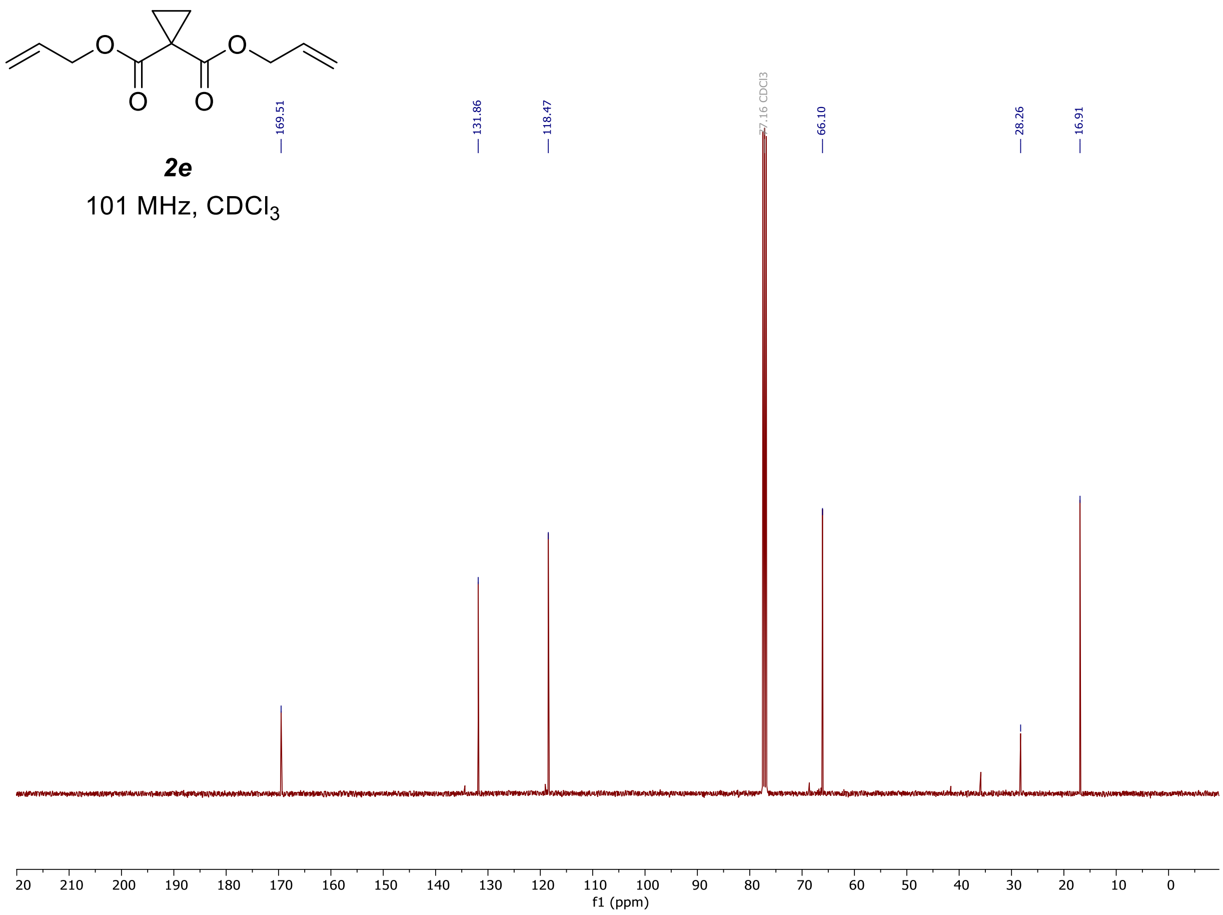

2e

101 MHz, $CDCl_3$

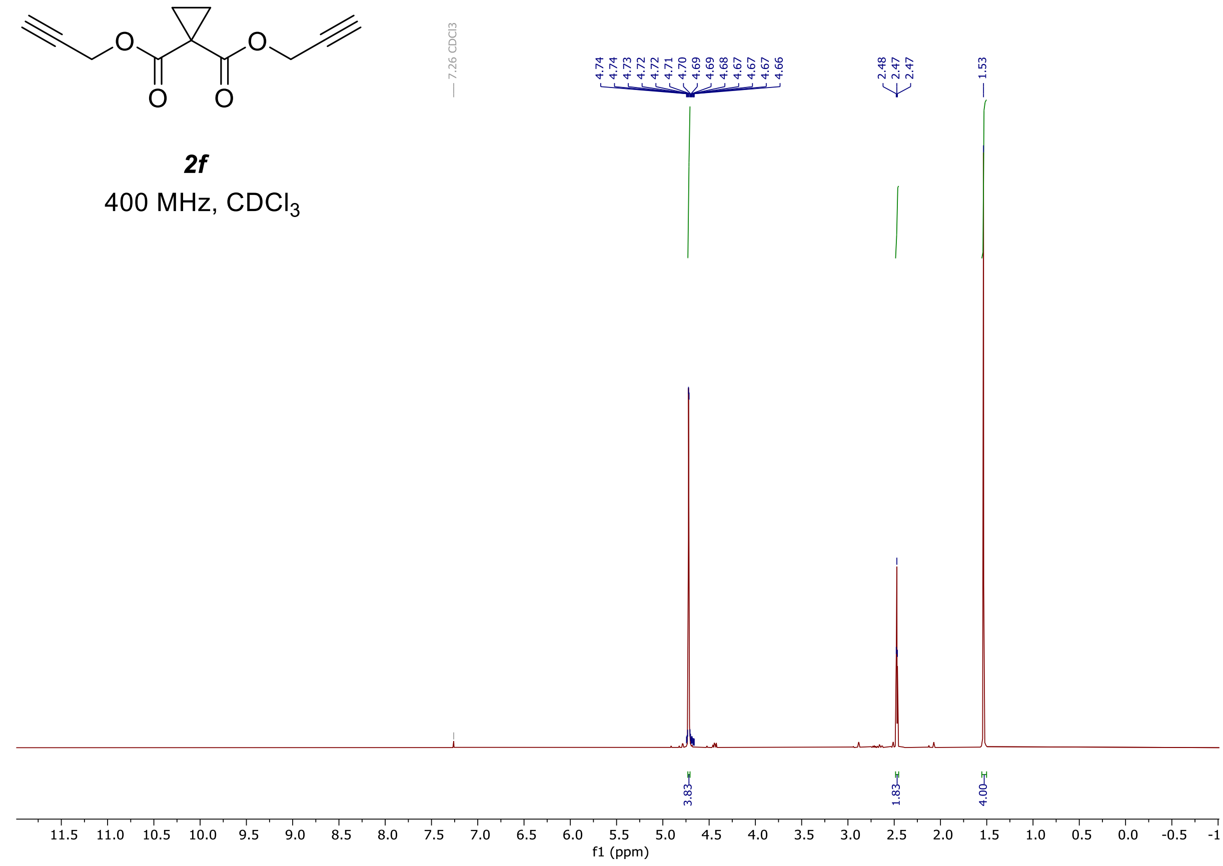

2f

400 MHz, $CDCl_3$

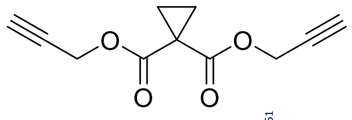

2f

101 MHz, $CDCl_3$

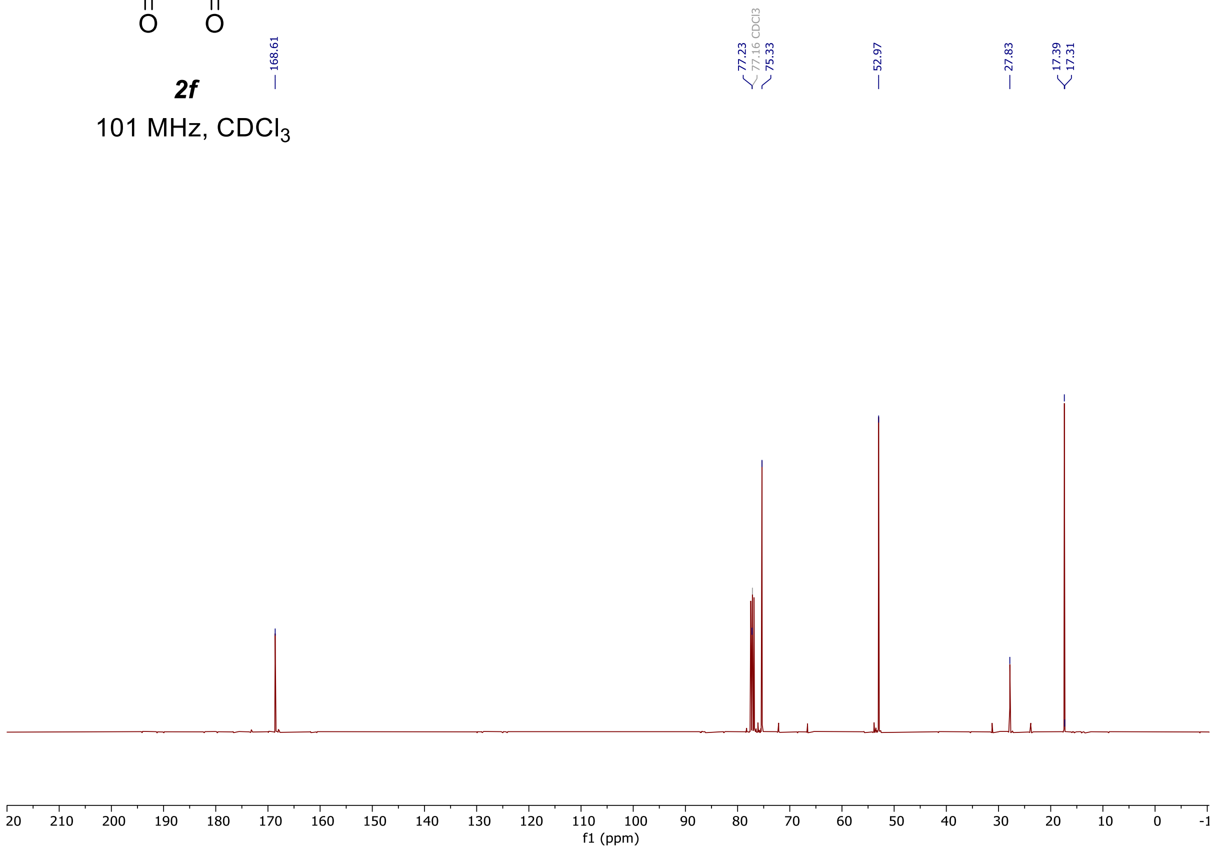

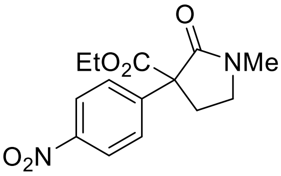

3a

500 MHz, $CDCl_3$

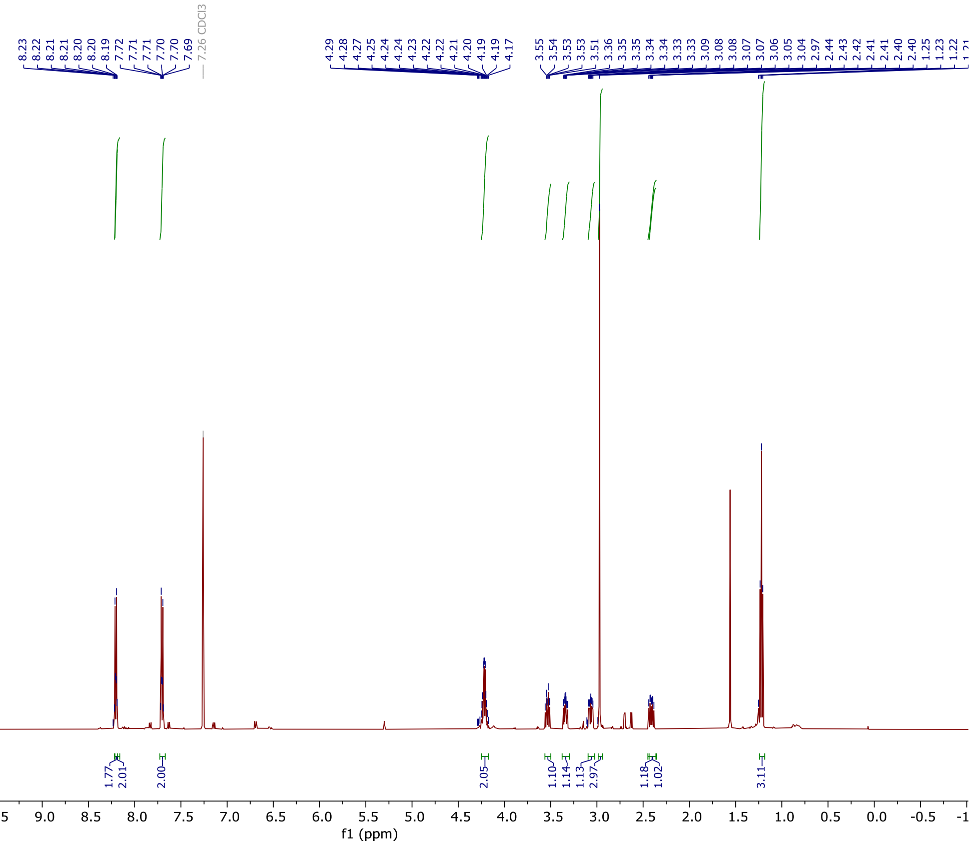

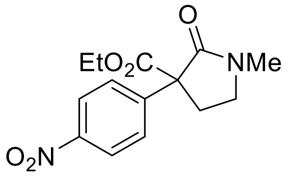

3a

126 MHz, $CDCl_3$

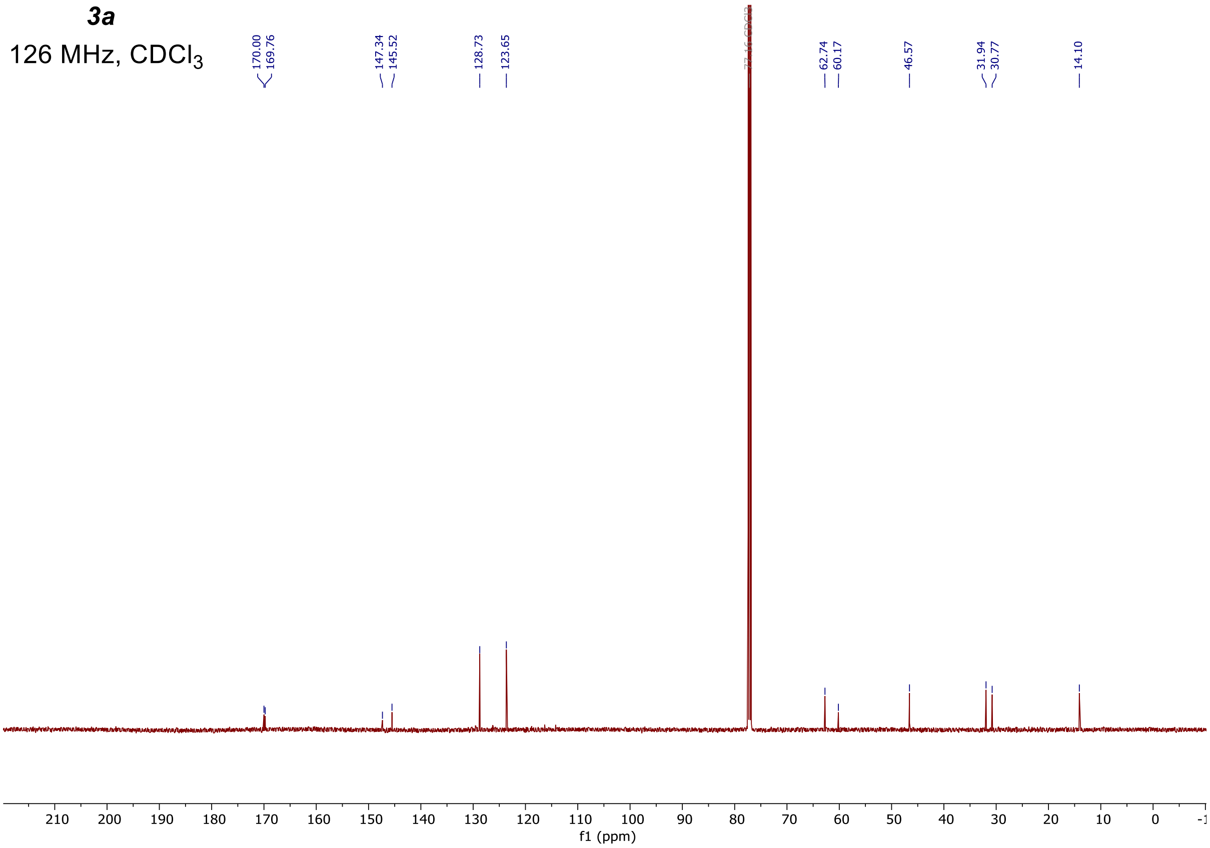

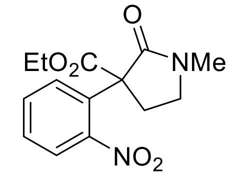

3b

400 MHz, $CDCl_3$

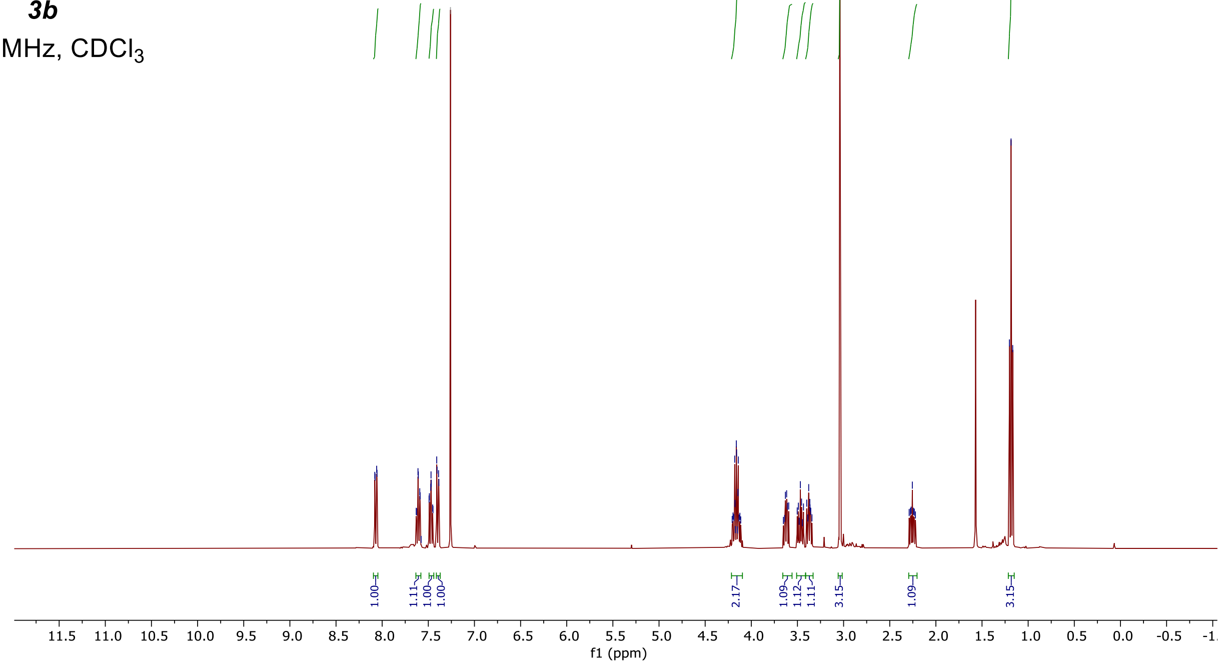

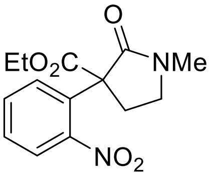

3b

101 MHz, $CDCl_3$

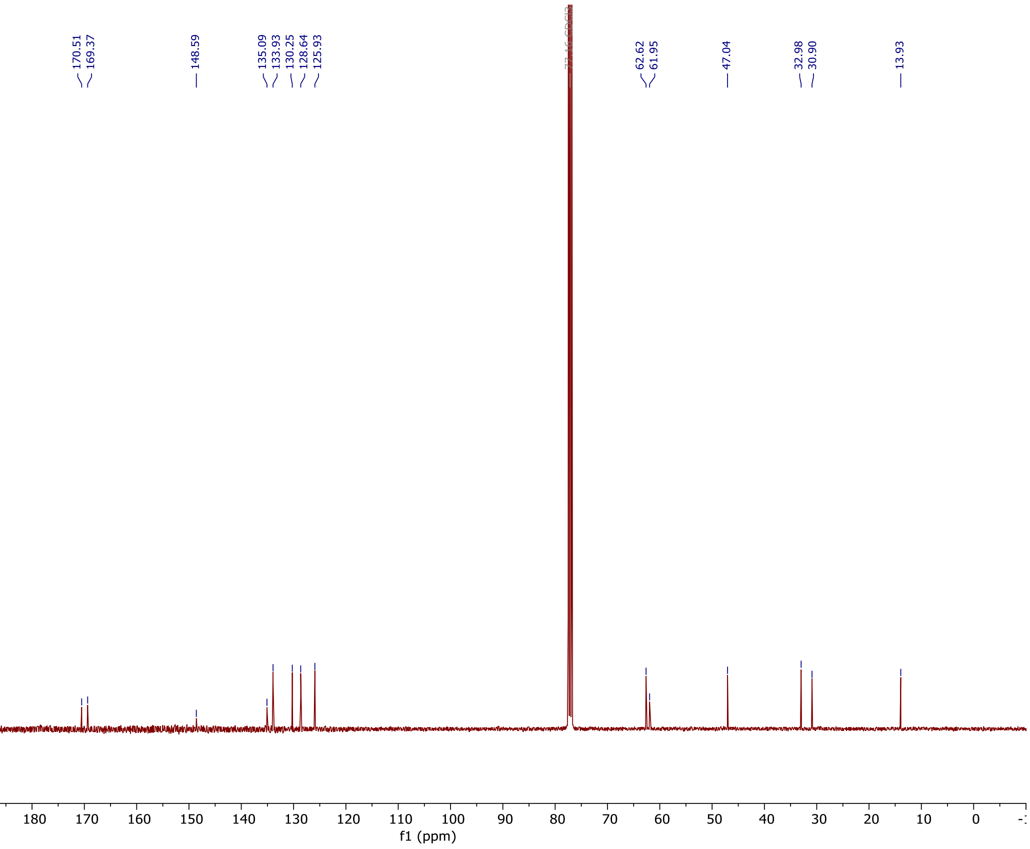

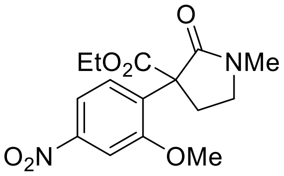

3c

400 MHz, $CDCl_3$

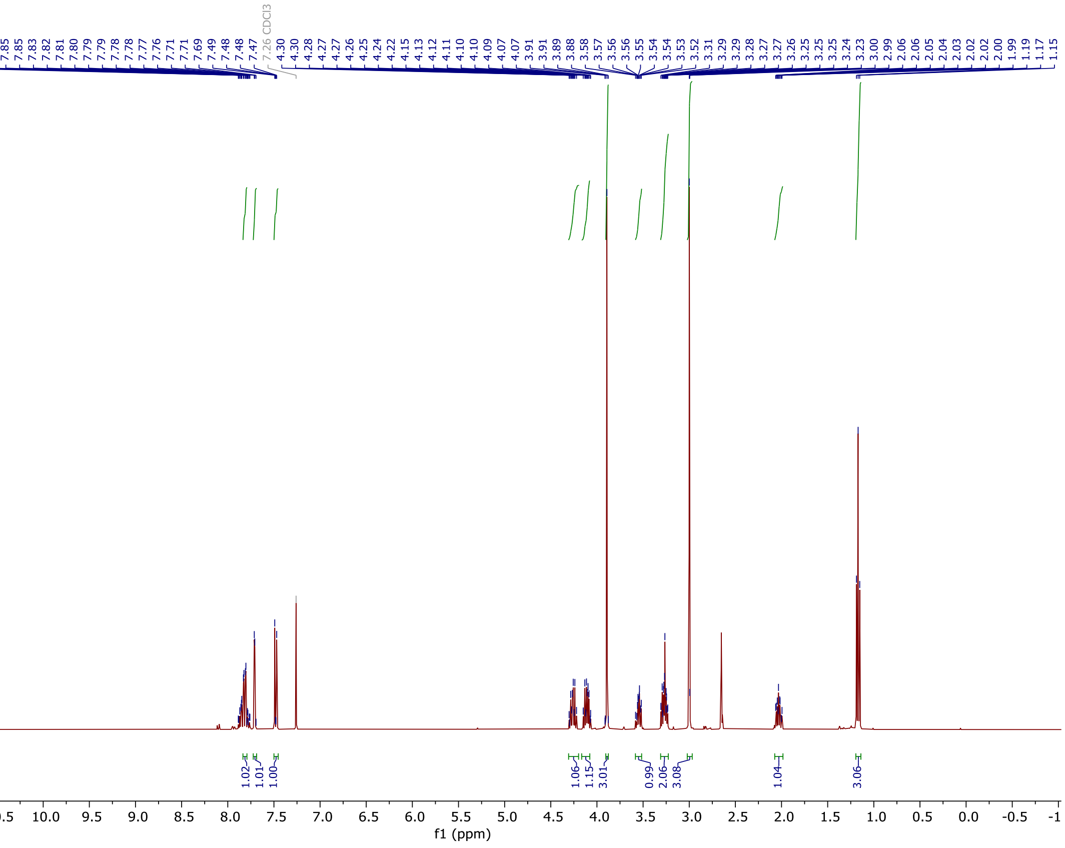

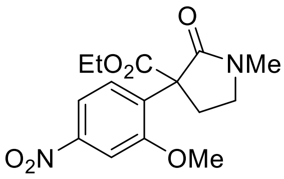

3c

101 MHz, $CDCl_3$

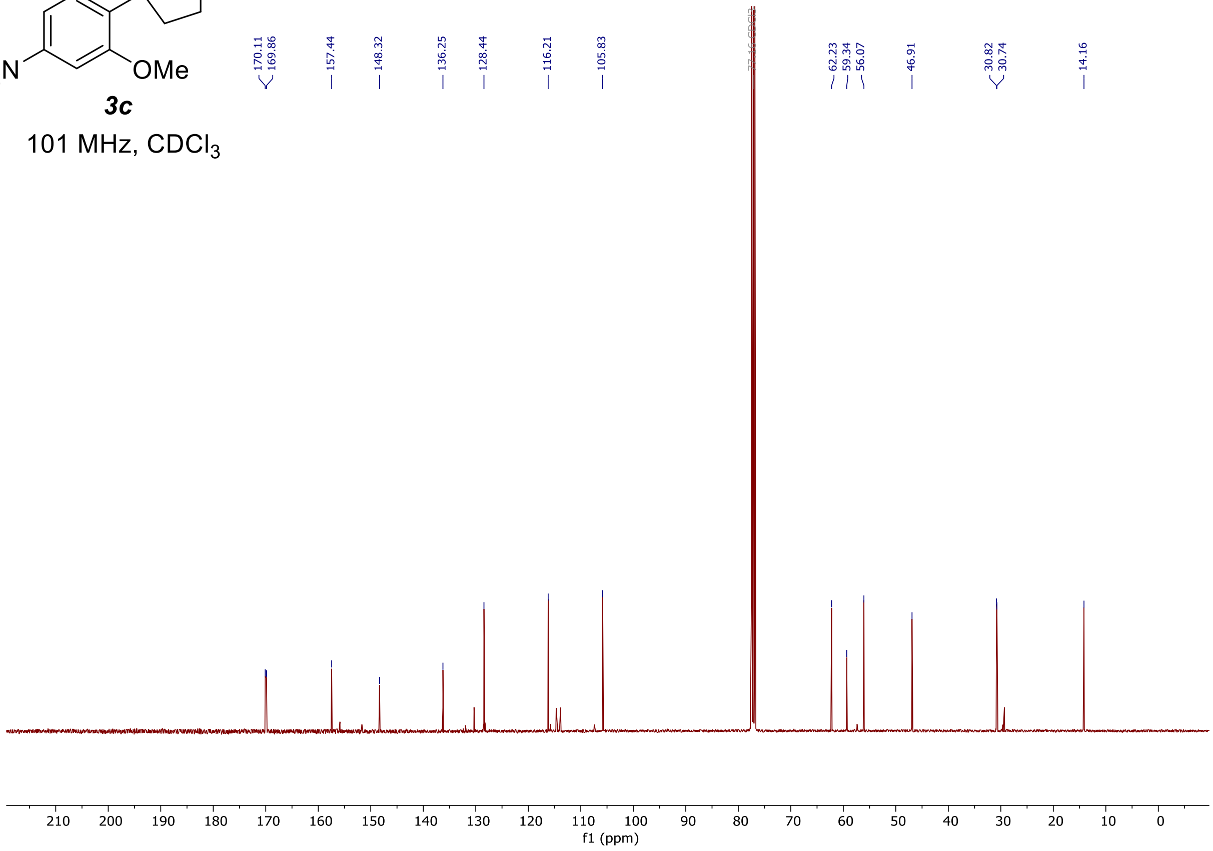

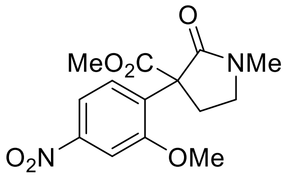

3d

400 MHz, $CDCl_3$

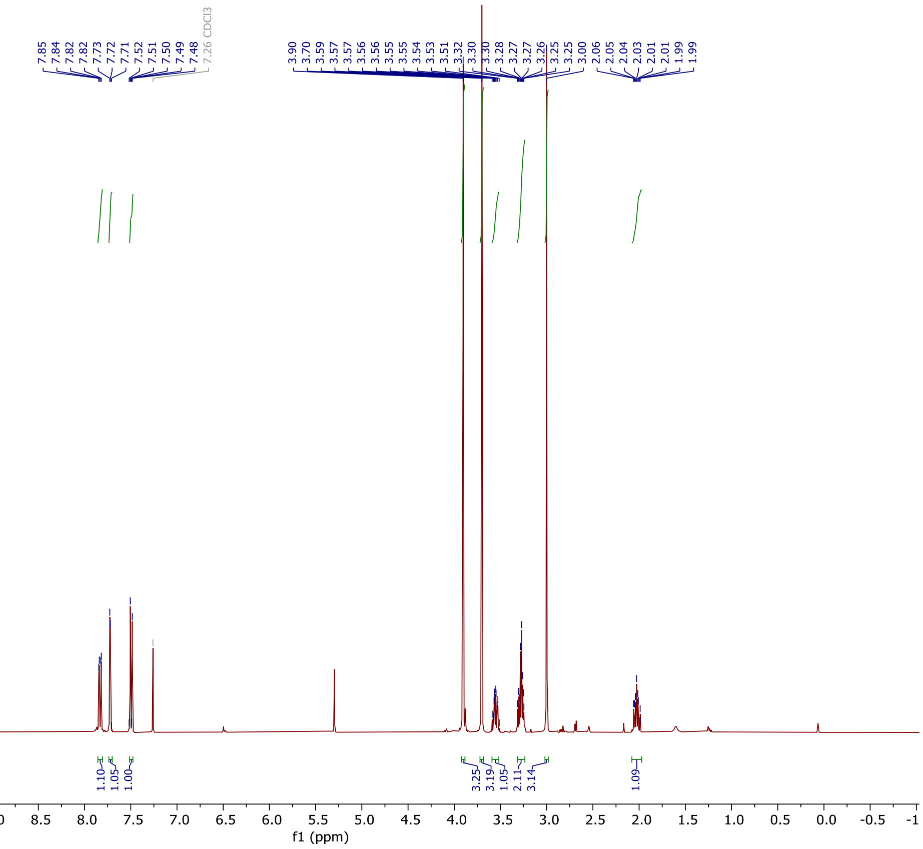

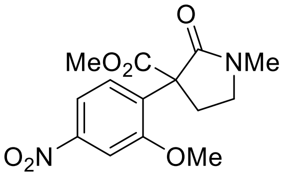

3d

101 MHz, $CDCl_3$

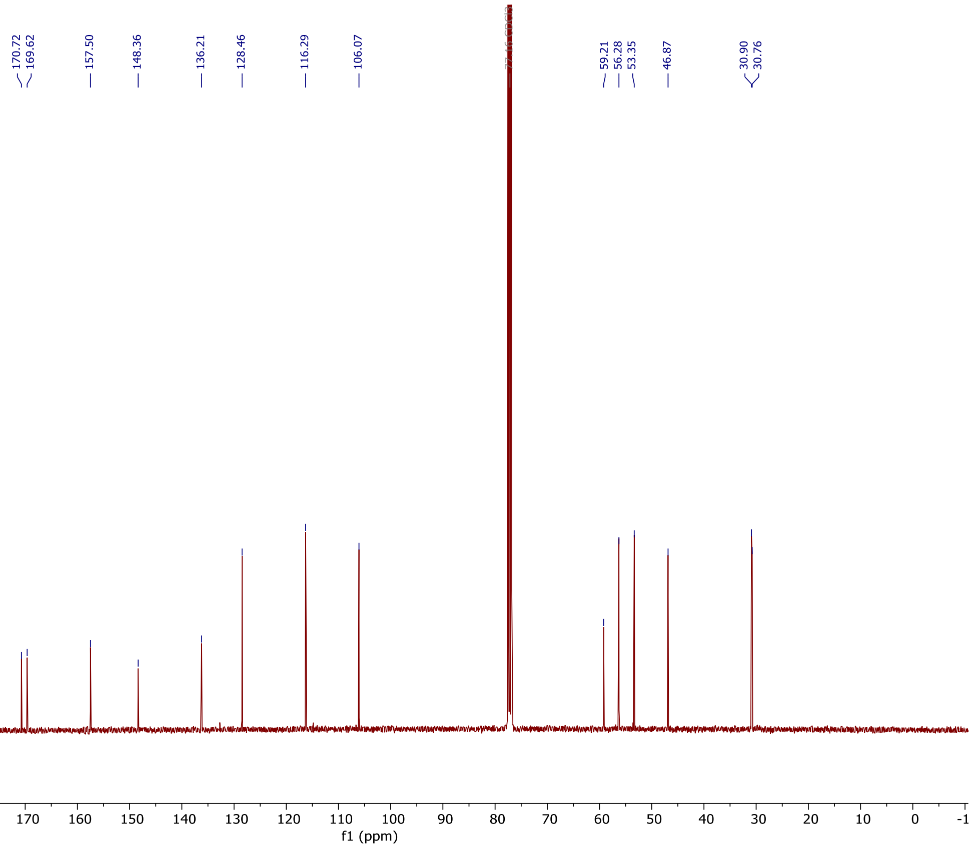

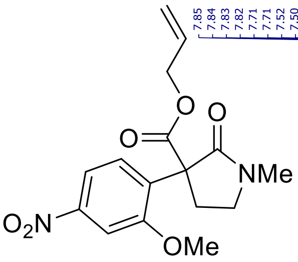

3e

400 MHz, $CDCl_3$

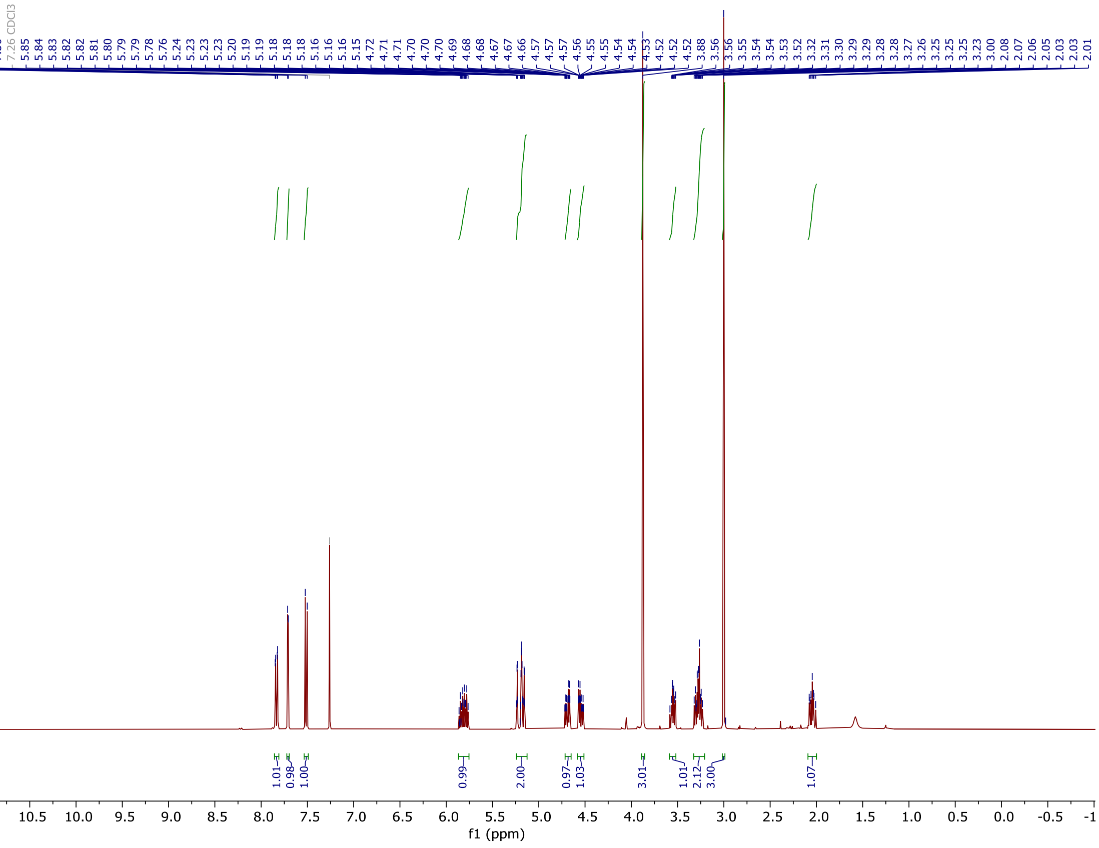

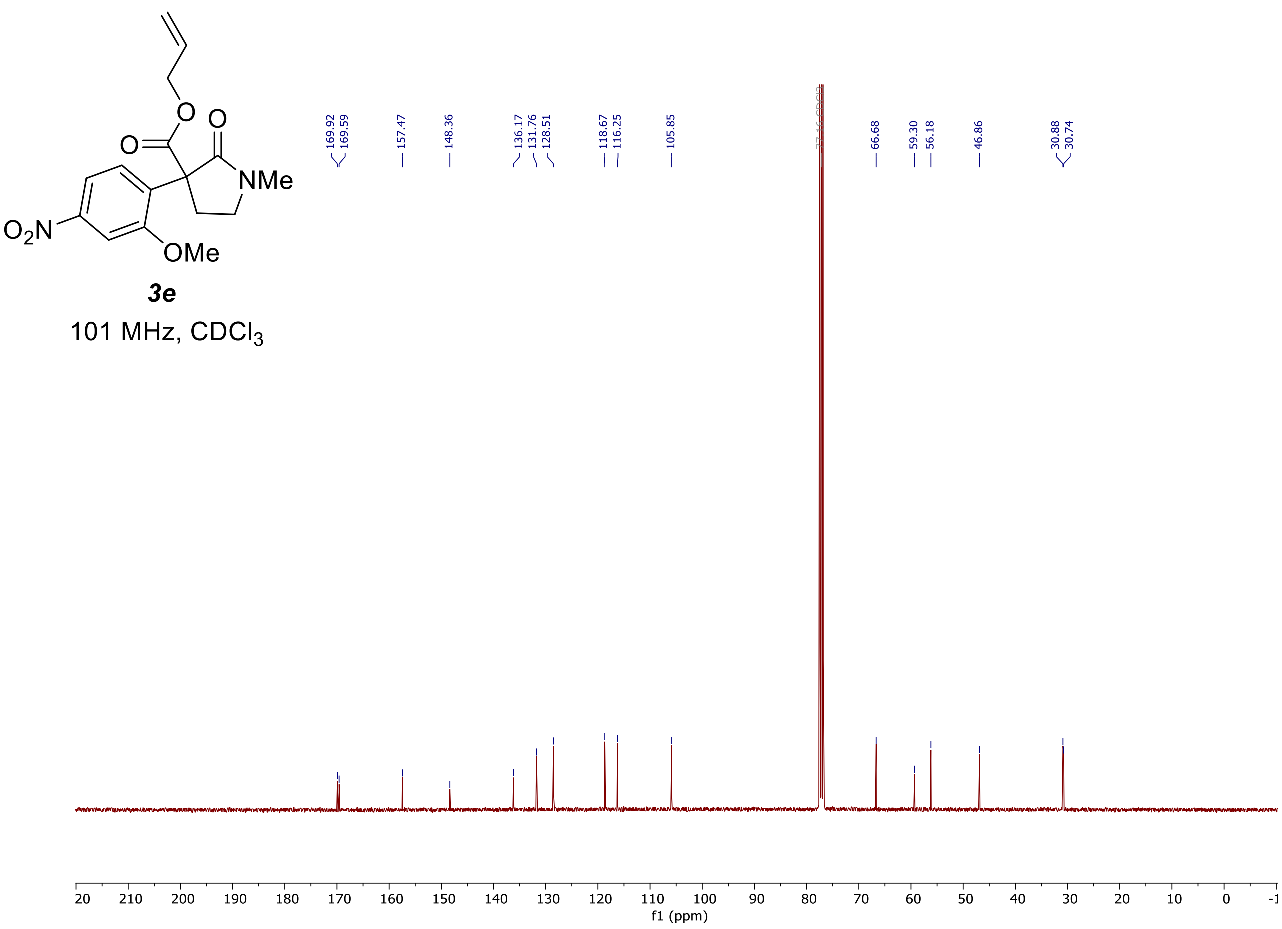
3e
101 MHz, CDCl3
169.92
169.59
157.47
148.36
136.17
131.76
128.51
118.67
116.25
105.85
66.68
59.30
56.18
46.86
30.88
30.74
f1 (ppm)

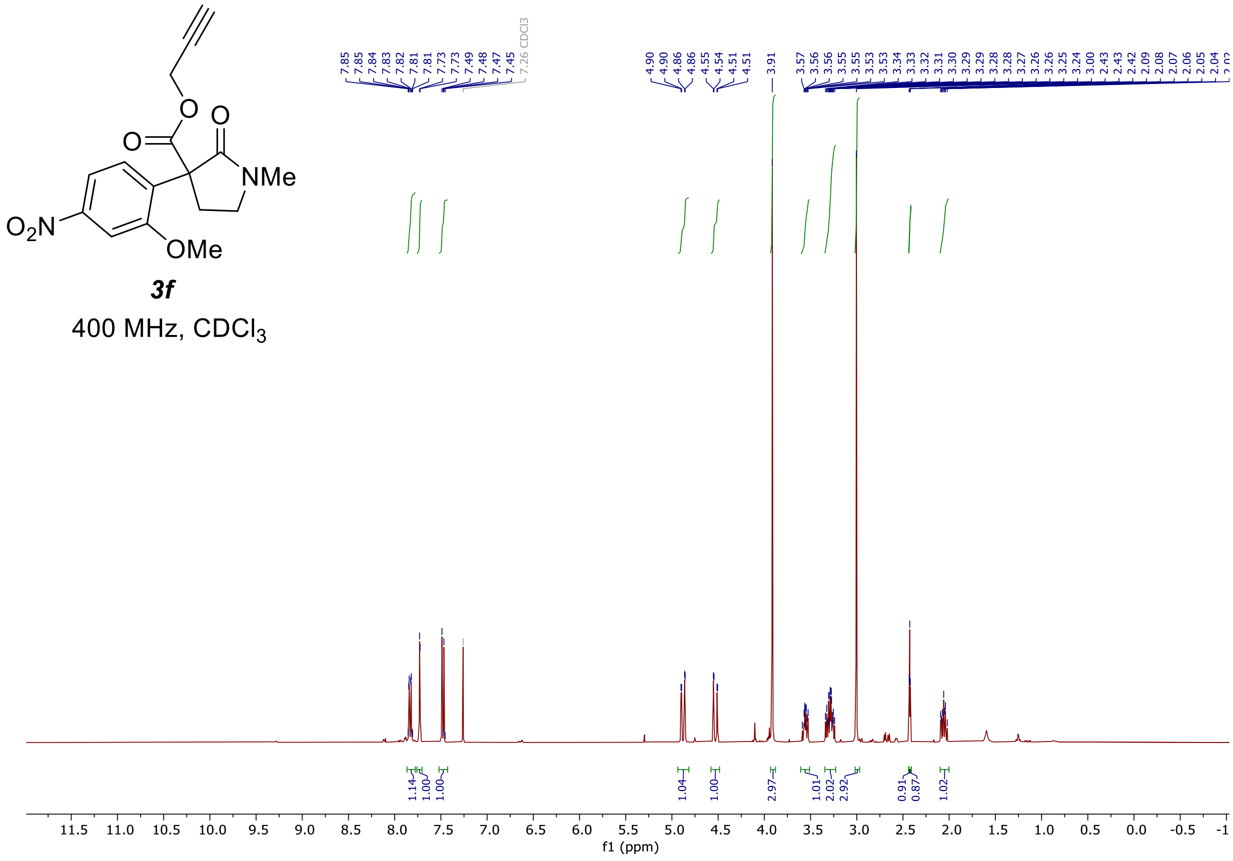
3f
400 MHz, CDCl3
7.26 CDCl3
3.91
f1 (ppm)

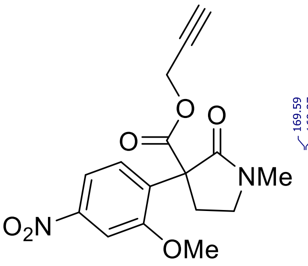

3f

101 MHz, $CDCl_3$

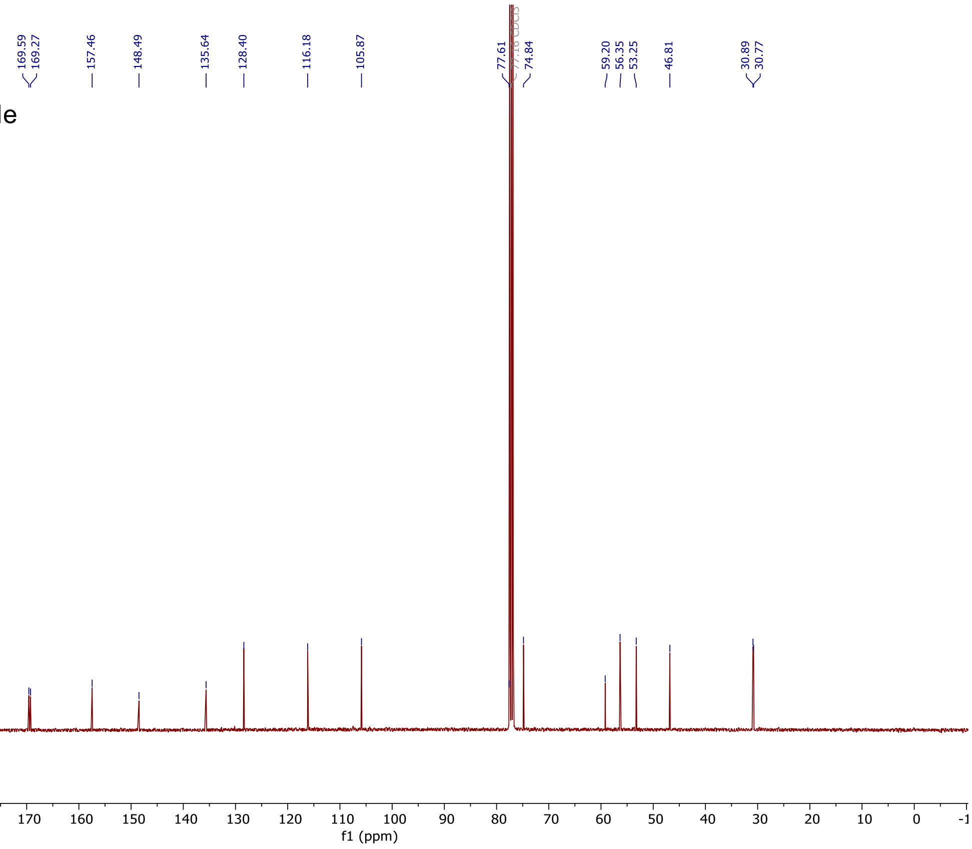

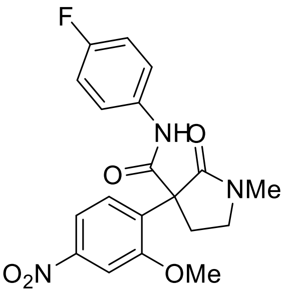

3g

400 MHz, $CDCl_3$

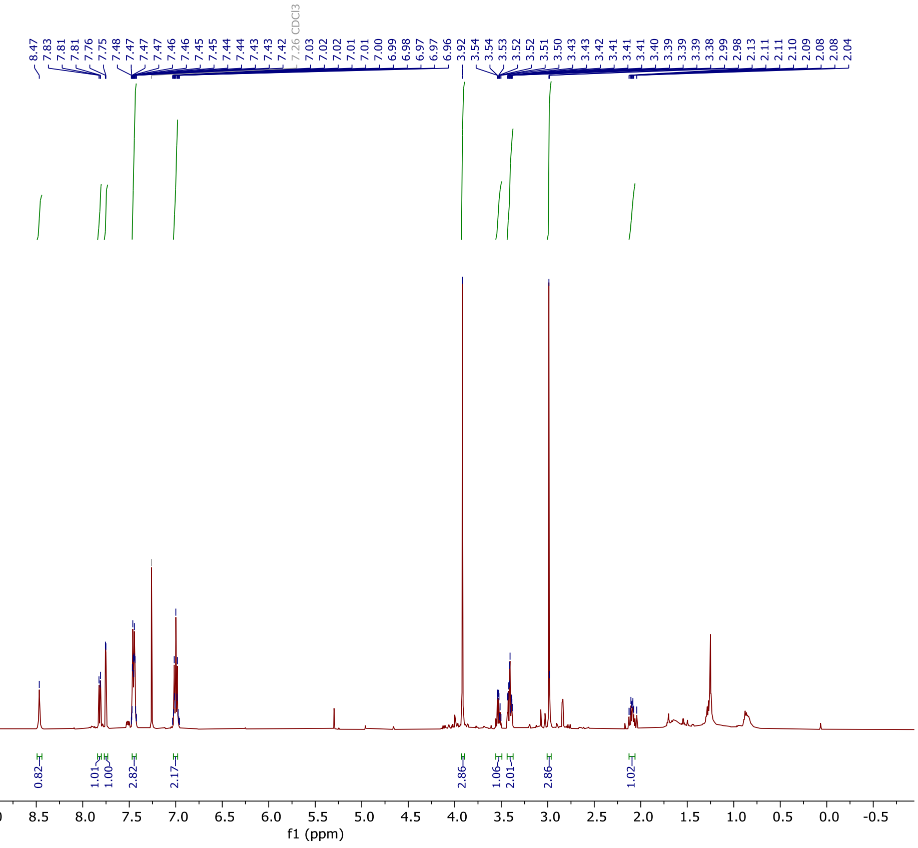

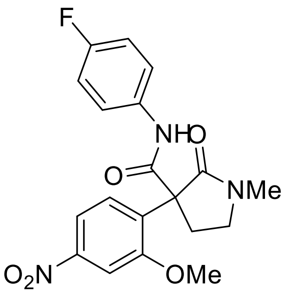

3g

101 MHz, CDCl$_3$

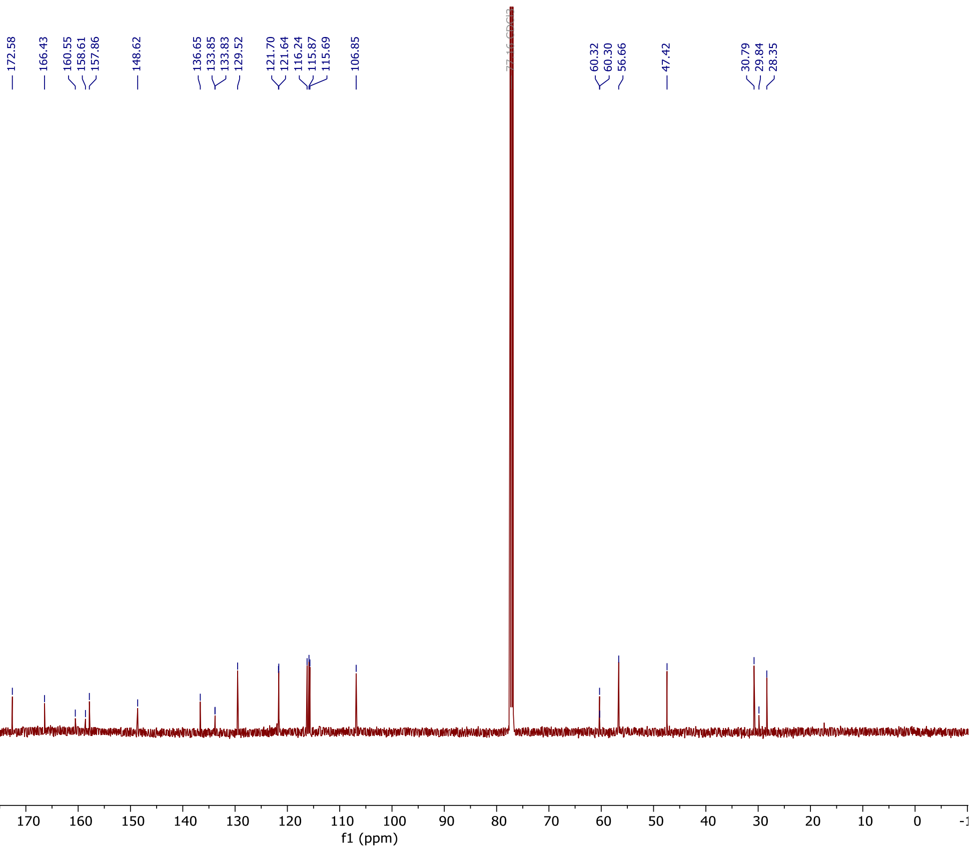

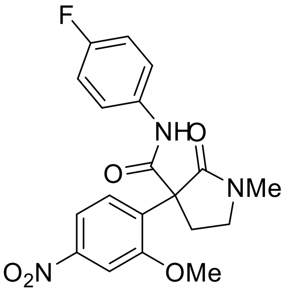

3g

101 MHz, CDCl$_3$

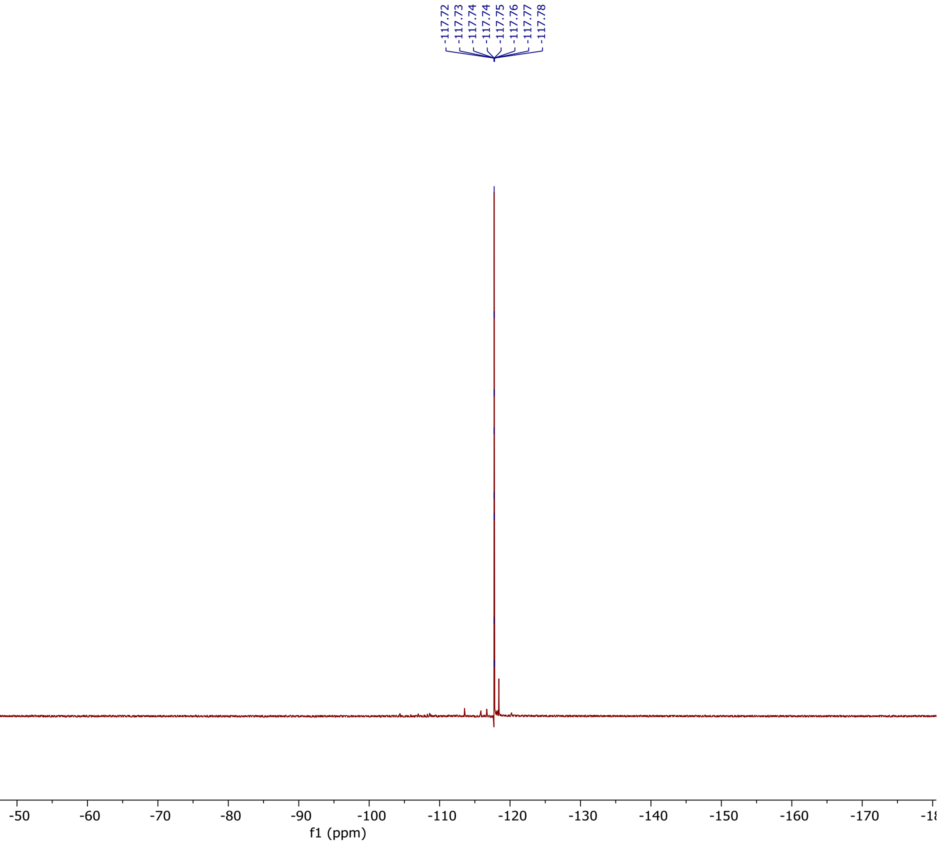

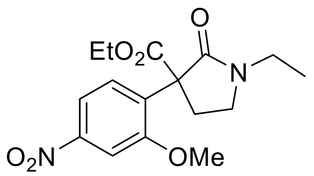

3h

400 MHz, $CDCl_3$

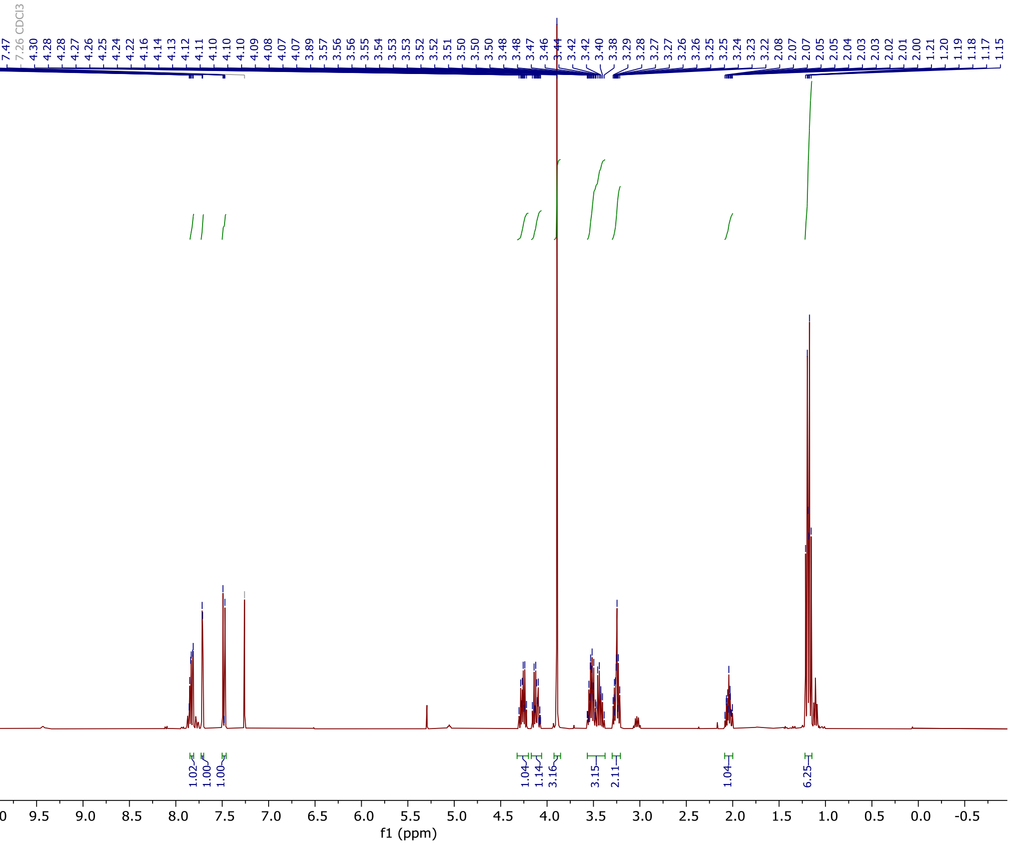

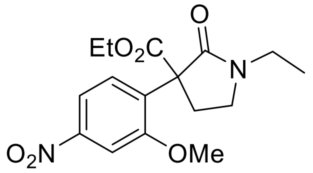

3h

101 MHz, $CDCl_3$

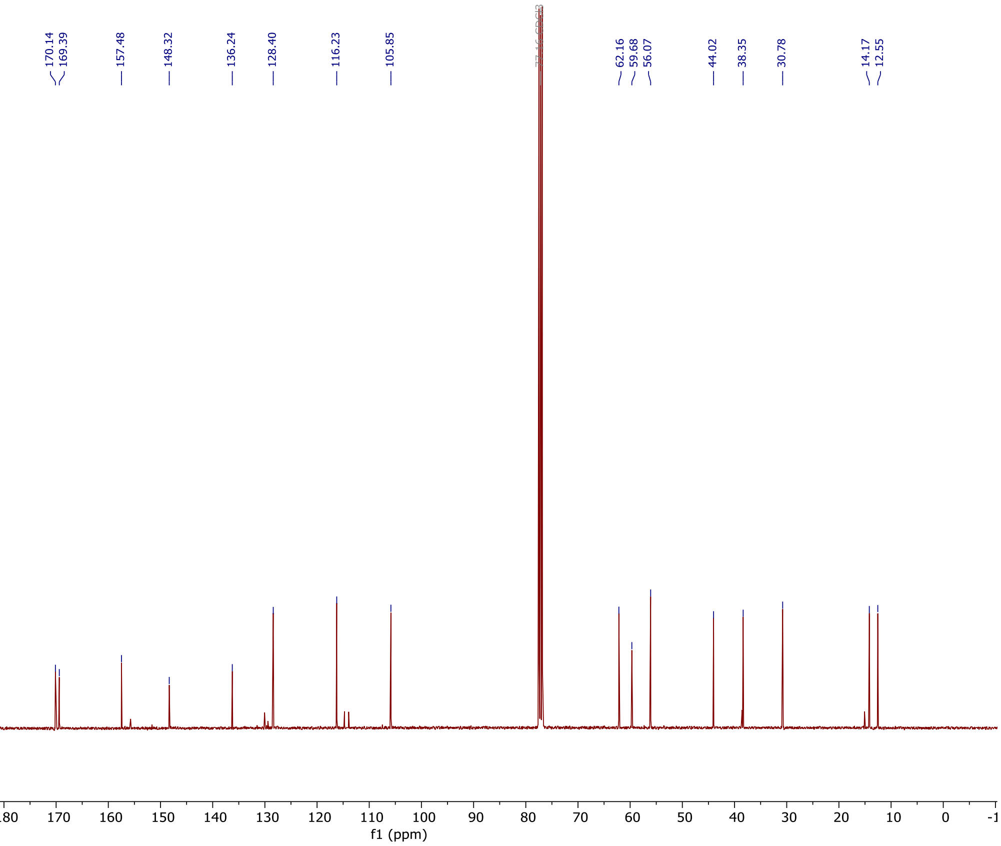

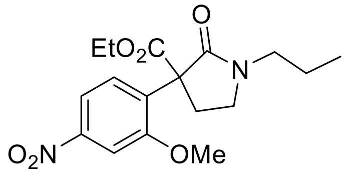

3i

400 MHz, $CDCl_3$

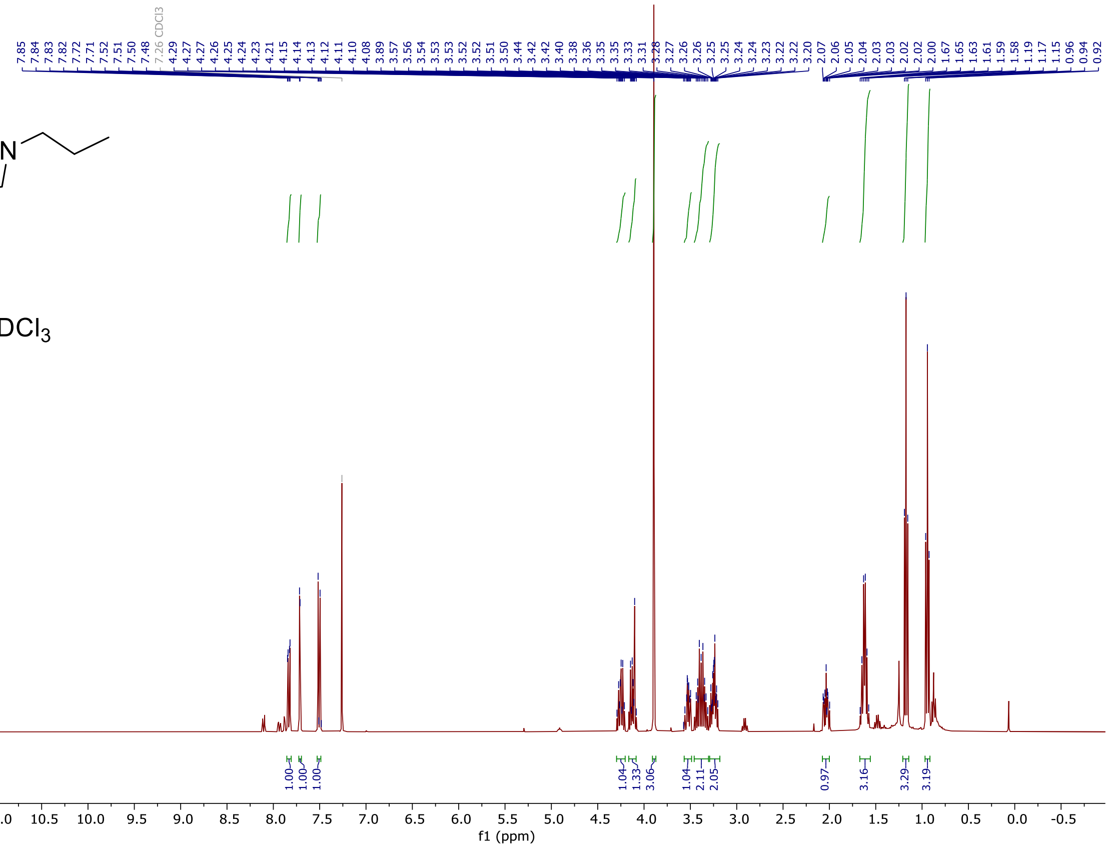

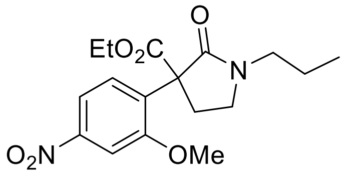

3i

101 MHz, $CDCl_3$

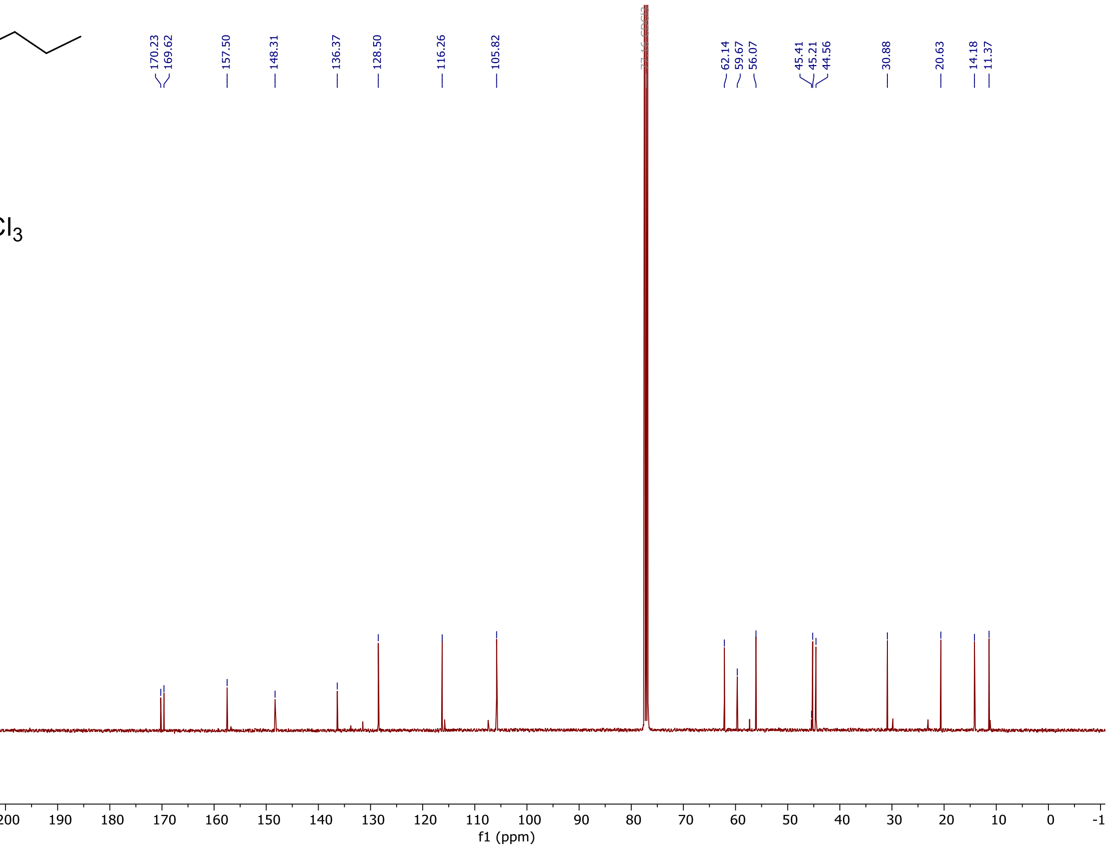

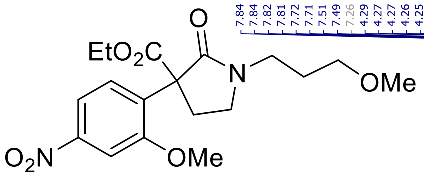

3j

400 MHz, $CDCl_3$

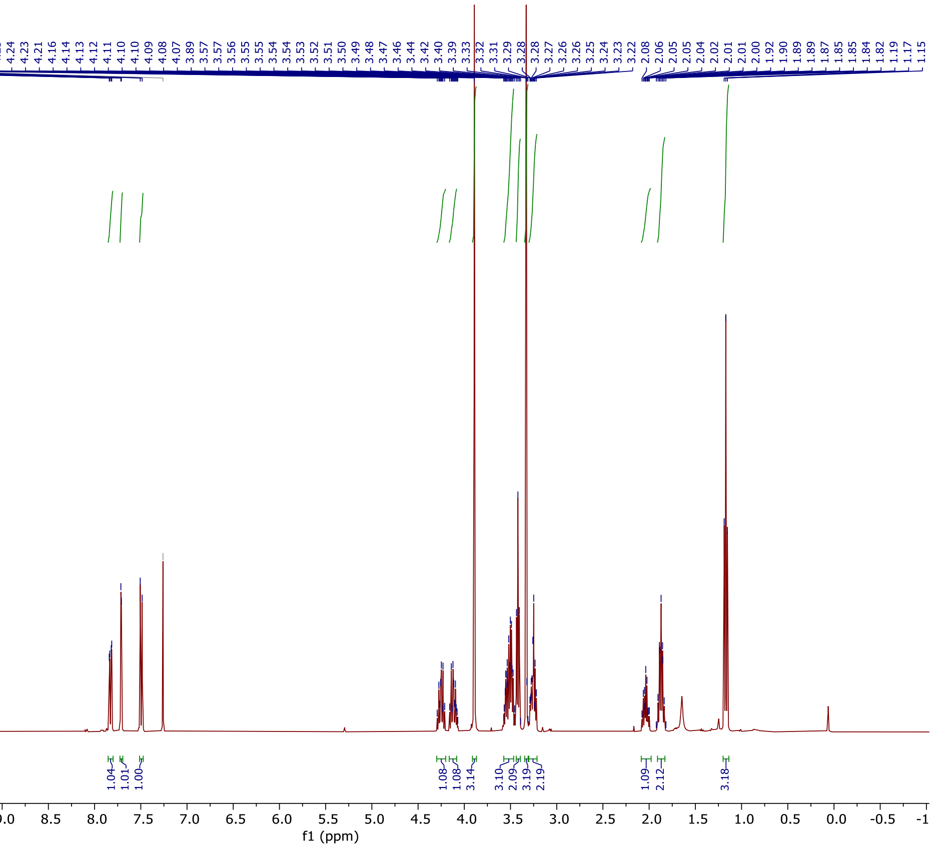

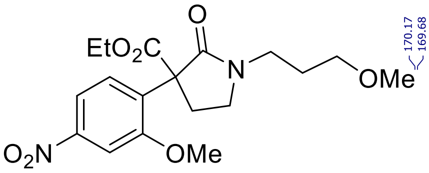

3j

101 MHz, $CDCl_3$

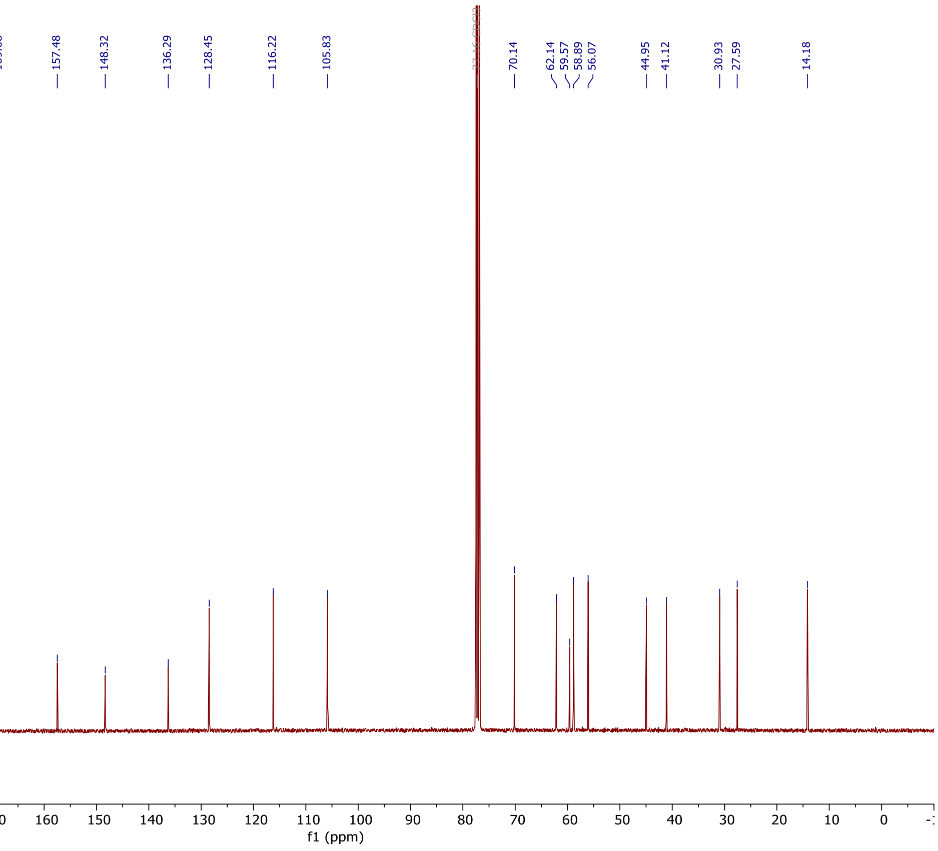

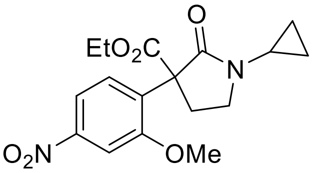

3k

400 MHz, $CDCl_3$

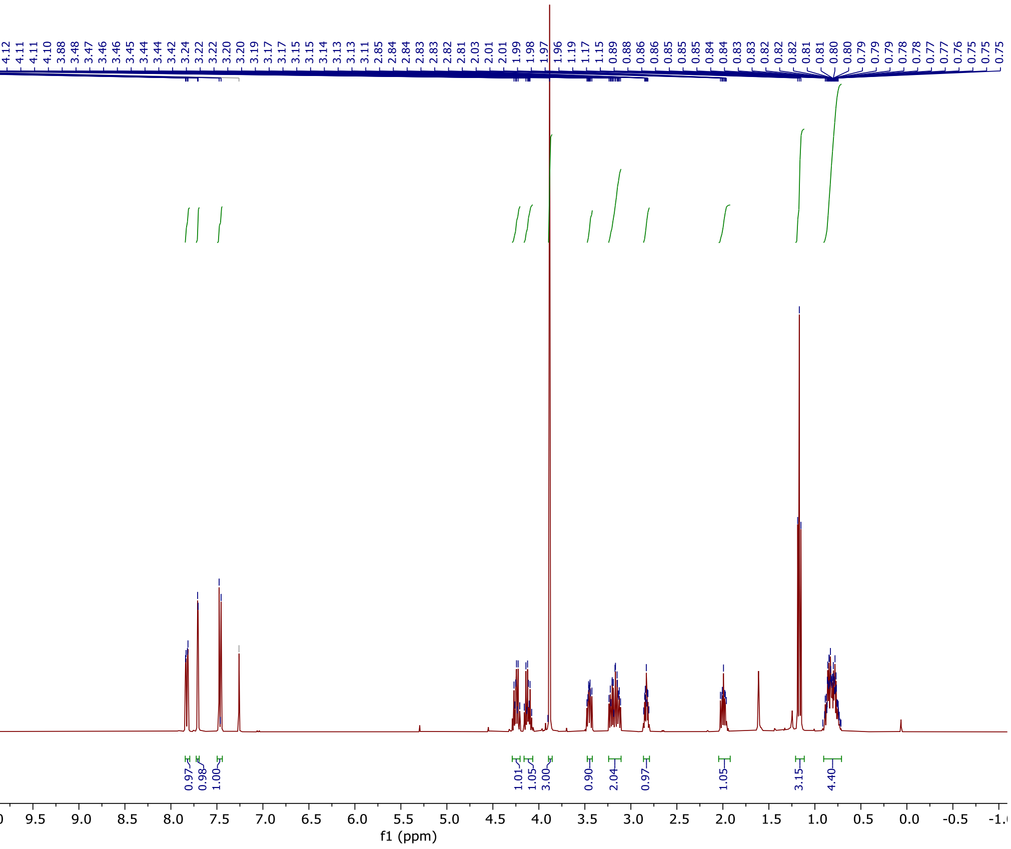

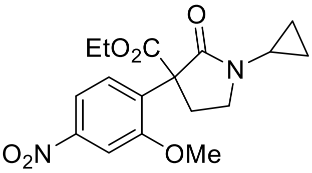

3k

101 MHz, $CDCl_3$

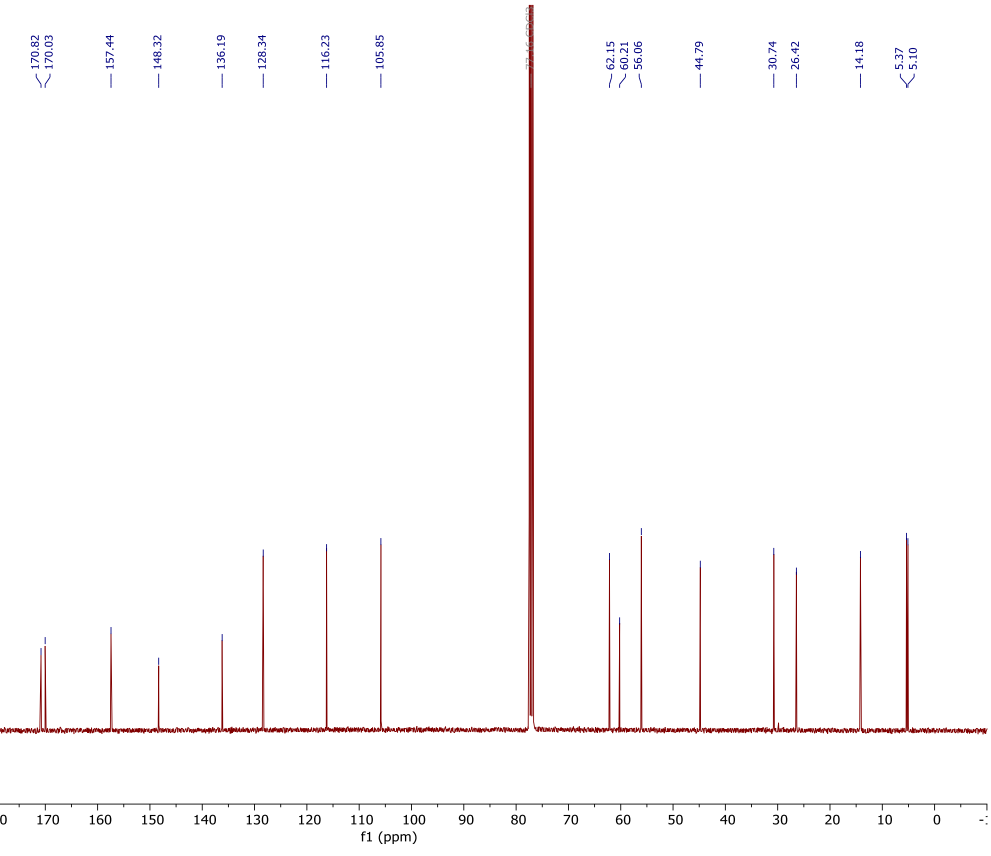

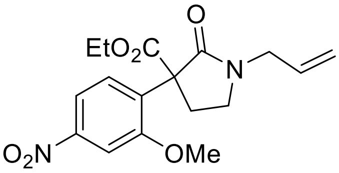

3l

400 MHz, $CDCl_3$

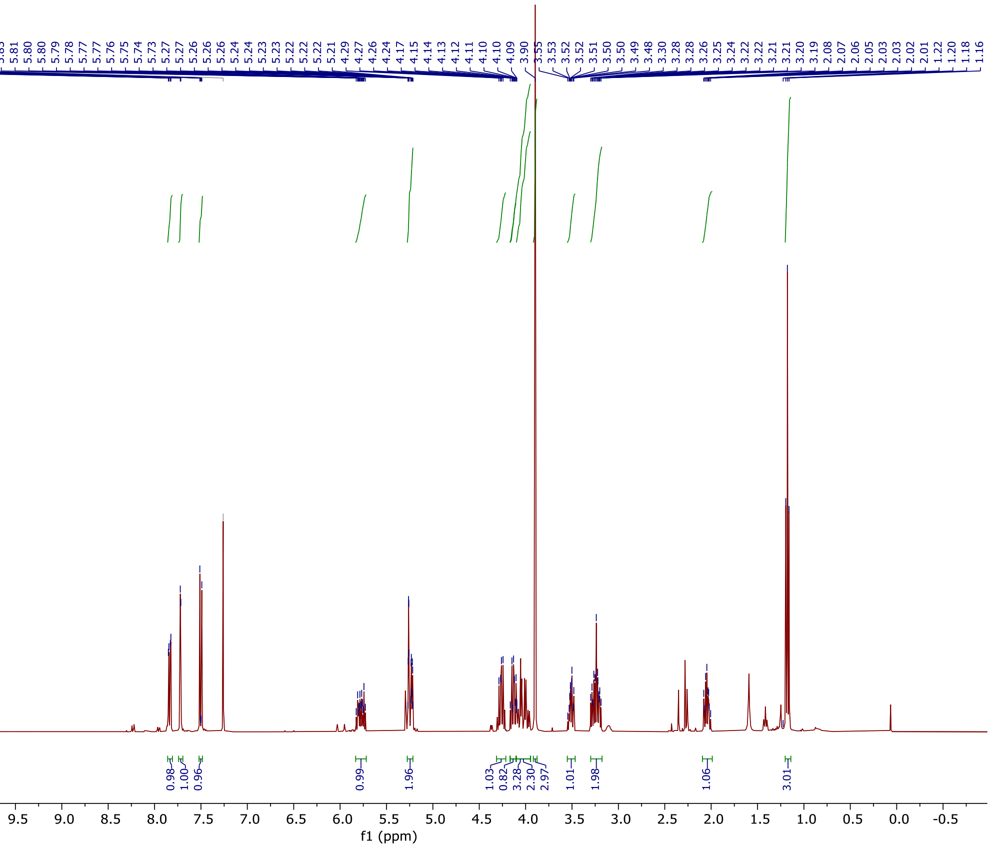

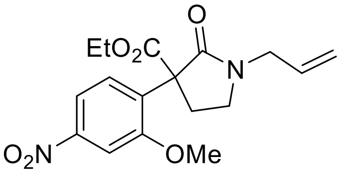

3l

101 MHz, $CDCl_3$

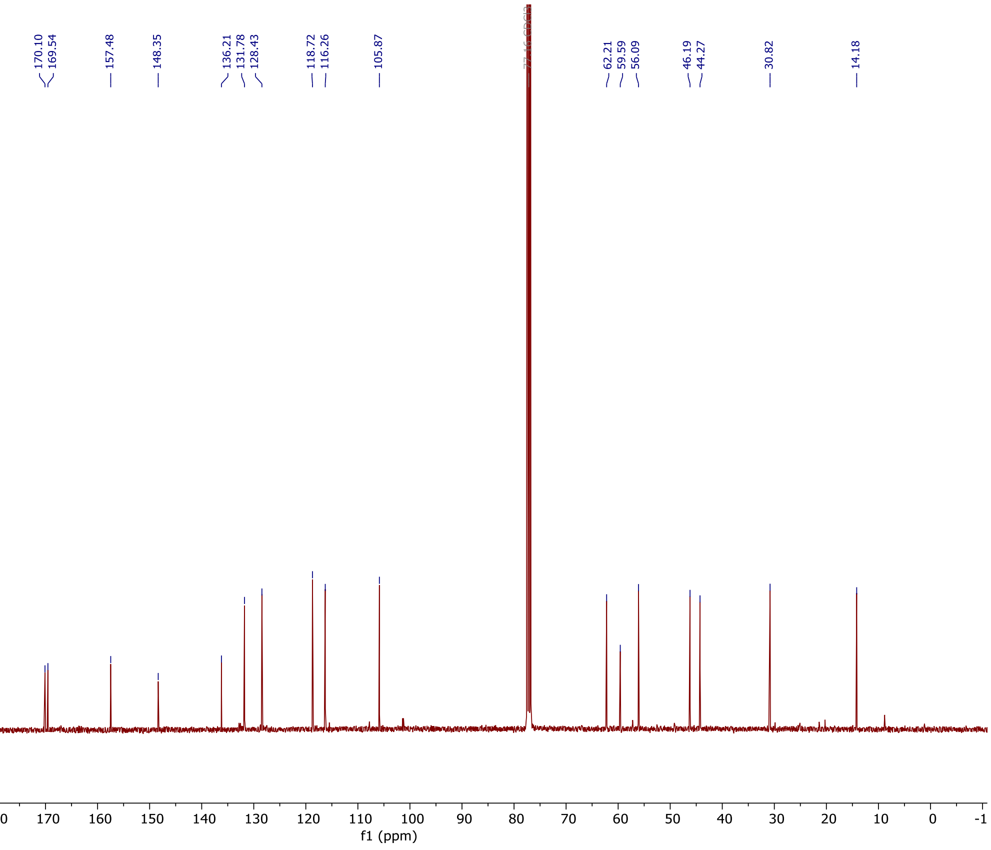

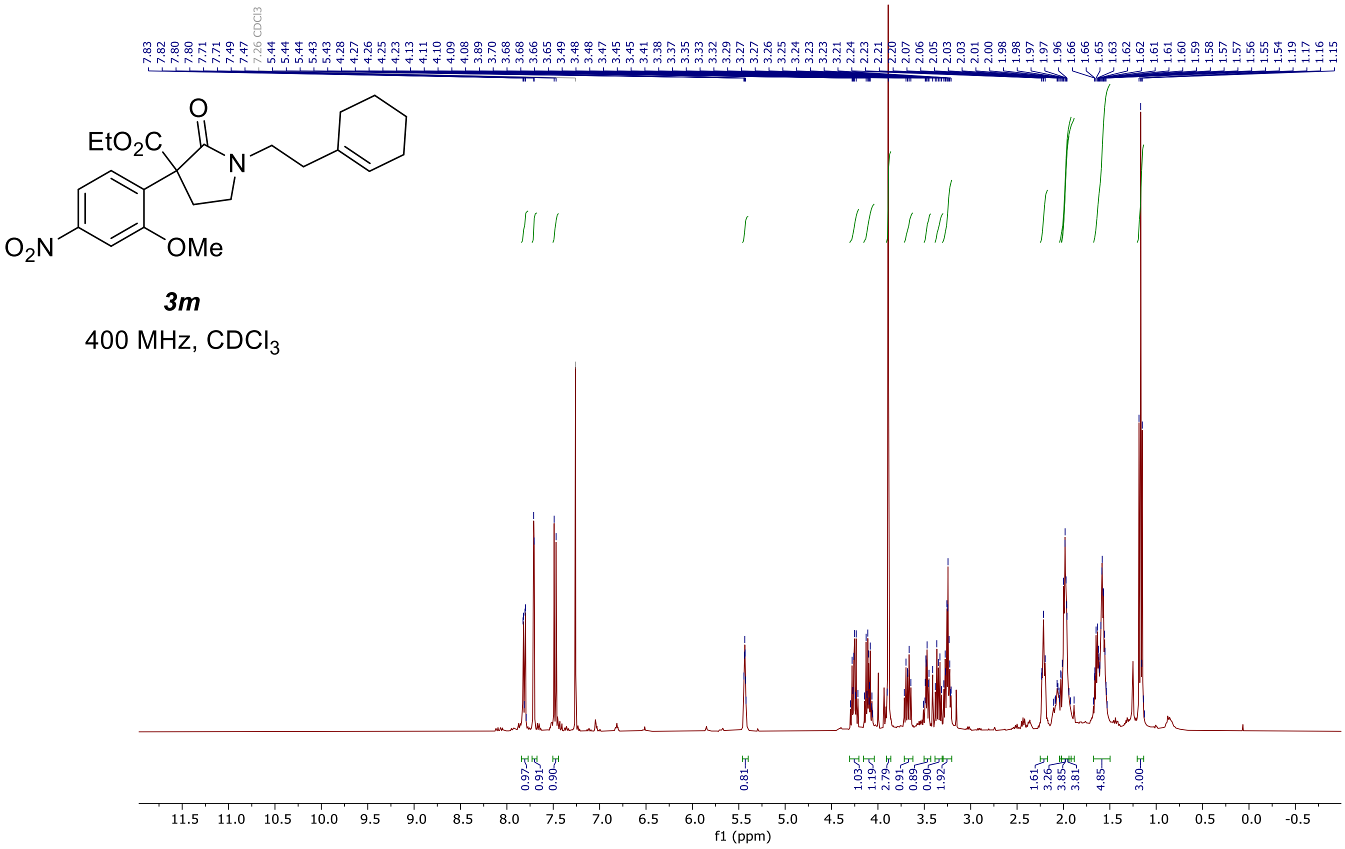

3m

400 MHz, $CDCl_3$

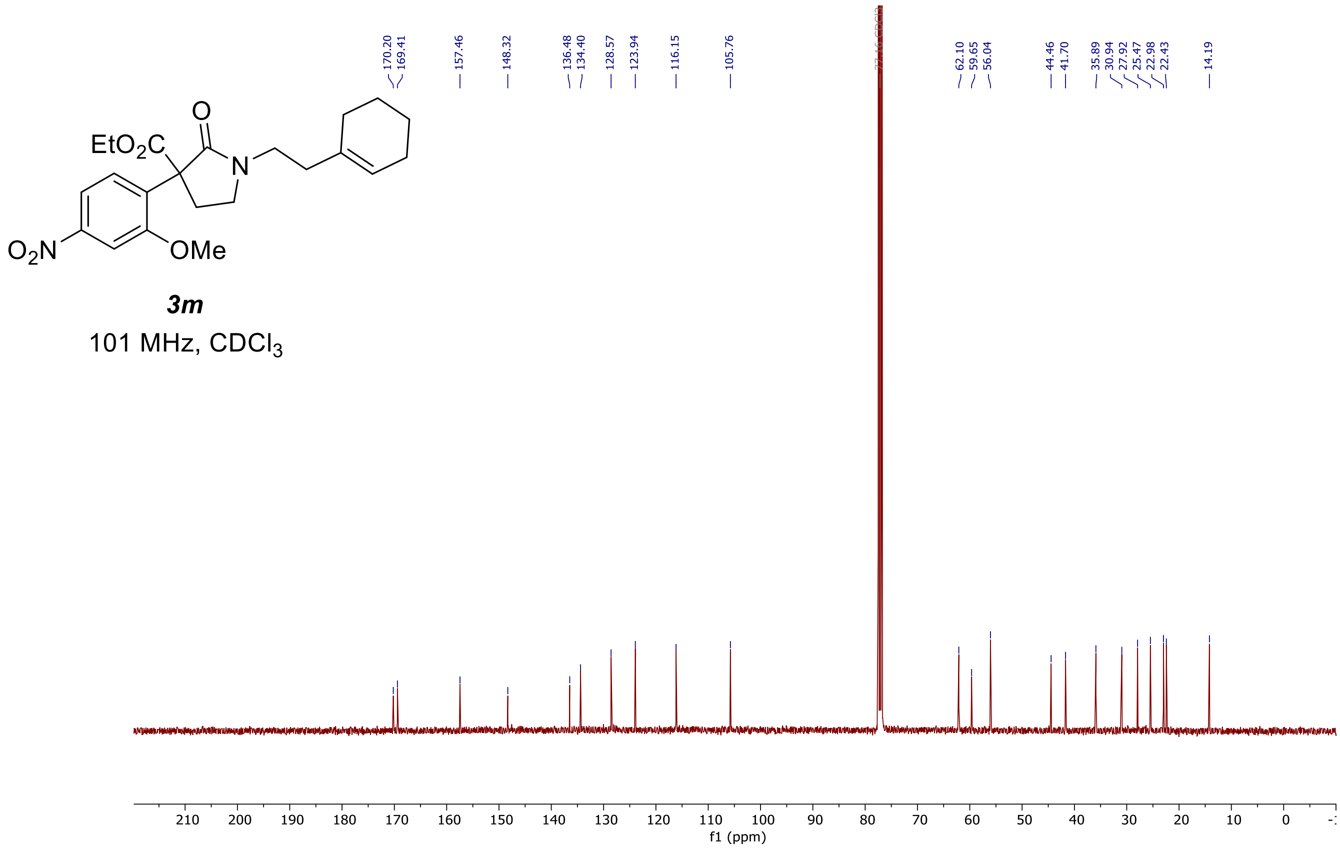

3m

101 MHz, $CDCl_3$

3n

400 MHz, $CDCl_3$

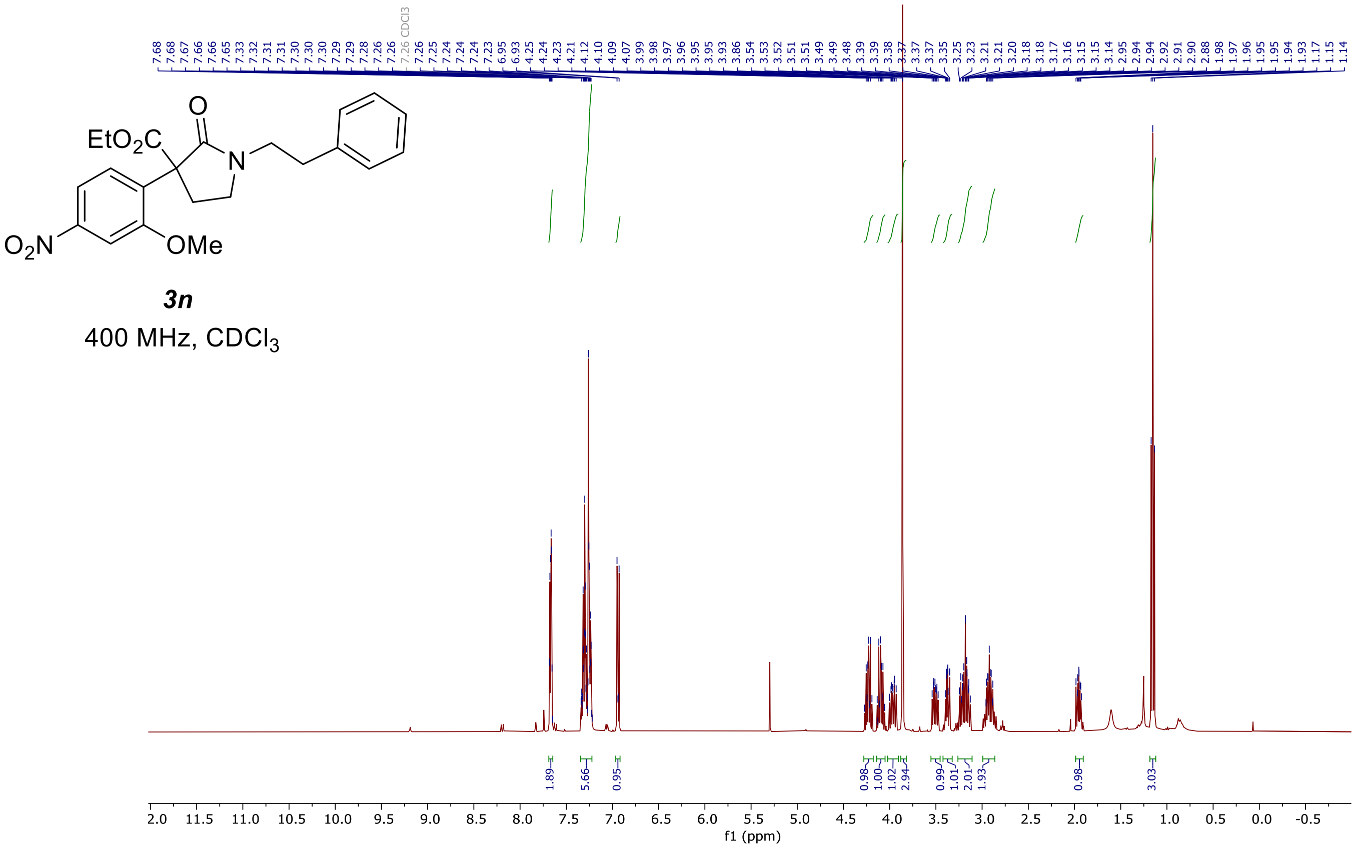

3n

101 MHz, $CDCl_3$

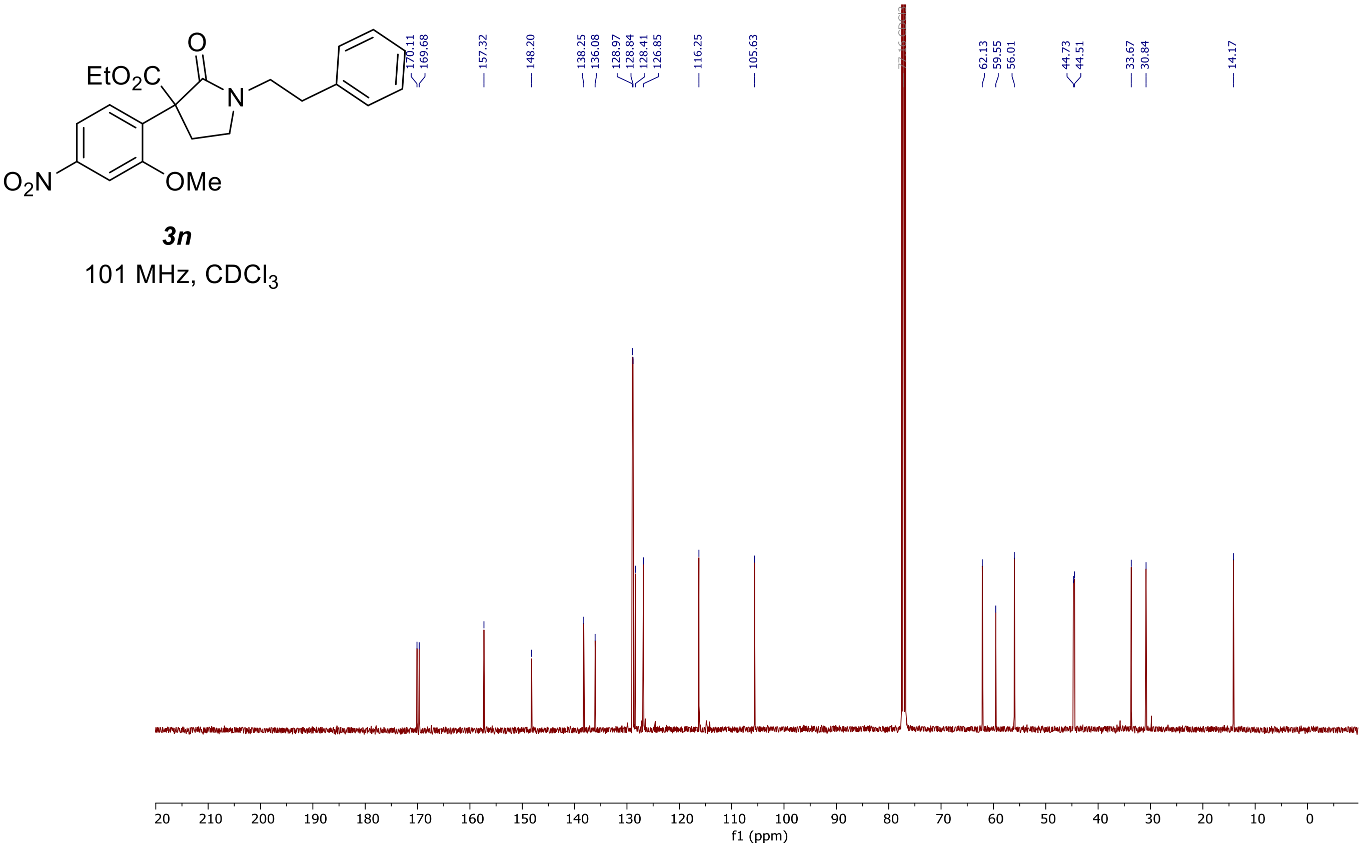

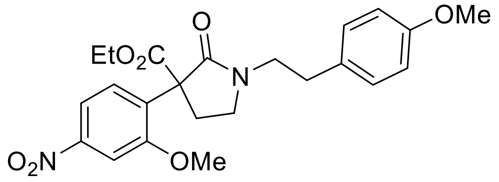

3o

400 MHz, $CDCl_3$

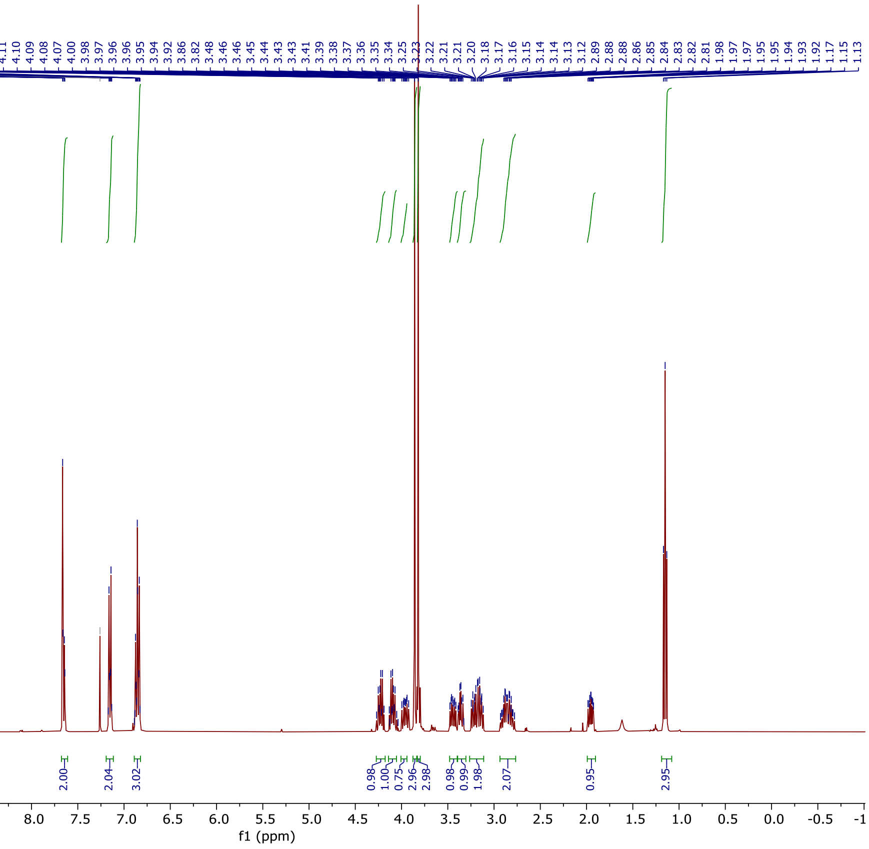

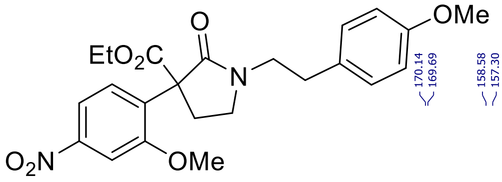

3o

101 MHz, $CDCl_3$

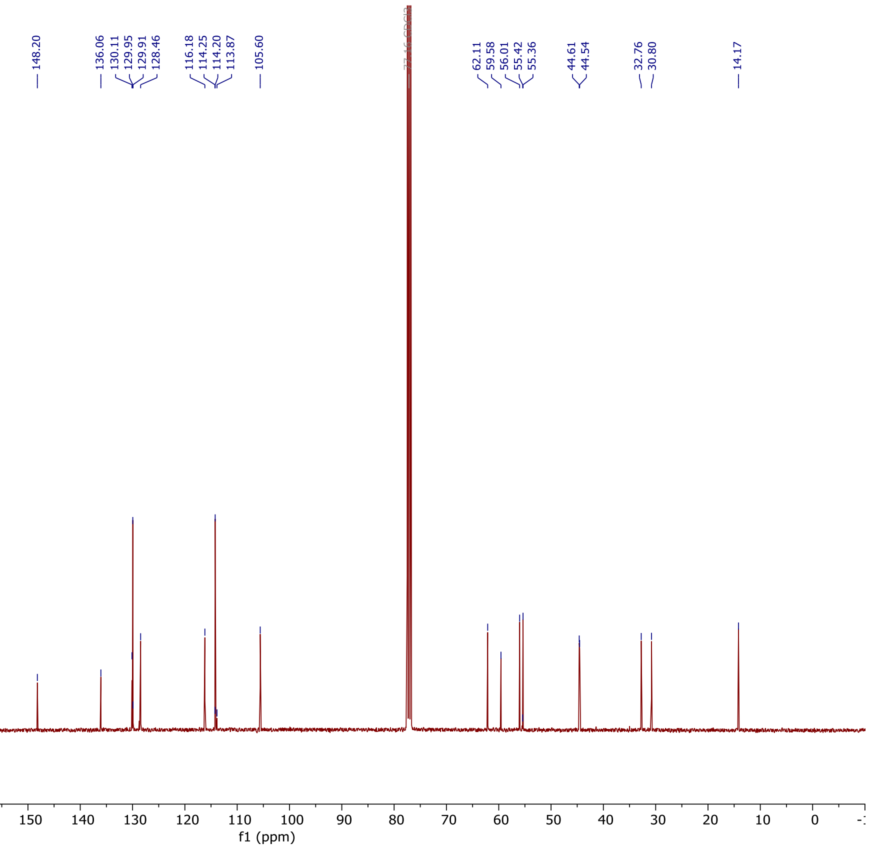

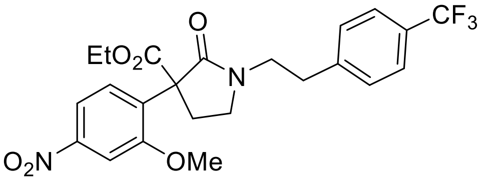

3p

400 MHz, $CDCl_3$

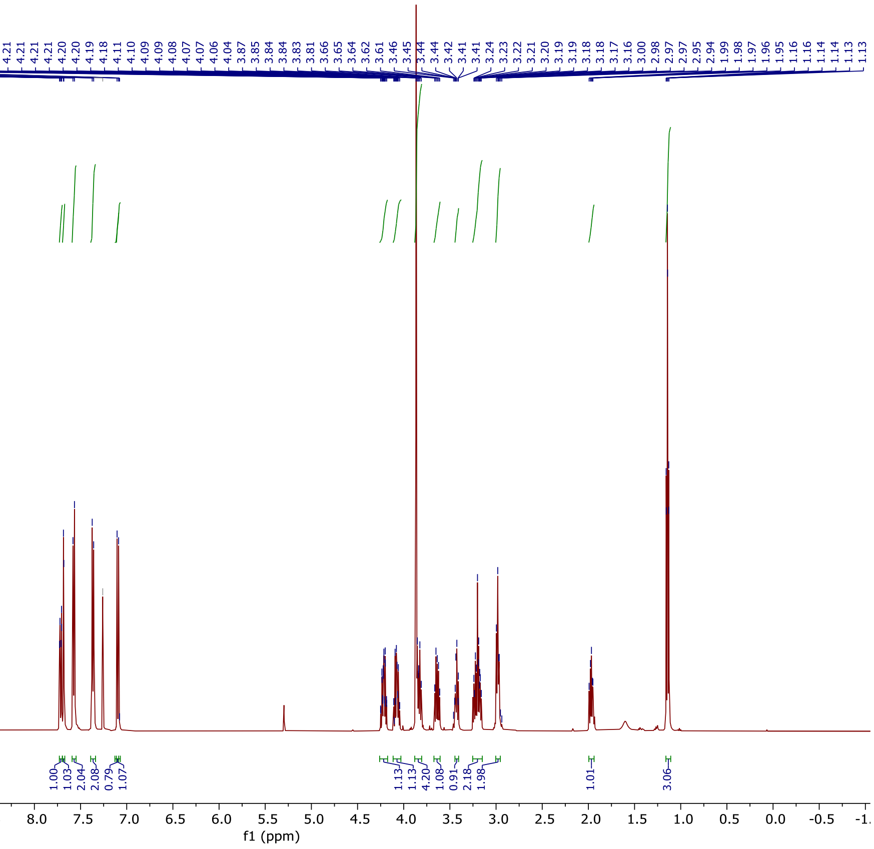

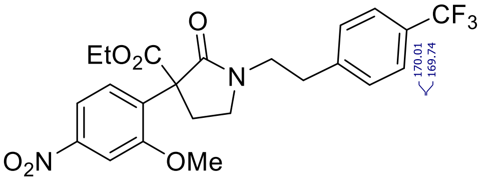

3p

101 MHz, $CDCl_3$

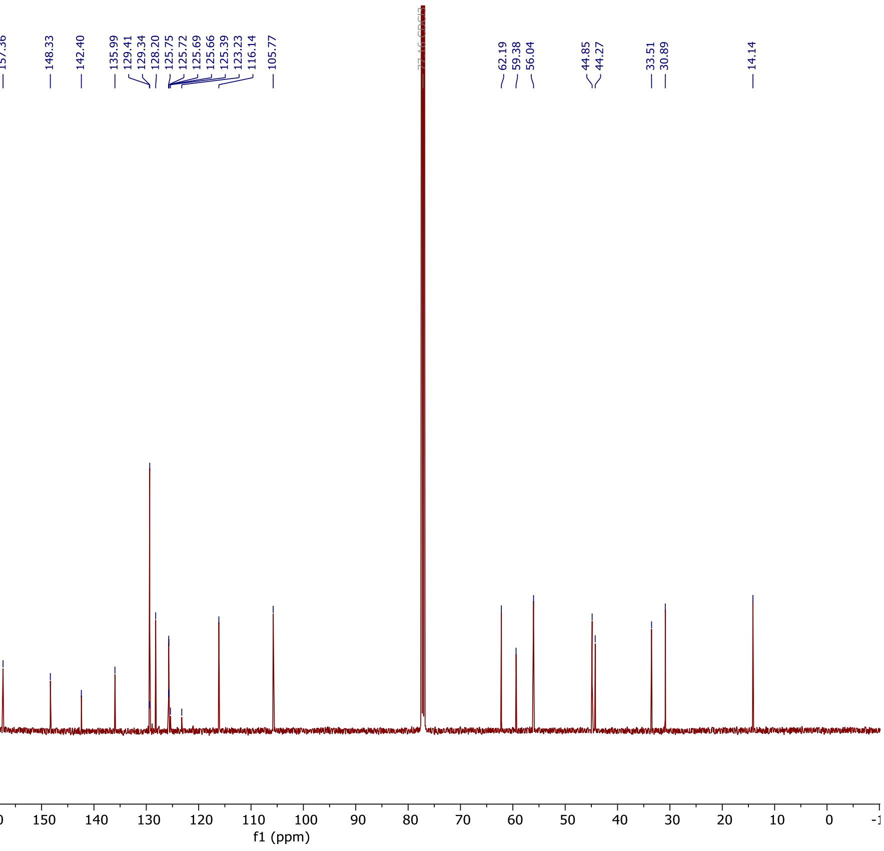

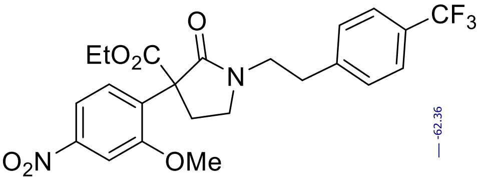

3p

101 MHz, $CDCl_3$

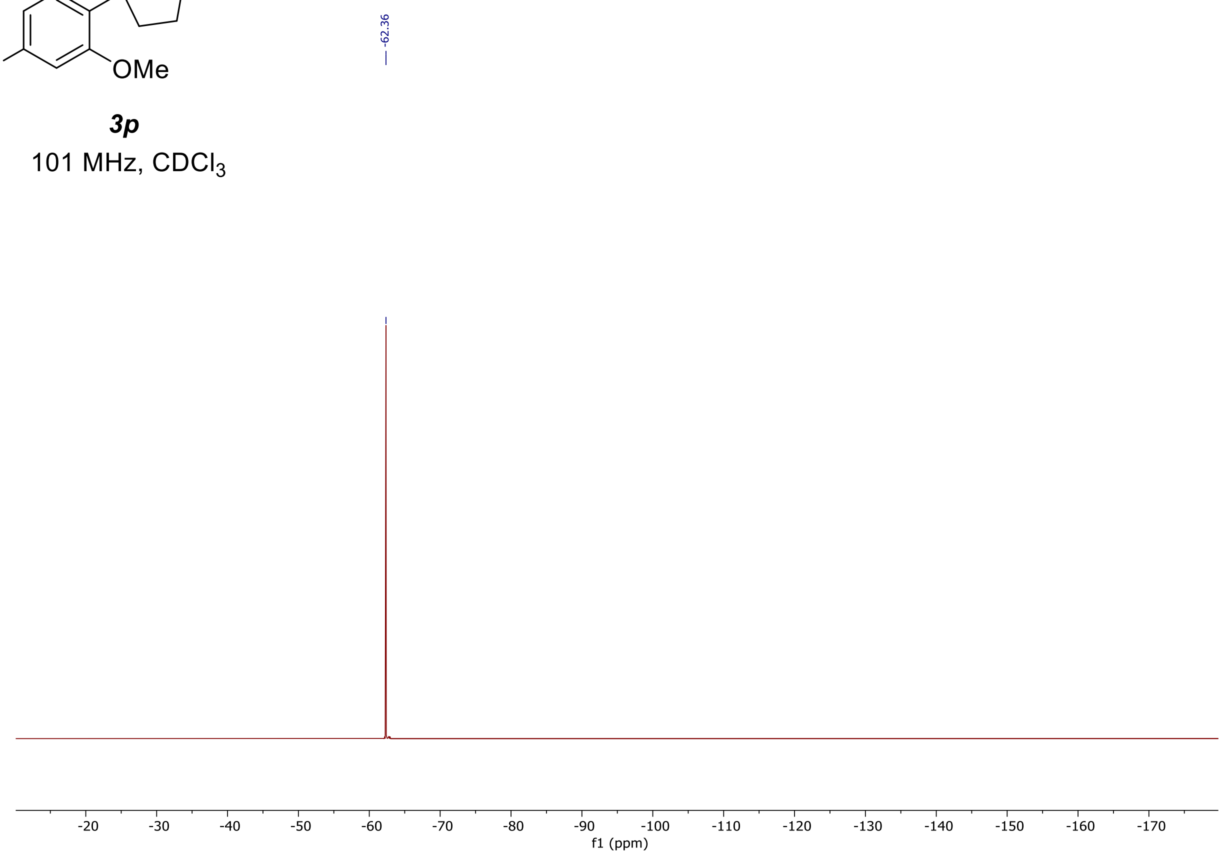

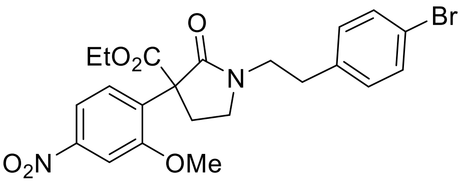

3q

500 MHz, $CDCl_3$

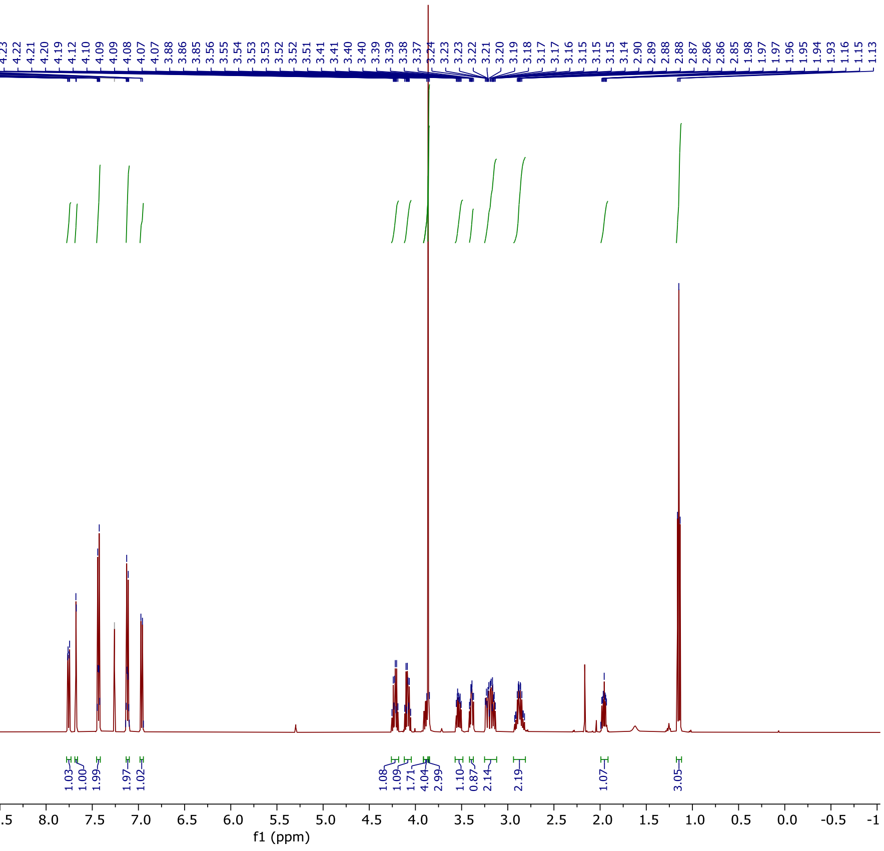

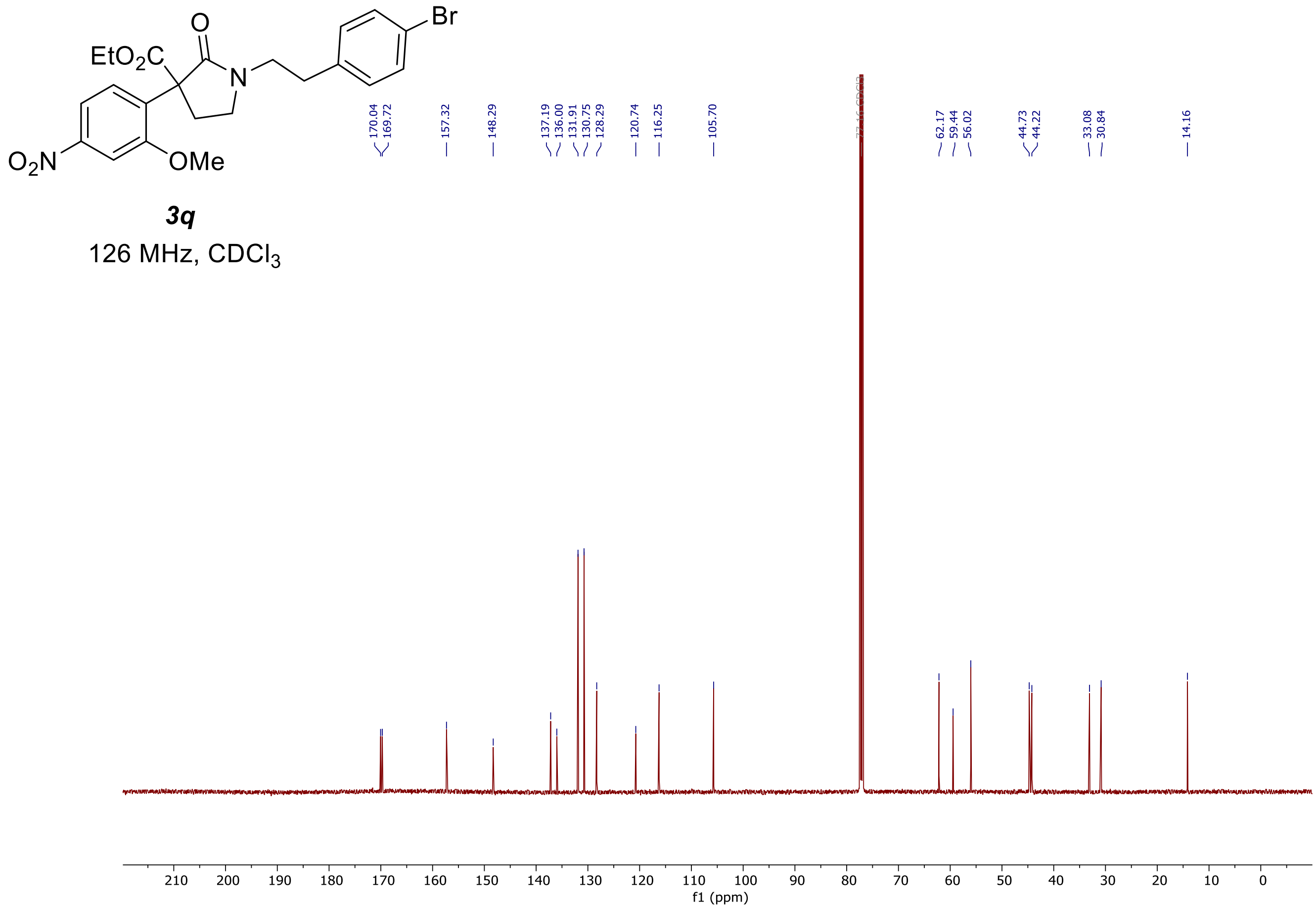
EtO2C
O
N
Br
O2N
OMe
3q
126 MHz, CDCl3
170.04
169.72
157.32
148.29
137.19
136.00
131.91
130.75
128.29
120.74
116.25
105.70
62.17
59.44
56.02
44.73
44.22
33.08
30.84
14.16
f1 (ppm)

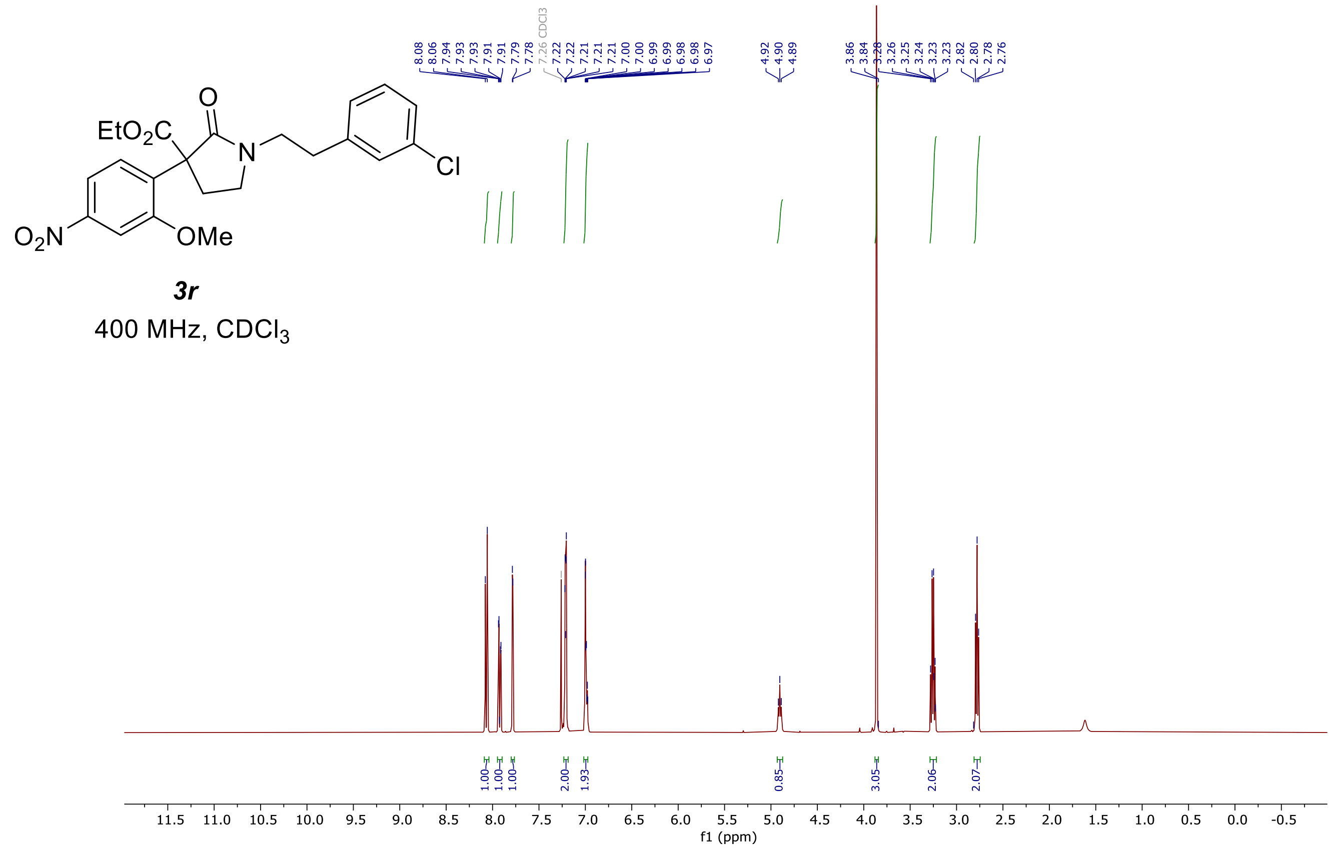
EtO2C
O
N
Cl
O2N
OMe
3r
400 MHz, CDCl3
8.08
8.06
7.94
7.93
7.93
7.91
7.91
7.79
7.78
7.26 CDCl3
7.22
7.22
7.21
7.21
7.21
7.00
6.99
6.99
6.98
6.98
6.97
4.92
4.90
4.89
3.86
3.84
3.28
3.26
3.25
3.24
3.23
3.23
2.82
2.80
2.78
2.76
1.00
1.00
1.00
2.00
1.93
0.85
3.05
2.06
2.07
f1 (ppm)

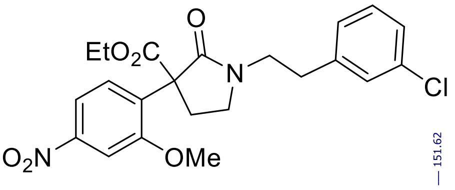

3r

101 MHz, $CDCl_3$

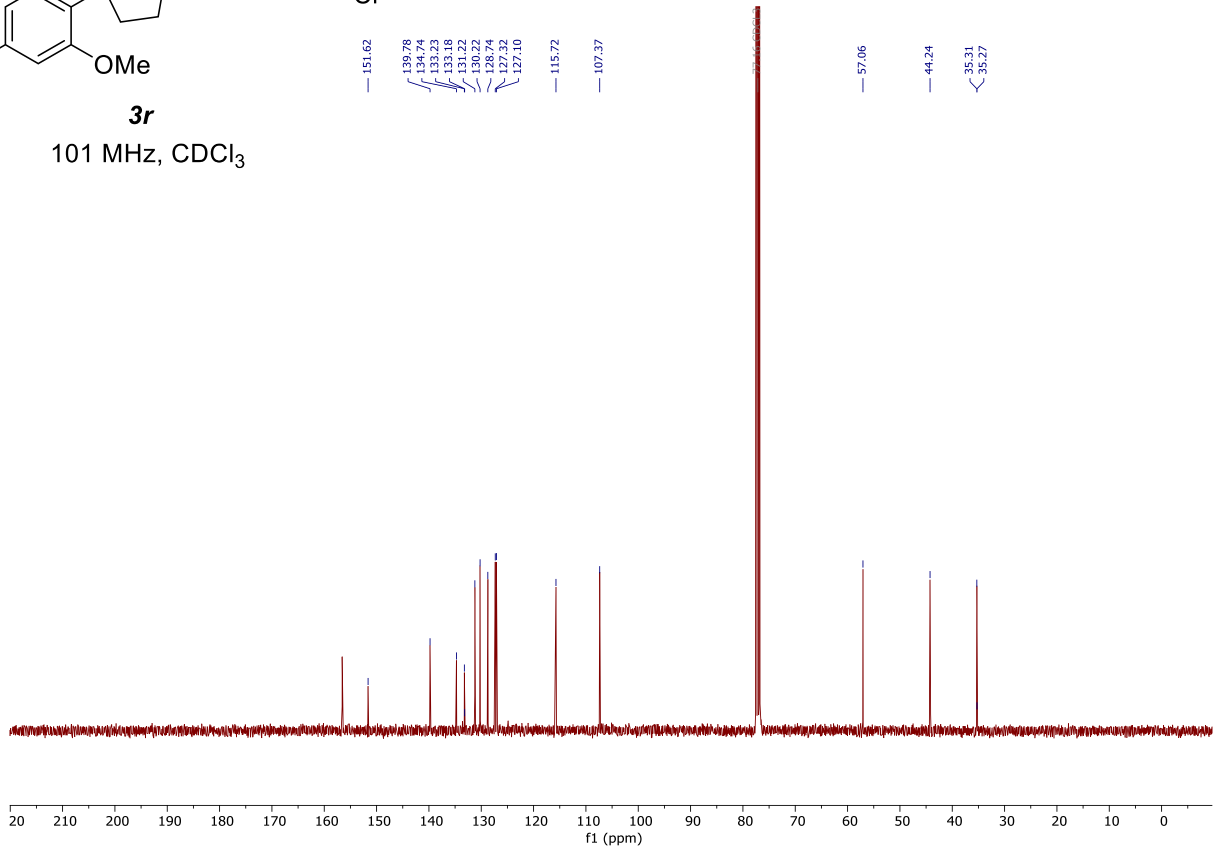

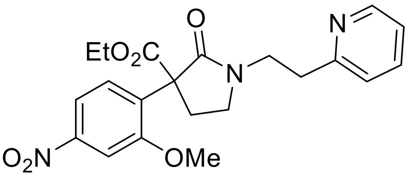

3s

400 MHz, $CDCl_3$

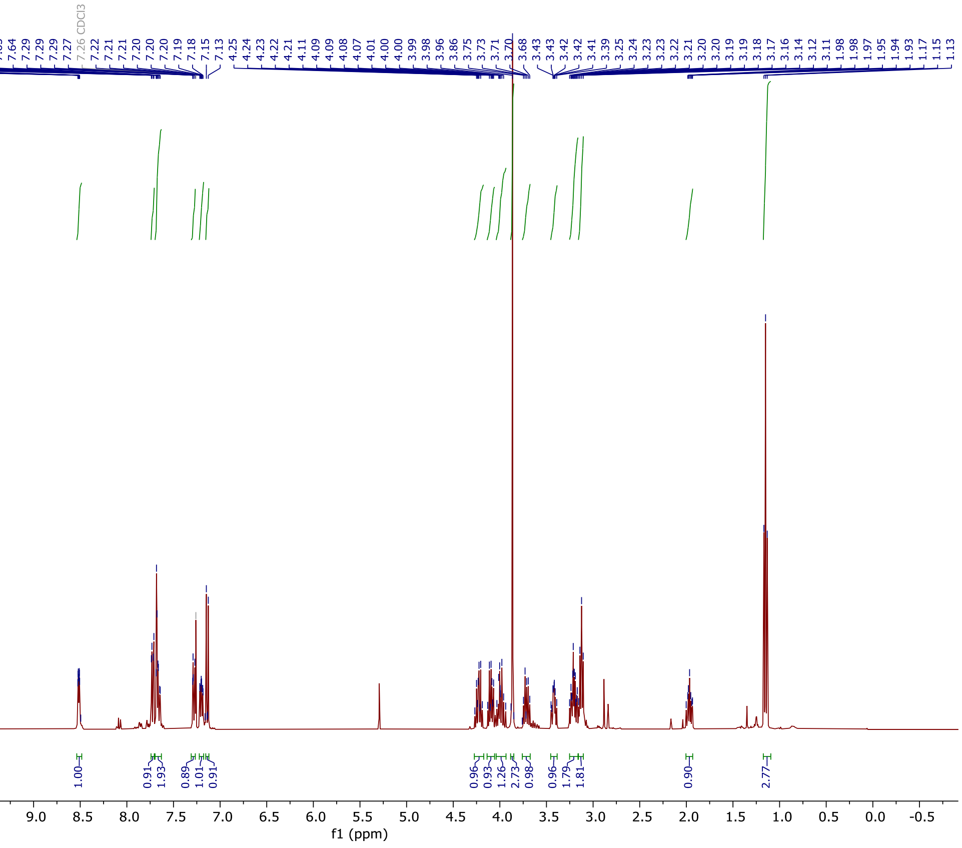

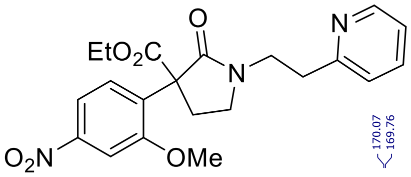

3s

101 MHz, $CDCl_3$

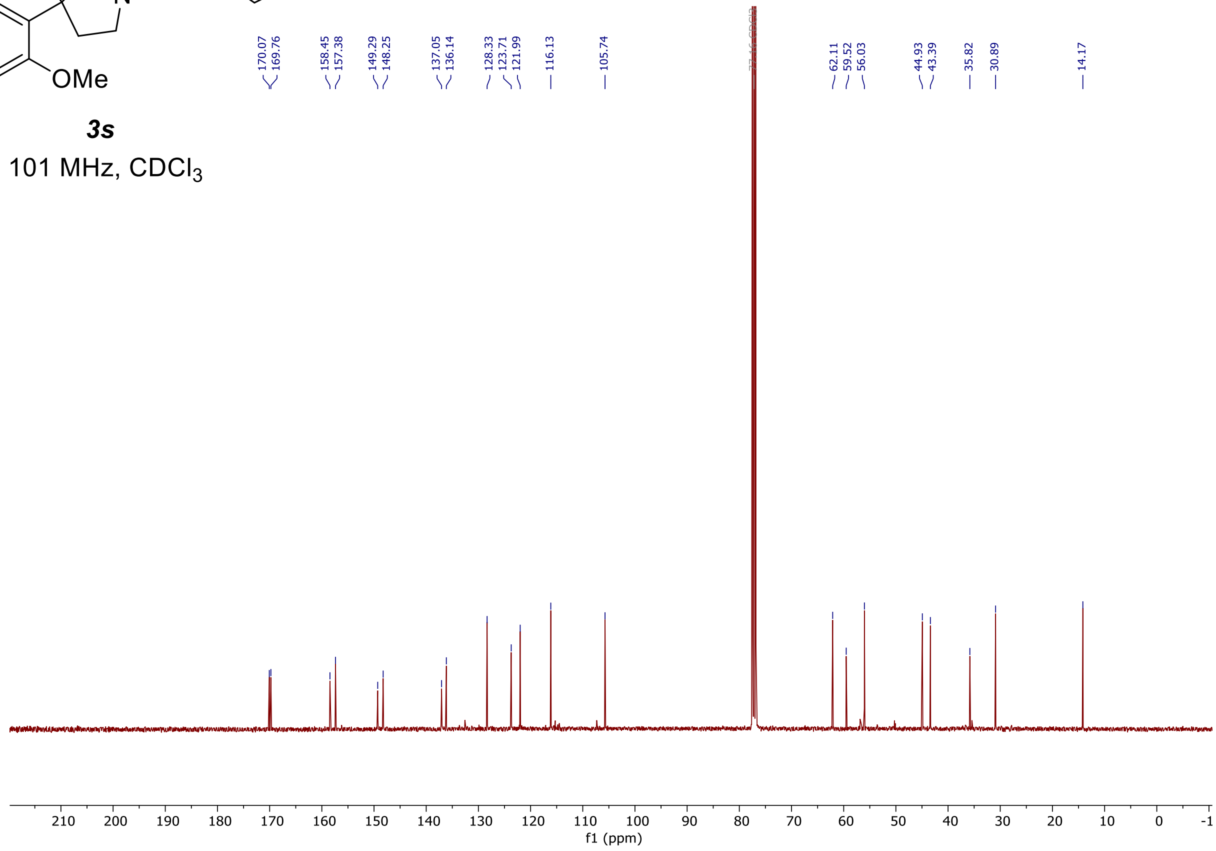

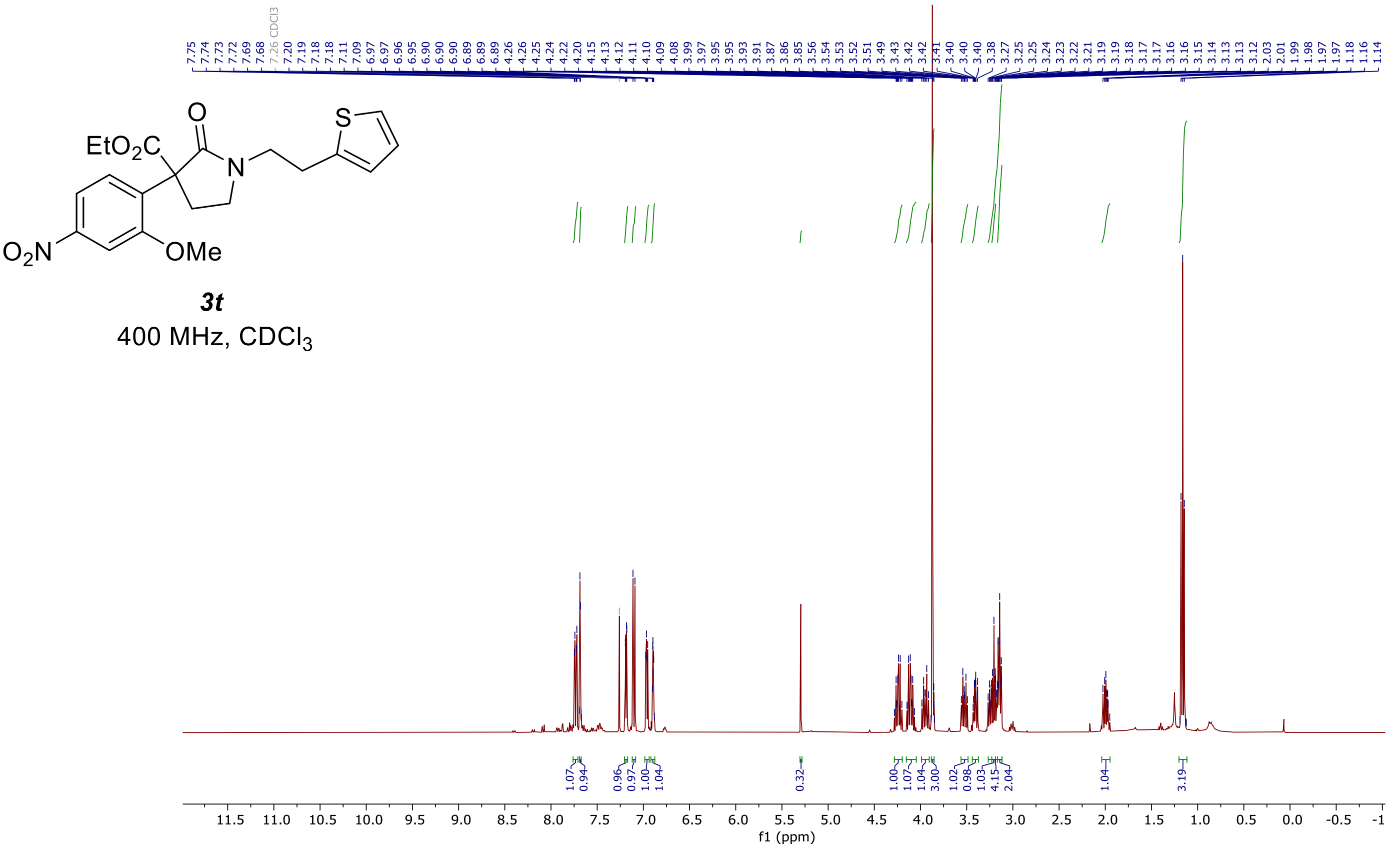

3t

400 MHz, $CDCl_3$

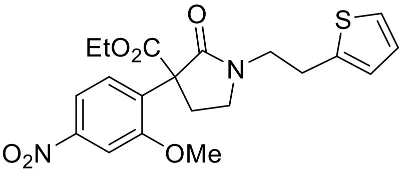

3t

101 MHz, $CDCl_3$

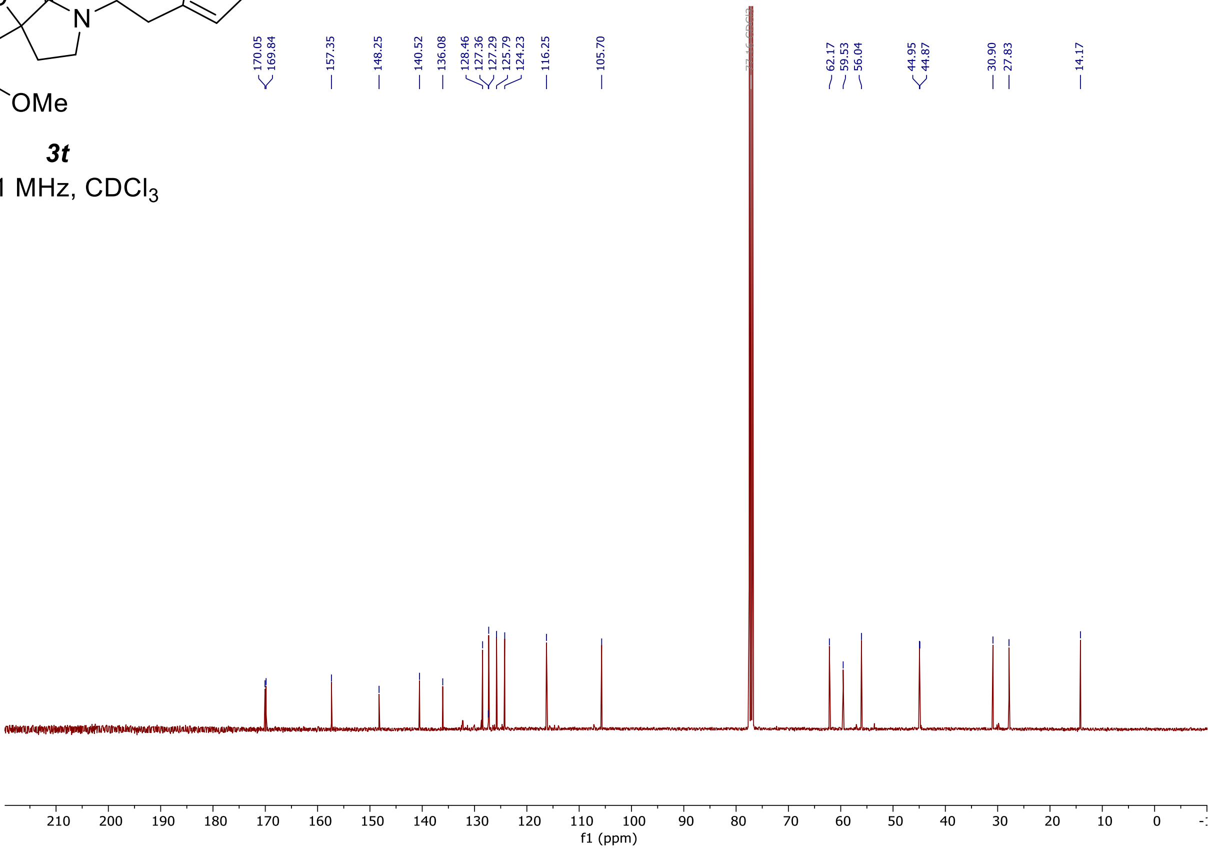

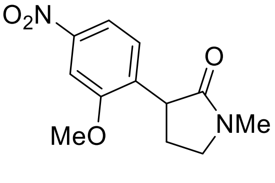

4a

400 MHz, $CDCl_3$

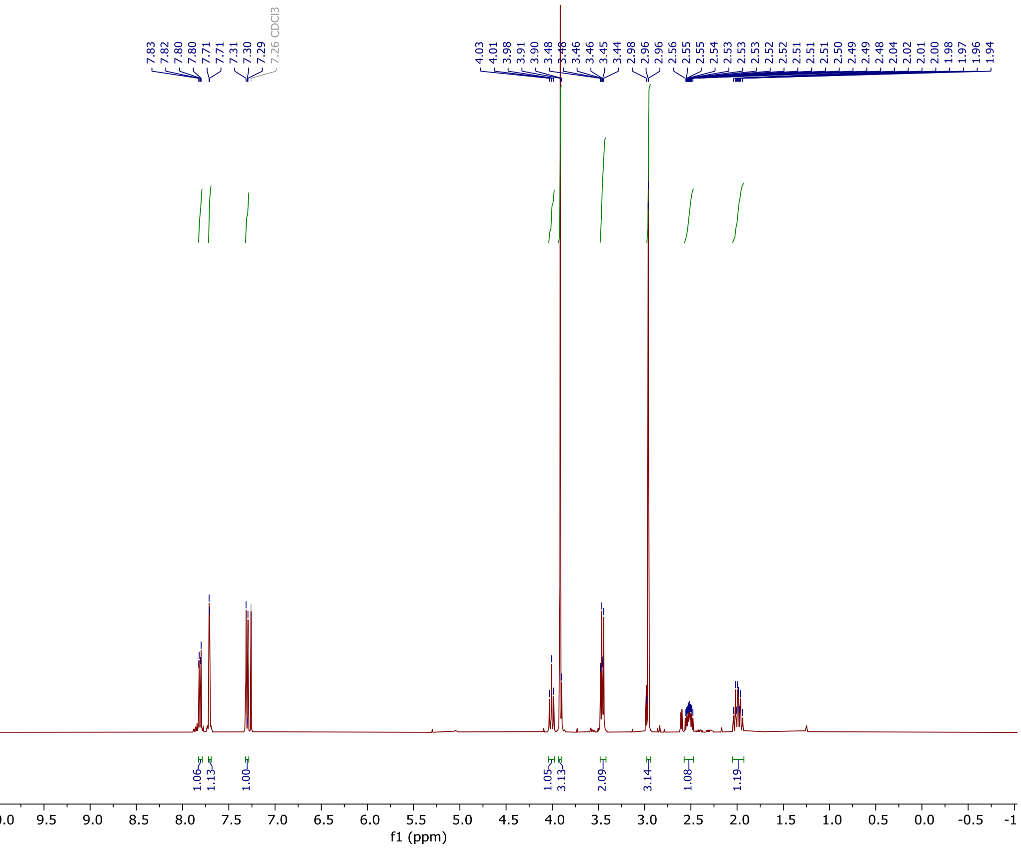

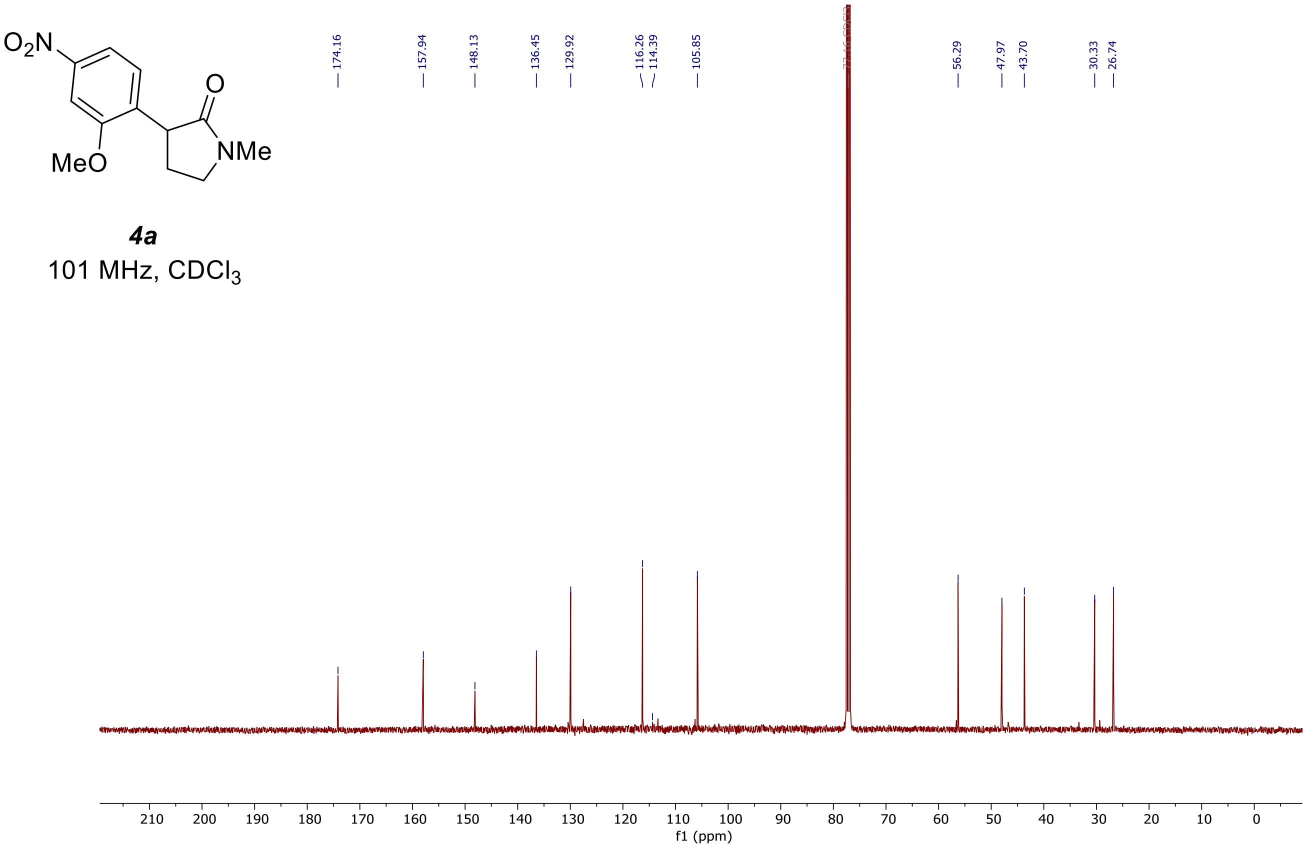

4a

101 MHz, $CDCl_3$

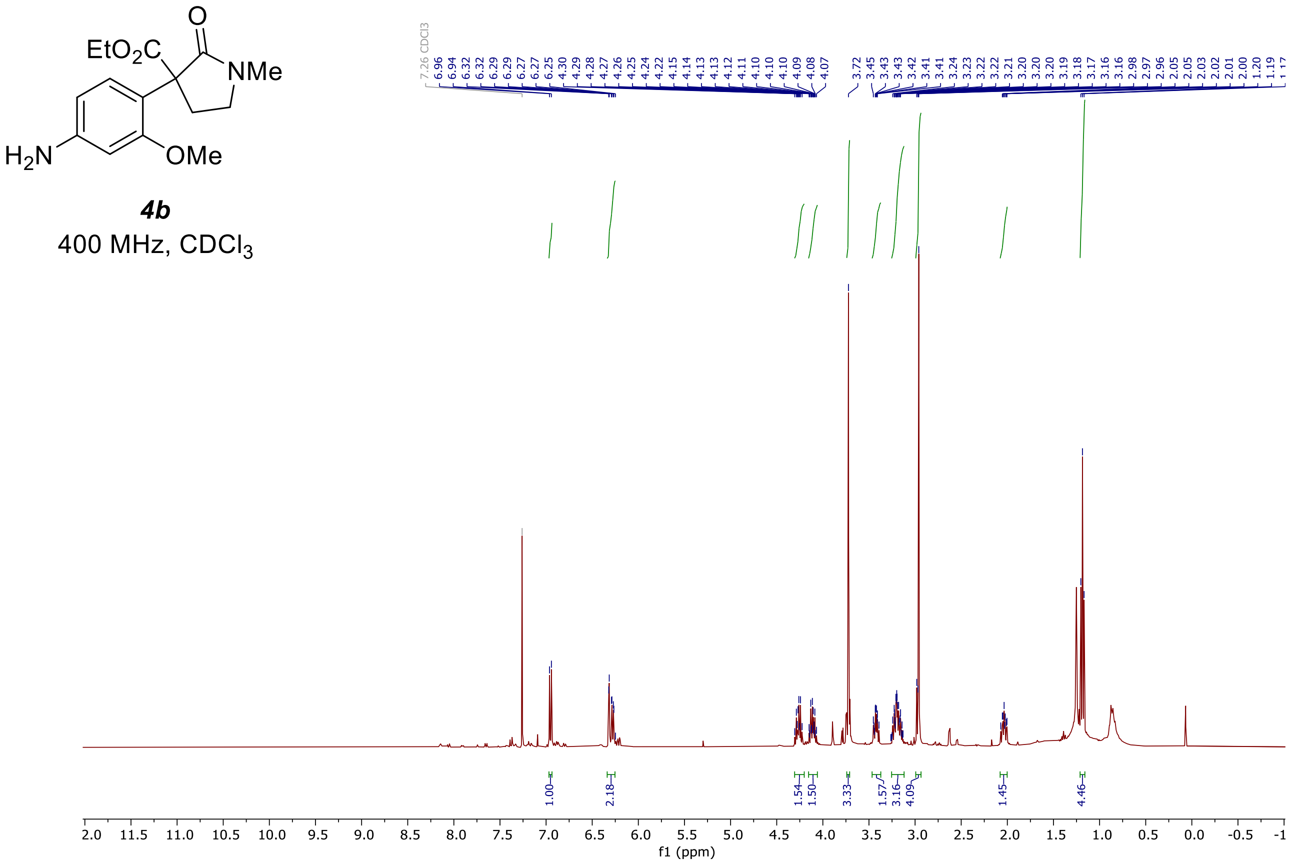

4b

400 MHz, $CDCl_3$

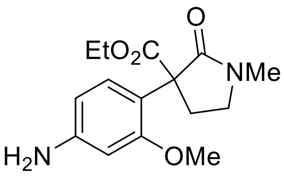

4b
101 MHz, $CDCl_3$

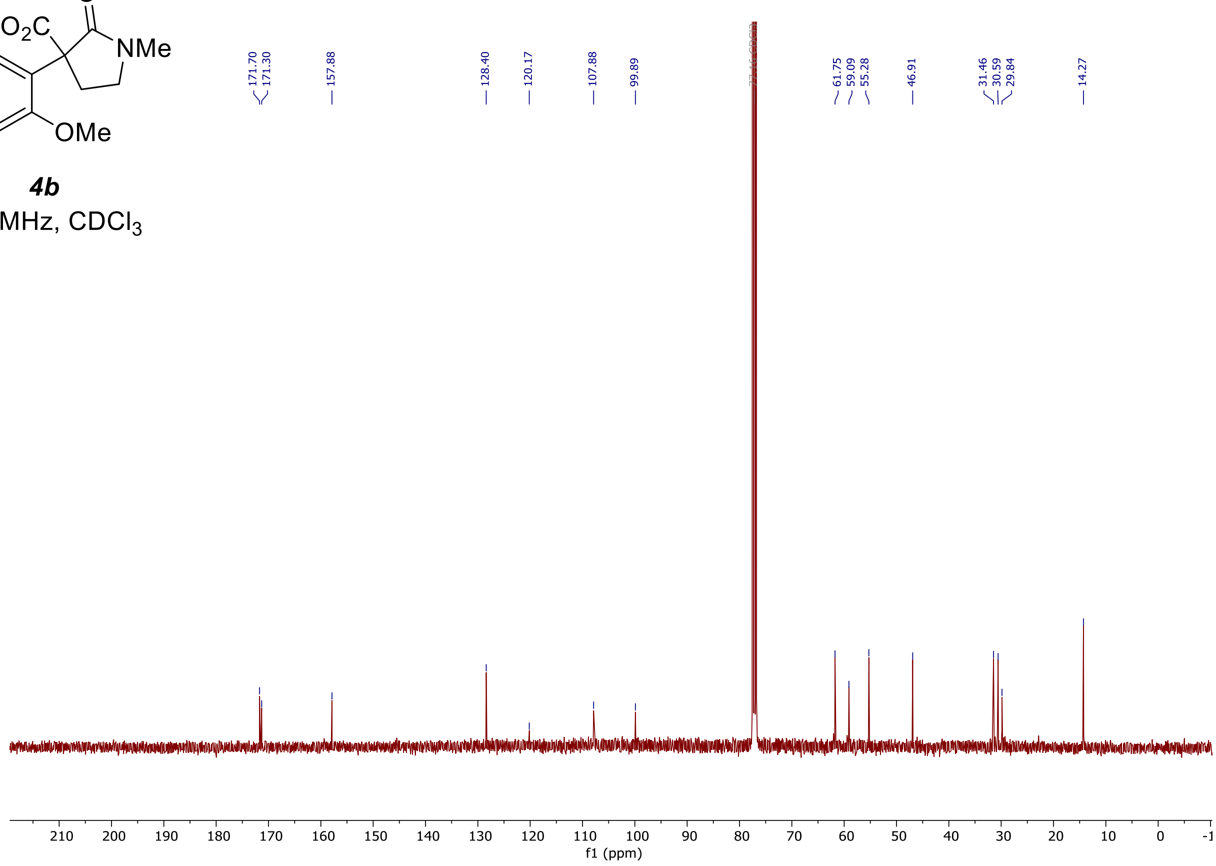

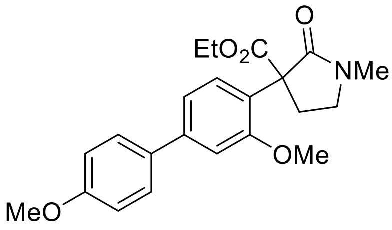

4c
400 MHz, $CDCl_3$

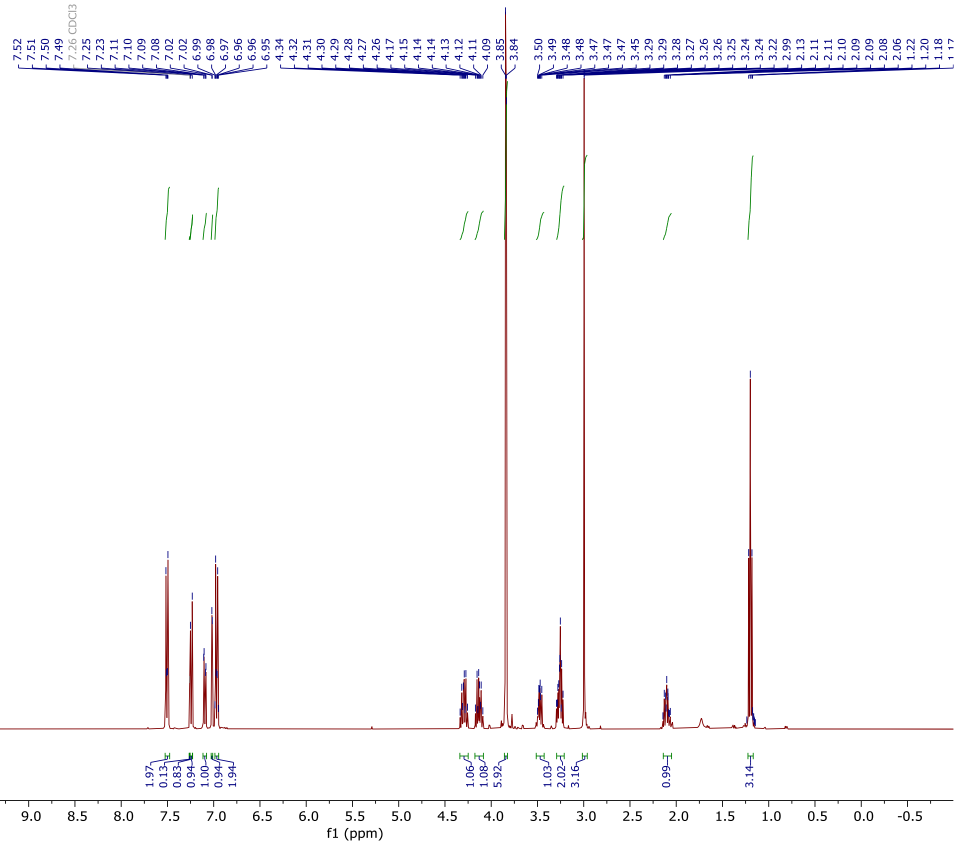

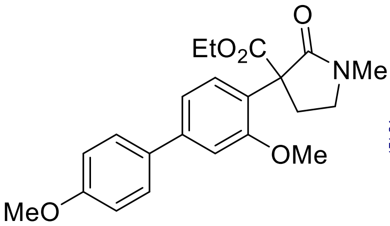

4c

101 MHz, $CDCl_3$

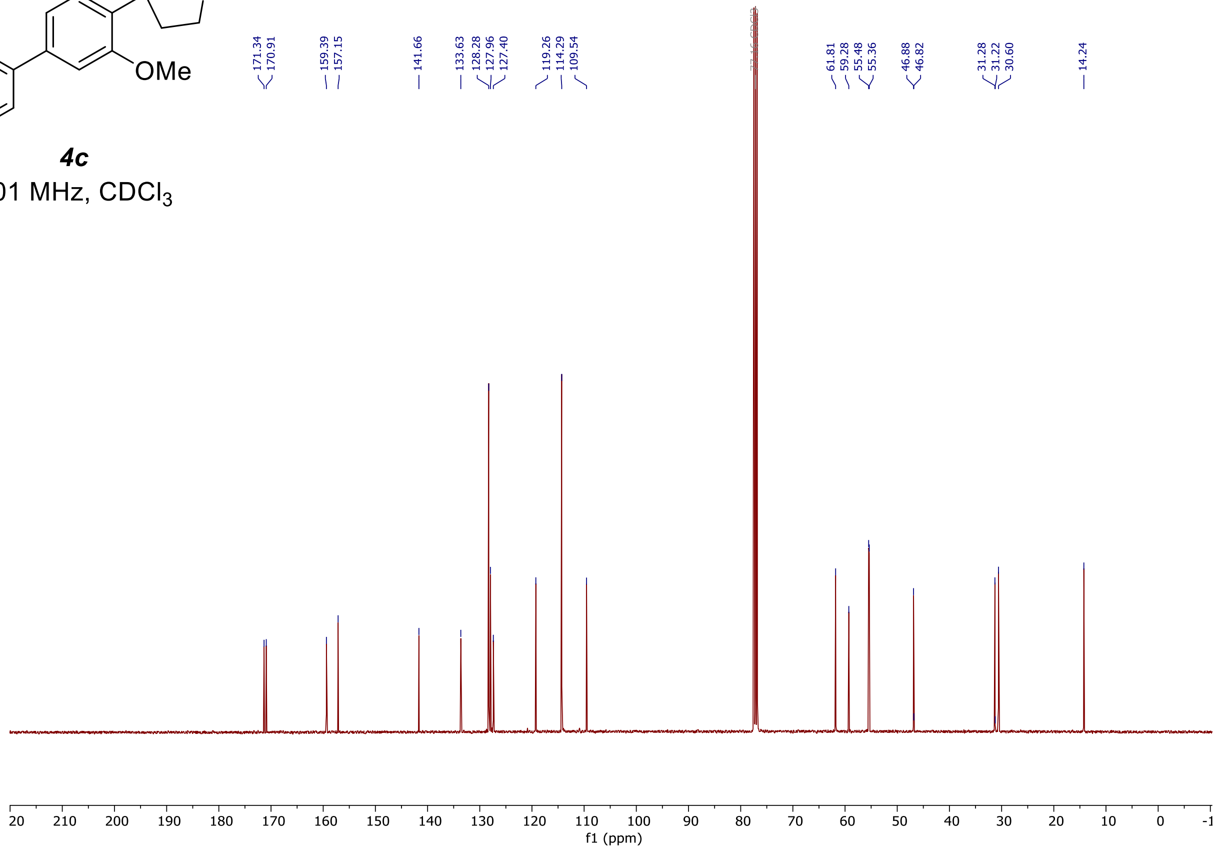

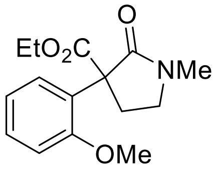

4d

400 MHz, $CDCl_3$

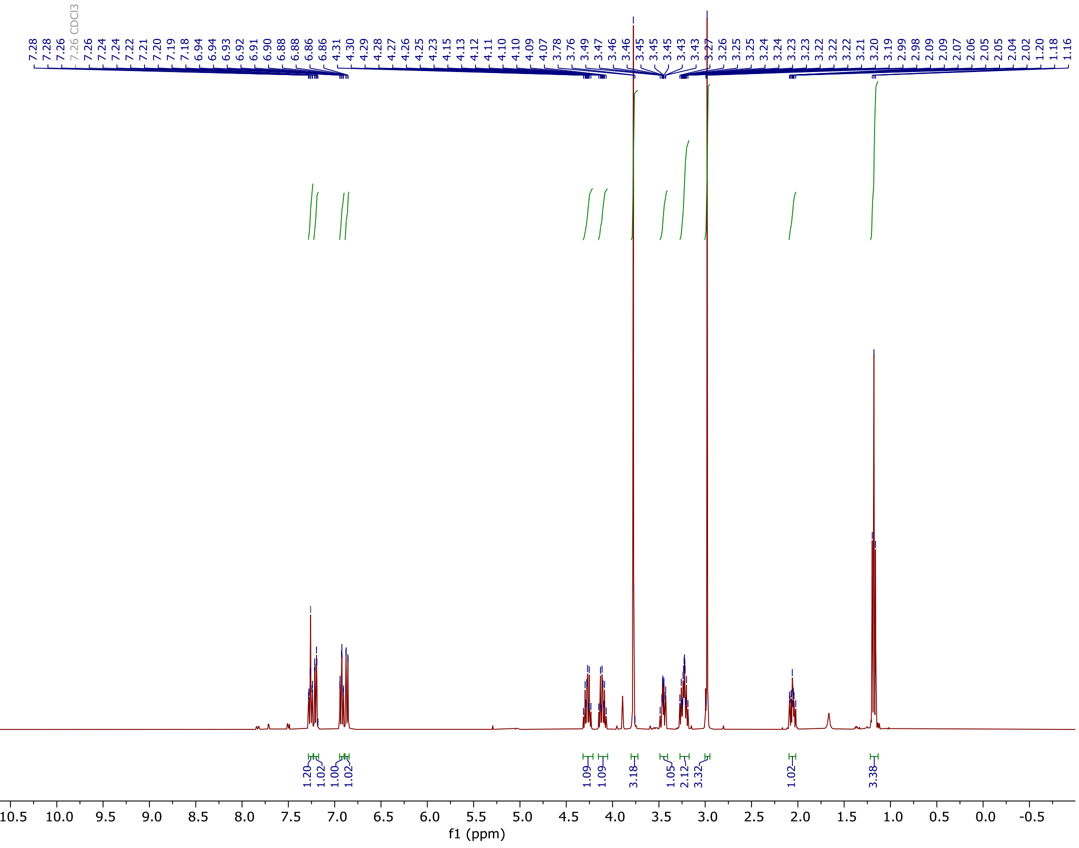

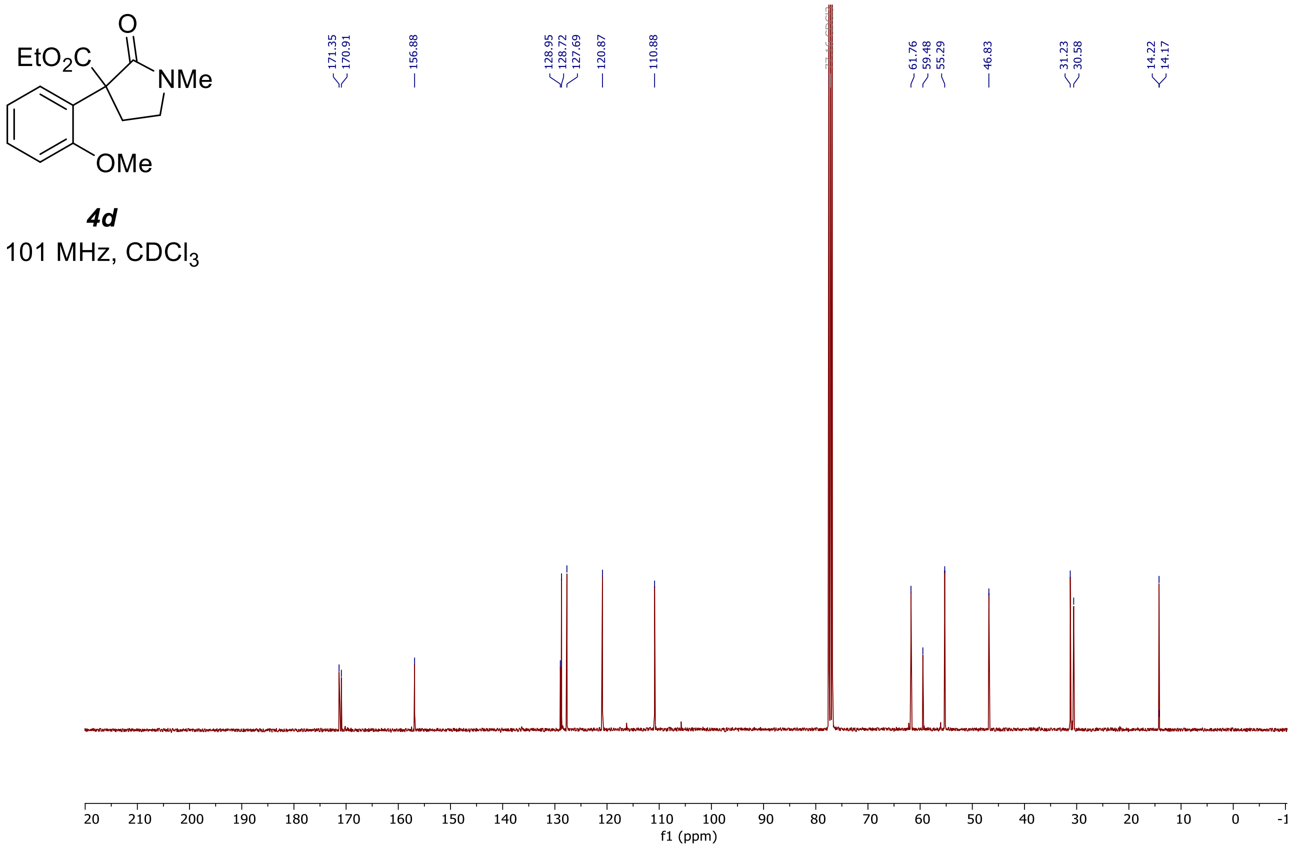

4d

101 MHz, $CDCl_3$

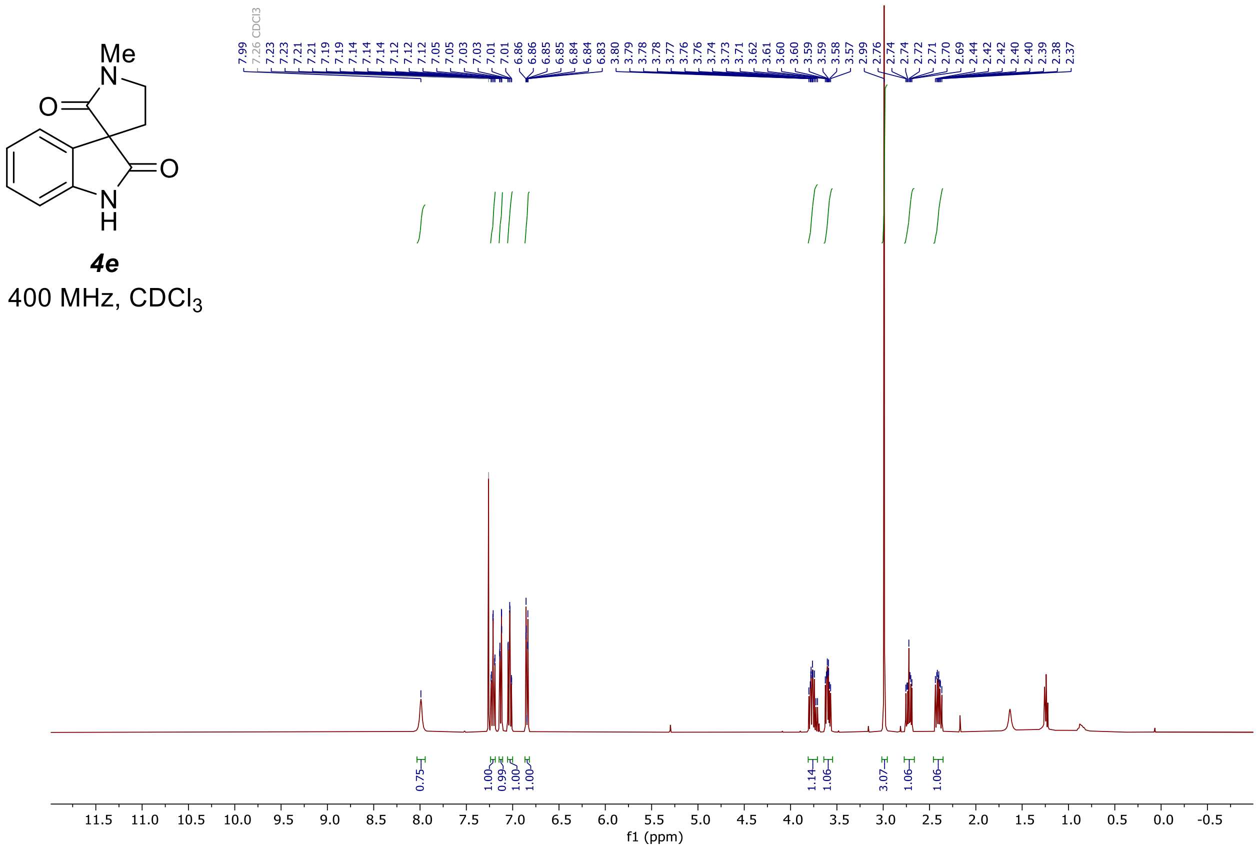

4e

400 MHz, $CDCl_3$

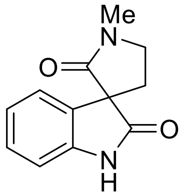

4e

101 MHz, $CDCl_3$

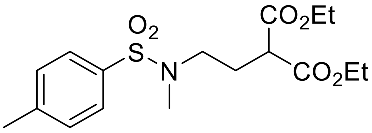

5a

400 MHz, $CDCl_3$

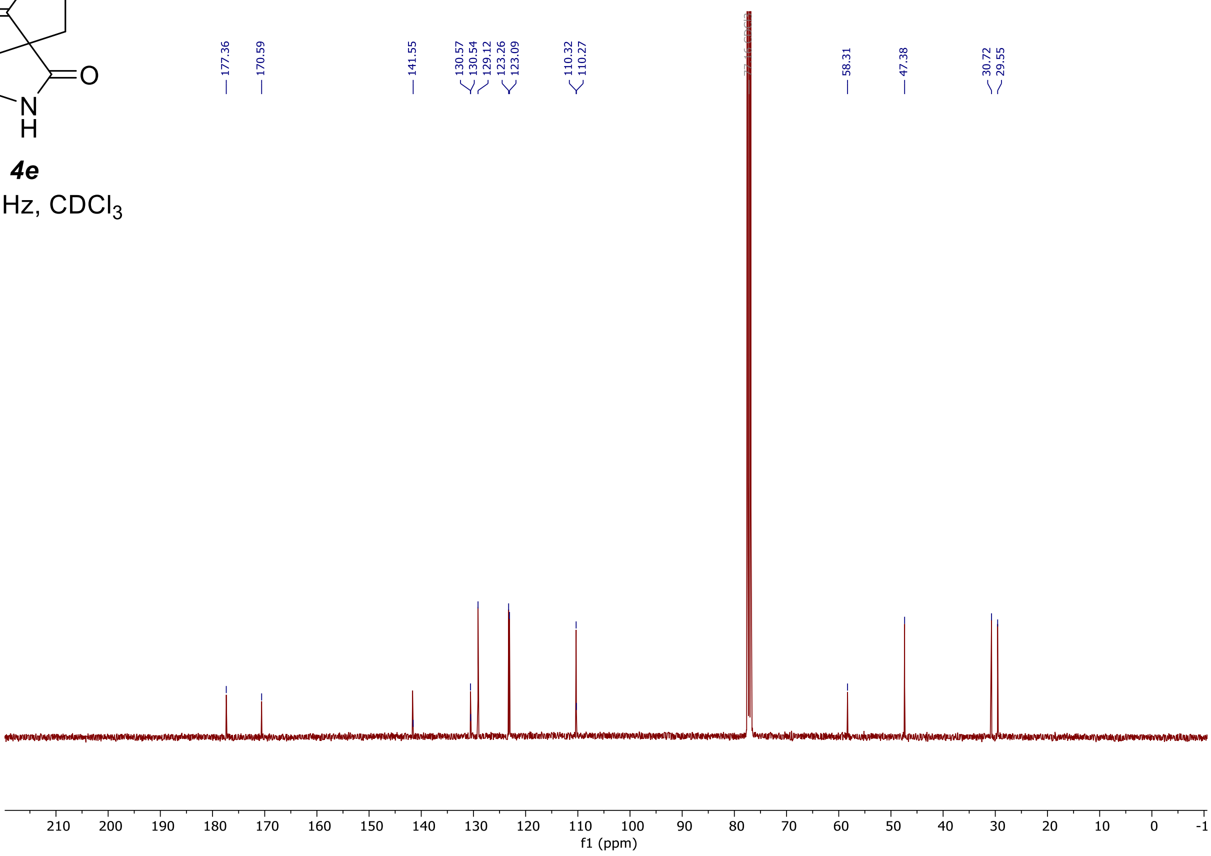

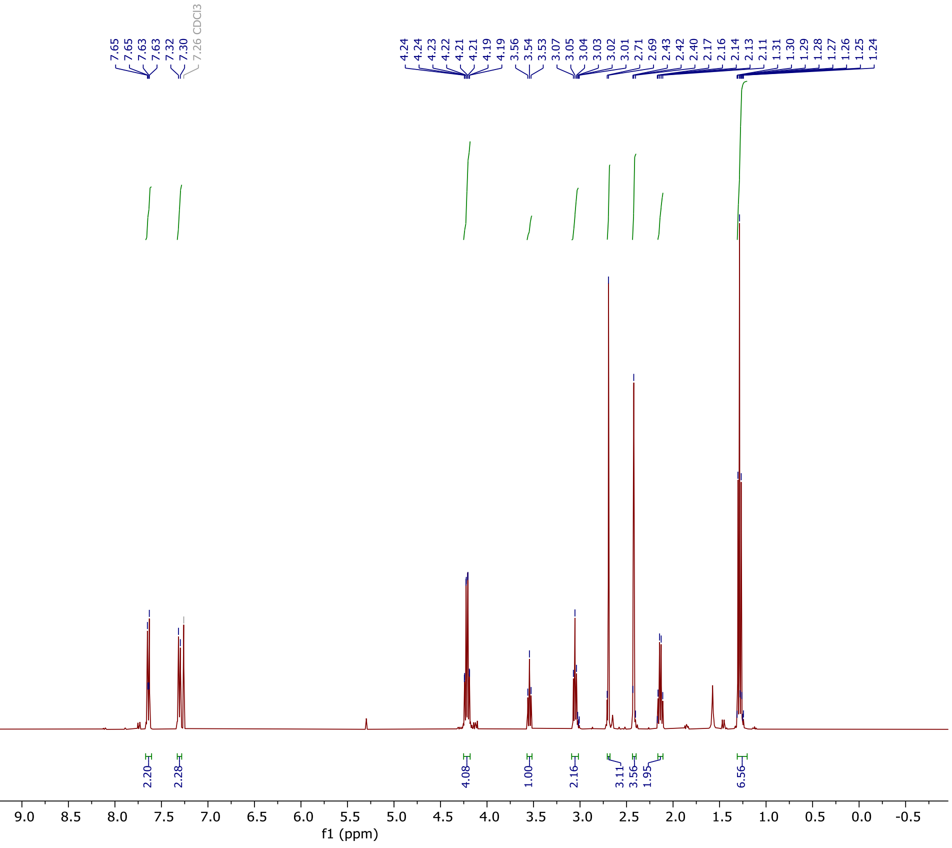

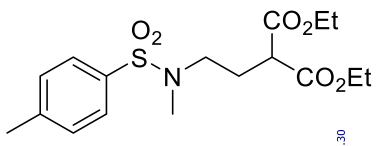

5a

101 MHz, $CDCl_3$

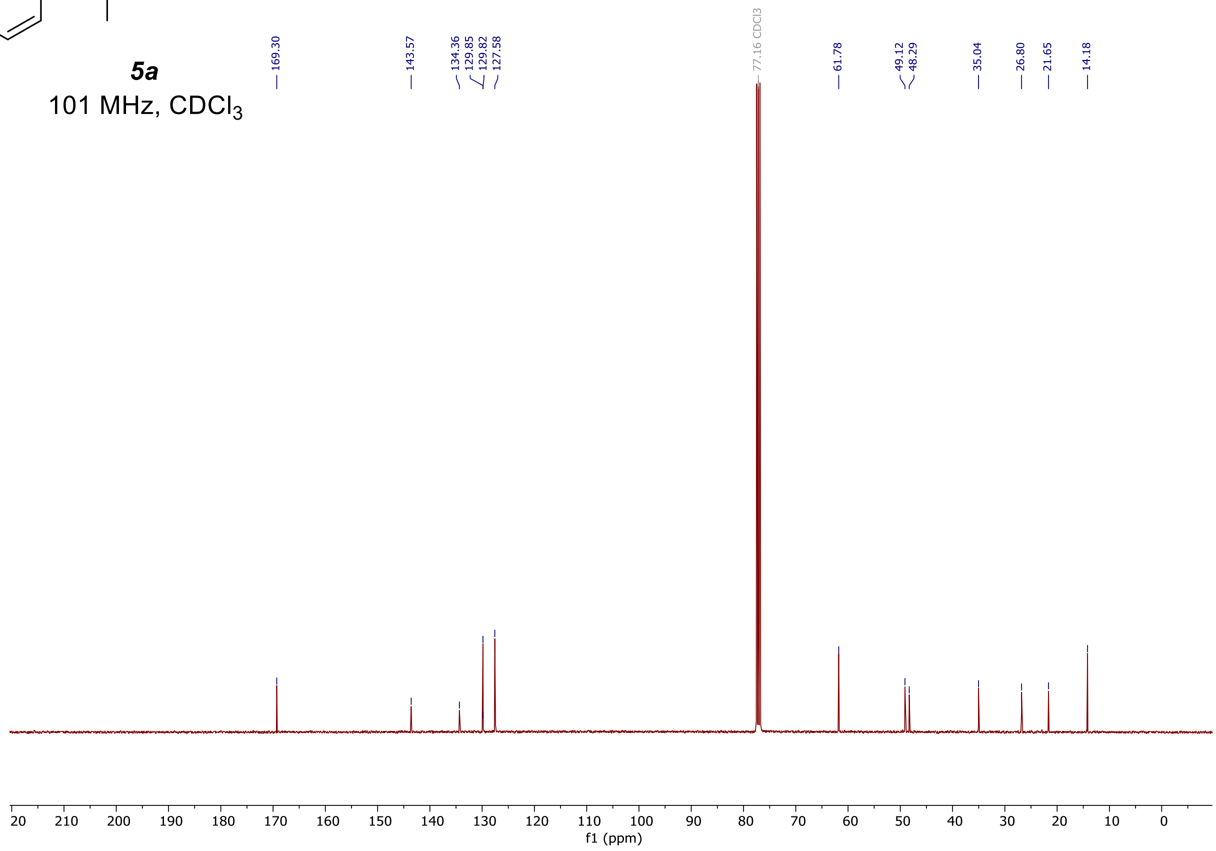

7. References

1 Z. Chen, A. Y. Hong and X. Linghu, *J. Org. Chem.*, 2018, **83**, 6225–6234.

2 S. Johnson, E. Kovács and M. F. Greaney, *Chem. Commun.*, 2020, **56**, 3222–3224.

3 C. Hirschhäuser, J. S. Parker, M. W. D. Perry, M. F. Haddow and T. Gallagher, *Org. Lett.*, 2012, **14**, 4846–4849.

4 J. J. Dotson, I. Liepuoniute, J. L. Bachman, V. M. Hipwell, S. I. Khan, K. N. Houk, N. K. Garg and M. A. Garcia-Garibay, *J. Am. Chem. Soc.*, , DOI:10.1021/jacs.1c01100.

5 M. R. Yadav, M. Nagaoka, M. Kashihara, R.-L. Zhong, T. Miyazaki, S. Sakaki and Y. Nakao, *J. Am. Chem. Soc.*, 2017, **139**, 9423–9426.

6 M. Kashihara, M. R. Yadav and Y. Nakao, *Org. Lett.*, 2018, **20**, 1655–1658.

7 M. G. Kulkarni, A. P. Dhondge, S. W. Chavhan, A. S. Borhade, Y. B. Shaikh, D. R. Birhade, M. P. Desai and N. R. Dhatrak, *Beilstein J. Org. Chem.*, 2010, **6**, 876–879.
